# Metabolic Changes in *Pseudomonas oleovorans* Isolated from Contaminated Construction Material Exposed to Varied Biocide Treatments

**DOI:** 10.3390/metabo14060326

**Published:** 2024-06-10

**Authors:** Muatasem Latif Ali, Lionel Ferrieres, Jana Jass, Tuulia Hyötyläinen

**Affiliations:** 1 School of Science and Technology, Örebro University, Fakultetsgatan 1, SE 701 82 Örebro, Sweden; muatasem.latif@oru.se (M.L.A.); jana.jass@oru.se (J.J.); 2 Saint-Gobain SWEDEN AB, SCANSPAC, Kemivägen 7, SE 705 97 Glanshammar, Sweden; 3 Saint-Gobain Recherche, 39 Quai Lucien Lefranc, FR-93303 Aubervilliers Cedex, France; lionel.ferrieres@saint-gobain.com

**Keywords:** biocides, *Pseudomonas oleovorans*, metabolomics, biocides resistance, 2-Methylisothiazol-3(2H)-one, 1,2-Benzisothiazol-3(2H)-one, 5-chloro-2-methyl-isothiazol-3-one

## Abstract

Biocide resistance poses a significant challenge in industrial processes, with bacteria like *Pseudomonas oleovorans* exhibiting intrinsic resistance to traditional antimicrobial agents. In this study, the impact of biocide exposure on the metabolome of two *P. oleovorans* strains, namely, *P. oleovorans* P4A, isolated from contaminated coating material, and *P. oleovorans* 1045 reference strain, were investigated. The strains were exposed to 2-Methylisothiazol-3(2H)-one (MI) MIT, 1,2-Benzisothiazol-3(2H)-one (BIT), and 5-chloro-2-methyl-isothiazol-3-one (CMIT) at two different sub-inhibitory concentrations and the lipids and polar and semipolar metabolites were analyzed by ultra-high performance liquid chromatography quadrupole time-of-flight mass spectrometry UPLC–Q–TOF/MS. Exposure to the BIT biocide induced significant metabolic modifications in *P. oleovorans*. Notable changes were observed in lipid and metabolite profiles, particularly in phospholipids, amino acid metabolism, and pathways related to stress response and adaptation. The 1045 strain showed more pronounced metabolic alterations than the P4A strain, suggesting potential implications for lipid, amino acid metabolism, energy metabolism, and stress adaptation. Improving our understanding of how different substances interact with bacteria is crucial for making antimicrobial chemicals more effective and addressing the challenges of resistance. We observed that different biocides trigged significantly different metabolic responses in these strains. Our study shows that metabolomics can be used as a tool for the investigation of metabolic mechanisms underlying biocide resistance, and thus in the development of targeted biocides. This in turn can have implications in combating biocide resistance in bacteria such as *P. oleovorans.*

## 1. Introduction

Biocides are chemical substances designed to inhibit or kill a wide range of microorganisms, including bacteria, fungi, algae, and viruses, in diverse industrial settings. They are especially important in water-based industrial products, such as those in the construction industry [1]. While their usage is crucial for preserving the integrity and aesthetics of coated surfaces, their indiscriminate deployment can lead to adverse consequences for both the environment and human health. The limitations associated with biocide dosage in coating materials encompass environmental concerns, health hazards, and compliance with regulatory frameworks [2].

Biocides are used worldwide for an increasing number of applications despite the tightening regulations in Europe and the United States. Numerous nations have enacted stringent regulations and guidelines governing the application of biocides in coatings to mitigate their environmental and health repercussions [3]. 

Non-adherence to these mandates can result in legal ramifications. Biocides are widely utilized in various industrial and consumer products. However, their extensive use has raised significant health and environmental concerns. Among these biocides, CMIT, MIT, and BIT have been particularly scrutinized. CMIT is known for its genotoxic and toxic properties, which can have trans- and multigenerational effects [1,4]. MIT is commonly associated with allergic reactions and skin sensitization. Its presence in cosmetic products has raised public health concerns, prompting restrictions on its use [5]. Exposure to MIT can result in dermatitis and other skin issues [6]. Similarly, BIT is known to cause allergic reactions and skin sensitization. Despite its stability up to pH 14 and low volatility, BIT can irritate the skin, eyes, and respiratory system [7].

Moreover, the emergence of biocide resistance poses a significant threat to the long-term effectiveness of these materials [8]. New regulations force industries to comply with minimized doses of biocide in their products, with ramifications on product quality and potentially increasing biocide resistance. Thus, understanding the metabolism of the target microbes will help in developing more efficient and sustainable preservation agents.

Contaminating microorganisms are responsible for the degradation of coating materials, which leads to significant losses of revenue and time for the industry [9]. Water-based coating materials produced from raw non-sterile materials are particularly susceptible to microbial deterioration due to the availability of nutrients supporting microbial growth. Product spoilage in coating materials is a significant concern in various industrial settings, with microorganisms, such as bacteria, fungi, and yeasts, often playing a key role in these incidents. Microbial growth modifies the coating viscosity, color, odor, and pH and may also produce visible surface growth. The pH of the product impacts microbial growth; for instance, bacteria tend to thrive in neutral to slightly alkaline pH conditions. Consequently, the pH of water-based coating materials assumes a pivotal role in shaping antimicrobial efficacy. It is noteworthy that specific bacterial species, such as those belonging to the *Pseudomonas* genus, experience restricted growth and reproduction in alkaline environments. This limitation is typically observed within the pH range of 8 to 9.5 [10].

In general, products often incorporate biocide agents, such as BIT, MIT, and CMIT, to combat a wide range of microbes. However, the effectiveness of these biocides in preventing microbial growth is contingent upon their specific chemistry, inherent properties, and mode of action [1], but the mechanism of action of different biocides is still not fully characterized [11]. By understanding how biocides interact with bacterial cells, what changes they cause in the bacteria, and how bacteria might develop resistance at a molecular level, better and more targeted antimicrobial strategies can be developed. *P. oleovorans* P4A isolated from contaminated coating material is a versatile hydrocarbon-degrading bacterium that is involved in water-based product degradation. It is a Gram-negative bacterium known for its remarkable ability to degrade and metabolize a wide range of hydrocarbons, including aliphatic and aromatic compounds in diverse environmental conditions [11,12]. The metabolic versatility of *P. oleovorans* is a notable feature attributed to its diverse array of catabolic pathways and enzymes, specifically designed for the degradation of hydrocarbons [12]. This bacterium has demonstrated remarkable adaptability to various environmental conditions, particularly those contaminated with hydrophobic compounds such as oil and related hydrocarbons [13]. Previous studies have highlighted the impressive catabolic capabilities of *Pseudomonas* ssp., highlighting its proficiency in utilizing a wide range of hydrocarbons as carbon and energy sources [14]. The bacterium employs an array of enzymes, including hydroxylases, dioxygenases, and dehydrogenases, which play roles in the initial steps of hydrocarbon degradation [15]. These enzymes enable the breaking down of complex hydrocarbon structures into metabolically usable intermediates, facilitating their incorporation into central metabolic pathways [16]. Plasmid-mediated resistance genes carried on plasmids can be transferred between Gram-negative bacteria, facilitating the spread of biocide resistance. Previous studies have explored the resistance mechanisms of various *Pseudomonas* species to biocides [17]. *Pseudomonas* species, including *P. oleovorans*, can exhibit varying degrees of resistance to biocides and this resistance can be attributed to factors including efflux pumps, mutation, adaptation, and biofilm formation [18,19,20]. *Pseudomonas* species possess an array of efflux pumps that actively pump out biocides from the cell, reducing efficacy. It can also develop mutations that render these pumps less susceptible to biocides over time [18]. The type of mutation that can render microorganisms less susceptible to biocides over time is often referred to as antimicrobial resistance (AMR) [19]. Antimicrobial resistance occurs when microorganisms, such as bacteria, undergo genetic changes that enable them to survive exposure to the biocidal agents that were once effective against them [20,21]. *Pseudomonas* species are notorious for their ability to form biofilms, which provide a protective matrix and resist the effects of biocides [22,23].

Biocides exposure influences the physiology of the organism, and this should be reflected at the metabolome and proteome level. *P. oleovorans* is known for its capacity to metabolize lipids, known for their role in many biological systems. Lipids represent a diverse group of organic molecules that are an important energy source, maintaining the cell structure and cell membrane integrity, as well as being involved in the cellular signaling pathways [24]. The relationship between lipid metabolism and biocide exposure is complex. Lipids play a crucial role in various cellular functions, including maintaining cell structure, serving as energy storage, and participating in signaling pathways [25]. When microbes are exposed to various types of biocides, this can lead to alterations in metabolism. This response is due to the multi-targeted antimicrobial action of biocides, which can affect the cellular structures and functions of microorganisms [26,27]. These changes are often indicative of the organism’s response to stress or environmental disturbance [28]. Several studies have reported alterations in lipid metabolism following exposure to biocides. For instance, some biocides can disrupt cell membranes by interacting with lipid components, leading to changes in membrane fluidity and permeability [1,25]. Additionally, biocides may induce oxidative stress, which can affect lipid peroxidation and impact lipid homeostasis [29]. Related to *Pseudomonas* strains, there is not yet much data on the impact of biocides on lipid metabolism, or metabolism in general. To understand the metabolic pathways affected by biocide treatment, a comprehensive metabolomics characterization is needed, including lipids as well as other metabolites. Changes in metabolite profiles can also serve as biomarkers for specific metabolic pathways affected by biocide exposure. By analyzing a broad spectrum of metabolites, we can gain insights into the overall impact on cellular metabolism and identify potential targets for further investigation. Studying metabolism in the context of biocide exposure, especially in *Pseudomonas* strains, is crucial for understanding the adaptive responses of these organisms to environmental stress. 

*P. oleovorans* possess enzymes and pathways for fatty acid degradation, including fatty acid β-oxidation, a central process in breaking down fatty acids into acetyl-CoA units, which can be further catabolized for energy production [30,31]. 

In this study, we investigated the metabolic effects of three different biocides on *P. oleovorans* P4A, a biocide-resistant industrial isolate and 1045 reference strain, to understand the metabolic mechanism of biocide resistance in these strains. Specifically, this bacterial strain has been identified in industrial products and it has shown strong resistance against biocides, and thus it can cause degradation of industrial products. To enable the development of more efficient biocide preservation agents, it is important to understand the molecular mechanism underlying the biocide resistance. Thus, comprehensive metabolic profiling was applied to investigate the effects of exposure to three different mixtures of biocides commonly used in industry, namely MIT, BIT, and CMIT. These biocides, which are widely used in industry, have different properties; MIT is stable and effective against bacteria, BIT is stable up to pH 14 and has low volatility, while CMIT has low solubility and is effective against fungi. We then compared the metabolic changes triggered by these biocides at the metabolic pathway level [1]. 

The objective of this study was to examine the metabolic effects of biocides (CMIT, MIT, and BIT) on two strains of *P. oleovorans*, particularly within the context of industrial water-based products. The main goal is to gain an understanding of the metabolism of target microbes that could be later utilized in the development of more efficient and sustainable preservation strategies. A particular focus was on *P. oleovorans* strain P4A. We compared the metabolome and the response to biocide exposure of the P4A strain with *P. oleovorans* strain DSM 1045, which was isolated from industrial cutting fluid in the USA in 2009 and has been reported to be biocide-sensitive [32]. 

## 2. Materials and Methods

### 2.1. Chemicals

CMIT, MIT, and BIT were purchased from Avantor VWR. The purity of the chemicals used in the study was as follows: CMIT (≥98% purity), MIT (≥98% purity), and BIT (≥98% purity). Mass spectrometry grade ammonium acetate and reagent grade formic acid were obtained from Sigma-Aldrich (St. Louis, MO, USA), while all solvents used were of HPLC or LC–MS grade, sourced from Honeywell (Morris Plains, NJ, USA), Fisher Scientific (Waltham, MA, USA), or Sigma-Aldrich (St. Louis, MO, USA). Lipid standards were acquired from Avanti Polar Lipids Inc. (Alabaster, AL, USA).

For quality assurance purposes, we employed standard reference materials: serum SRM 1950 (for lipidomic and metabolomics) and SRM 1957 (for bile acids), both of which were obtained from the National Institute of Standards and Technology (NIST) at the US Department of Commerce (Washington, DC, USA) (NIST, 2023).

### 2.2. Biocide Exposure

*P. oleovorans* P4A, a biocide-resistant industrial isolate, and the reference strain *P. oleovorans* 1045 were streaked out from the −80 °C storage freezer onto Luria–Bertani (LB) plates and incubated at 30 °C for 48 h. LB was purchased from Avantor VWR, and the composition of the LB–Agar Lenox medium was 10.0 g Tryptone, 5.0 g Yeast Extract, 5.0 g Sodium Chloride, and 15.0 g Agar. 

**Figure 1:**
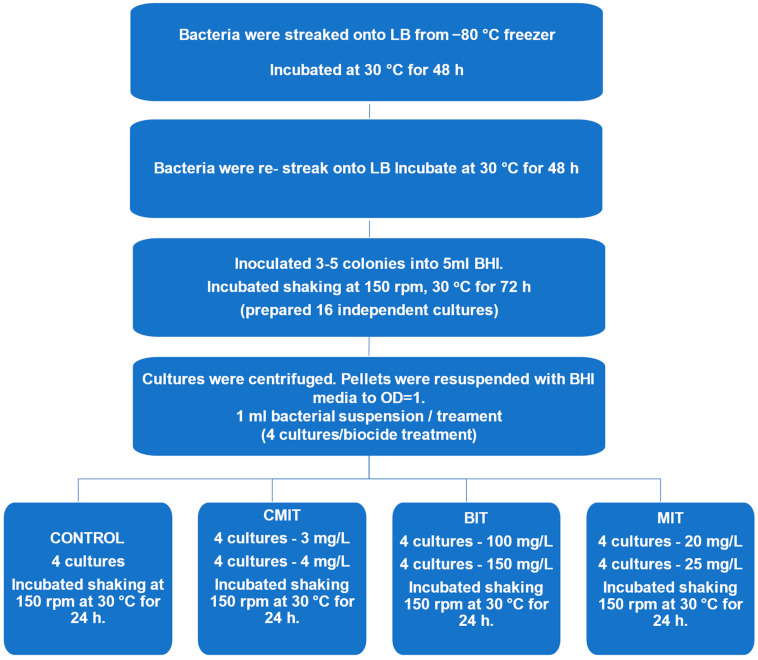
Workflow of culture preparation for lipidomic and metabolomic analysis. The 16 independent cultures prepared for each *Pseudomonas oleovorans* P4A and 1045 according to the flowchart were divided so that 4 cultures were used for each treatment condition. A 1 mL volume of OD-adjusted bacterial suspension ensured that equivalent biomass was used for each exposure and control.

The biocide exposure protocol, outlined in Figure 1, commenced with re-streaking bacterial cultures onto LB agar and incubating at 30 °C for 48 h. After incubation, three to five colonies were transferred to sixteen tubes containing 5 mL of Brain Heart Infusion (BHI) broth for each strain. BHI was Purchased from Avantor VWR; the composition of the media was 7.7 g Brain infusion solids, 9.8 g Beef heart infusion solids, 10.0 g Peptones, 2.0 g Glucose, 5.0 g Sodium chloride, and 2.5 g Disodium hydrogen phosphate. The tubes were incubated with shaking at 150 rpm for 24 h at 30 °C, the resulting culture was centrifuged at 6000 rpm and the pellet was adjusted to an optical density (OD) of 1 at 600 nm with fresh BHI. The spectrophotometer Infinite F50 Microplate Reader, Tecan, used for optical density (OD) measurements was calibrated daily to maintain accuracy. OD readings were taken at a wavelength of 600 nm to evaluate bacterial growth. Although the bacteria reached a stationary phase in the initial growth, they were pelleted and resuspended in fresh BHI up to OD = 1. Using fresh media will allow the cultures to grow, therefore the exposure was in an exponential phase and by 24 h would have likely reached a stationary status. To achieve standardization of bacterial culture to an optical density (OD) of 1 at 600 nm, bacterial culture was initiated in the Brain Heart Infusion (BHI) broth medium and incubated until it entered the logarithmic phase of growth. The spectrophotometer was calibrated to zero absorbance using a blank cuvette filled with the growth medium. A small sample of the bacterial culture was obtained, and its initial OD at 600 nm was determined using the spectrophotometer. The OD measurement for the diluted culture was repeated, and further dilution adjustments were made as needed until the desired OD was achieved. The dilution factor required to attain an OD of 1 was calculated by dividing the target OD by the initial OD. Subsequently, the culture was diluted in a new tube with fresh medium, ensuring comprehensive mixing. To validate the OD measurements, diluted samples were plated on agar plates, and the colony-forming units were enumerated for accuracy. The final OD of the adjusted culture, along with details of any dilution steps undertaken, was recorded to ensure the reproducibility of experiments.

To investigate the effects of biocide exposure on both strains, we conducted exposures at concentrations below their respective Minimum Inhibitory Concentrations (MICs). Our methodological strategy diverges from conventional Minimum Inhibitory Concentration (MIC) determinations. Unlike MIC assays that require higher concentrations to evaluate antimicrobial efficacy, our approach focuses on sub-MIC levels. This allows for a more detailed understanding of biocide effects on lipidomic profiles while avoiding potential confounding factors associated with supra-MIC exposures [33,34,35].

Four cultures of the P4A strain and four cultures of the 1045 strain served as untreated controls, with each set considered as four replicates for their respective strain. Each of the twelve remaining tubes for each strain was further split into two fresh tubes, resulting in a total of 24 tubes for each strain. Within each set of two tubes derived from the same original tube, 1 mL of inoculum from each tube was exposed to CMIT. Specifically, one tube was exposed to a concentration of 3 mg/L CMIT, and the other tube was exposed to a concentration of 4 mg/L CMIT. This process was replicated four times for each concentration of CMIT, resulting in a total of eight tubes being exposed to CMIT. To break this down further, four tubes were exposed to 3 mg/L CMIT, and another set of four tubes was prepared by exposing 1 mL of inoculum from these four tubes to 4 mg/L CMIT (Figure 1). 

This exposure procedure was repeated for MIT, with concentrations of 20 ppm and 25 ppm, and BIT, with concentrations of 100 ppm and 150 ppm. In summary, a total of 28 samples were prepared for each strain, resulting in 56 samples overall. All samples were then incubated for 24 h at 150 rpm and 30 °C.

Following incubation, samples were harvested by centrifugation at 6000 rpm for 10 min. The supernatant was collected and stored for subsequent analysis. Bacterial pellets were washed with phosphate-buffered saline (pH 7.4), vortexed, and centrifuged at 6000 rpm for 10 min and frozen at −80 °C for future analysis. All samples were maintained at −80 °C until further use. 

### 2.3. LC–MS Analysis

Sample Preparation and Analysis: The samples were analyzed in randomized order. The bacterial cell samples were weighed, and phosphate-buffered saline (PBS) was added to achieve a ratio of 1 mg of bacterial mass to 10 µL of buffer. The resulting samples were vortexed and then ultrasonicated for 3 min. Two distinct extraction methods were employed in this study: one for lipidomic analysis and another for the extraction of polar and semipolar metabolites (Table 1). The samples were analyzed using ultra-high-performance liquid chromatography quadrupole time-of-flight mass spectrometry (UHPLC–QTOFMS) equipped with dual ESI ionization. The UHPLC system utilized in this study was a 1290 Infinity II system from Agilent Technologies, equipped with a multi-sampler, quaternary solvent manager, and a column thermostat maintained at 50 °C. Injection volumes were set to 1 µL, and separations were performed on an ACQUITY UPLC^®^ BEH C18 column (2.1 mm × 100 mm, particle size 1.7 µm) by Waters (Milford, MA, USA). The mass spectrometer, a 6545 QTOF from Agilent Technologies, was coupled to the UHPLC and operated in positive ion mode. MassHunter B.06.01 software (Agilent Technologies) was employed for all data acquisition, and quality control measures were consistently implemented throughout the dataset. Identification of compounds was performed by using in-house constructed databases (retention times, spectral data) for polar and semipolar metabolites and lipids, respectively. These spectral databases have been constructed using authentic standards and, for those lipids, those standards were not available, based on their mass spectrum and retention behavior.

A. Lipidomic Analysis: for lipidomic analysis, a modified version of the Folch procedure [36] was used to optimize the extraction of the lipids from biological samples [37]. A total of 20 µL of the cell homogenate was extracted with 150 µL of a 2.5 ppm internal standard solution in CHCl_3_: MeOH (Chloroform: methanol). The standard solution contained the following compounds: 1,2-diheptadecanoyl-sn-glycero-3-phosphoethanolamine (PE(17:0/17:0)), N-heptadecanoyl-D-erythro-sphingosylphosphoryl-choline (SM (d18:1/17:0)), N-heptadecanoyl-D-erythro-sphingosine (Cer (d18:1/17:0)), 1,2-diheptadecanoyl-sn-glycero-3-phosphocholine (PC(17:0/17:0)), 1-heptadecanoyl-2-hydroxy-sn-glycero-3-phosphocholine (LPC(17:0)) and 1-palmitoyl-d31-2-oleoyl-sn-glycero-3-phosphocholine (PC (16:0/d31/18:1)), were purchased from Avanti Polar Lipids, Inc. (Alabaster, AL, USA), and, tri-heptadecanoyl-glycerol (TG(17:0/17:0/17:0)) was purchased from Larodan AB (Solna, Sweden) (Supplementary Table S1). After vortex mixing, the samples were incubated on ice for 30 min and then centrifuged for 5 min at 7800× *g*. Sixty µL from the lower layer of each sample was transferred to a glass vial with an insert, and 60 µL of CHCl_3_: MeOH (2:1, *v*/*v*) was added to each sample. These samples were stored at −80 °C until further analysis.

Calibration curves were generated using standard compounds, including PC(16:0e/18:1(9Z)), PC(18:0p/18:1(9Z)), LPC(18:0), LPC(18:1), PE(16:0/18:1), PC(18:0p/22:6), DG(18:0/18:2), LPE(18:1), Cer (d18:0/18:1 (9Z)), PE (16:0/18:1), LPC (16:0), TG (16:0/16:0/16:0), TG (18:0/18:0/18:0), ChoE (18:0), and ChoE (18:2) (Supplementary Table S2). These standards were prepared at various concentration levels (100, 500, 1000, 1500, 2000, and 2500 ng/mL in CHCl_3_: MeOH, 2:1, *v*/*v*), including 1250 ng/mL of each internal standard.

B. Polar, Semipolar Metabolites Analysis: For the analysis of polar metabolites, semipolar metabolites, and bile acids, a combined target–non-target method was employed. The initial step involved 80 µL of the homogenized cell samples extracted with 180 µL of acetonitrile (ACN) containing internal standards (13C-labeled PFOS, PFOA, PFDA, PFHxS, and PFUnDA, as well as Valine-d8, Glutamic acid-d5, Succinic acid-d4, Heptadecanoic acid, Lactic acid-d3, Citric acid-d4, 3-Hydroxybutyric acid-d4, Arginine-d7, Tryptophan-d5, Glutamine-d5, CA-d4, LCA-d4, UDCA-d4, CDCA-d4, DCA-d4, GCA-d4, GLCA-d4, GUDCA-d4, and GCDCA-d4), After vortexing and 3 min of ultrasonication, the samples were centrifuged for 5 min at 7800 rpm. A total of 180 µL was transferred to Vµ-vials, while 40 µL was reserved for pooled samples. The samples were evaporated to dryness and resuspended into 40 µL of 70% MeOH in water. 

The analysis of semipolar metabolites was performed using ultra-high-performance liquid chromatography quadrupole time-of-flight mass spectrometry (UHPLC–QTOFMS) from the same extract used for target analyses. The UHPLC system, Agilent 1290 Infinity II, was equipped with a multi-sampler, quaternary solvent manager, and a column thermostat set at 50 °C. A C18 column was employed for chromatographic separation, and the mass spectrometer, a 6545 QTOF from Agilent Technologies, operated in negative ion mode. Chromatographic conditions and ionization settings were optimized for semipolar metabolite analysis. MassHunter B.06.01 software was used for data acquisition.

Quality control measures, including extraction blanks, pure standard samples, pooled samples, and control serum samples, were integrated into the analysis process to ensure data accuracy and reliability.

### 2.4. Data Preprocessing

Mass spectrometry data preprocessing was conducted using MZmine 2.53, an open-source software package [38]. The following steps were applied:

1. Mass Detection: Mass detection utilized a noise level threshold of 750.
2. ADAP Chromatogram Building: Chromatograms were built with a minimum group size of 5, a group intensity threshold of 200, a minimum height of 1000, and an m/z tolerance of 0.007 m/z or 7 ppm.
3. Chromatogram Deconvolution: Deconvolution applied a 70% chromatographic threshold, 0.05 min minimum RT range, 5% minimum relative height, 1200 minimum absolute height, a minimum peak top/edge ratio of 1.2, and a peak duration range of 0.08–5.0.
4. Isotopic Peak Grouper: Isotopic peaks were grouped with an m/z tolerance of 5.0 ppm and an RT tolerance of 0.05 min, and the most intense isotope was selected as the representative.
5. Join Aligner: Data alignment used a m/z tolerance of 0.008 or 8 ppm and a weight of 2, RT tolerance of 0.15 min and a weight of 1, without requiring charge state or identification, and without comparing isotope patterns.
6. Peak List Row Filter: Rows in the peak list were filtered, requiring a minimum presence in 10% of the samples.
7. Gap Filling: Gap filling was conducted using the same RT and m/z range with an m/z tolerance of 0.009 m/z or 11.0 ppm.
8. Identification: Compounds were identified using in-house database searches with an m/z tolerance of 0.009 m/z or 10.0 ppm and a retention time tolerance of 0.2 min.

Quality Control: Quality control samples included aliquots of each sample, along with the NIST SRM1950 reference plasma sample and an in-house pooled serum sample. Relative standard deviations (% RSDs) for peak areas were computed, averaging 12.1% for lipidomic and 12.0% for polar metabolites.

### 2.5. Statistical Analysis

In the metabolomic data analysis, we employed the MetaboAnalyst 5.0 software [34]. Only lipids and metabolites detected in more than 70% of the samples were retained for analysis, ensuring a robust dataset. To prepare the metabolomics data for statistical analyses, a two-step preprocessing approach was applied. Firstly, the data underwent a logarithmic transformation, followed by auto-scaling. This transformation aimed to normalize the data and improve its suitability for subsequent statistical analyses.

Principal Component Analysis (PCA) was employed as an unsupervised method to extract underlying patterns for exploratory data analysis, and univariate analysis methods were employed. In the case of two-group data, such as Fold Change (FC) analysis, *t*-tests, and volcano plots (a combination of these two methods) were utilized. Significance was established when the *p*-value fell below the threshold of 0.05, and the false discovery rate was below 0.1. 

For multi-group data analysis, one-way analysis of variance (ANOVA) was conducted, followed by post-hoc analyses using Tukey’s the honest significant differences (HSD) test. These analyses were performed to generate a comprehensive list of potentially significant compounds that could differentiate between the conditions under investigation. Significance was established when the *p*-value fell below 0.05. 

Hierarchical clustering, combined with heatmap analysis, was used to provide a visual representation of a data table, with colored cells denoting concentration values. Rows usually represent samples, while columns represent features or compounds. This approach facilitates the identification of samples or features with notably high or low values, streamlining data analysis and pattern recognition processes.

We conducted pathway analysis using Metaboanalyst 5.0 [35] integrating pathway enrichment analysis and pathway topology analysis. This approach supports functional analysis of untargeted metabolomics data generated from high-resolution mass spectrometry. The pathway analysis was performed with the data of the polar and semipolar metabolites, as the pathway analysis for lipidomics data is not sufficiently robust due to the lack of exact structures of the lipids (fatty acid composition, including the position of the double bonds, cis/trans configuration). However, our polar/semipolar panel includes a large number of lipids, except for neutral lipids (CE, DG, TG), which are not covered either by sample preparation or the negative ion mode. The input data for the pathway analysis comprised complete LC–HRMS data, i.e., both identified and unknown metabolites, obtained in negative ionization mode, First, we performed statistical analyses using *t*-test between control and BIT-exposed strains, resulting in fold change, *p* values, and FDR values. The whole input peak list, with peak names given as their numeric mass (m/z) values for putative annotation, and the statistical results were used for the pathway analysis. We applied the Mummichog algorithm using the Kyoto Encyclopedia of Genes and Genomes (KEGG) database (KEGG) Pathway associated with *Pseudomonas putida* KT2440 to determine the relative significance of the identified pathways [38]. The mass tolerance for the pathway analysis was set at 7 ppm, and we also used the advanced option to select representative adducts by removing isotopic adducts, as these have been already removed in our data preprocessing step. This methodology facilitated the functional analysis of untargeted metabolomics data obtained from high-resolution mass spectrometry, enabling a comprehensive understanding of the metabolic pathways involved. For the main pathways identified as significant, we checked the metabolites that had been identified by the pathway analysis tool and performed additional MS/MS analyses for these compounds. 

## 3. Results

We exposed the two *Pseudomonas oleovorans* strains to sub-minimum inhibitory concentrations (MICs) of biocides, including 4 mg/L CMIT, 25 mg/L MIT, and 150 mg/L BIT. The biocide concentrations selected for the experiment were sub-MIC to ensure maximum exposure while obtaining sufficient biomass for the study. These concentrations were to affect various cellular processes without inhibiting growth. Two different concentrations were used to assess the metabolic changes due to biocide exposure, aiming to identify a concentration that yielded more significant effects. However, analysis revealed no substantial differences in metabolites between the two concentrations for all biocides tested. Consequently, samples were exposed to a higher concentration for further investigation. We applied different growth times for the two strains as the growth of P4A was considerably slower and reached the stationary phases after 35 h in comparison to the 1045, which reached the stationary phase after 18 h.

This choice aimed to permit bacterial growth while triggering potential survival mechanisms in the biocide-exposed environment. These mechanisms may include alterations in lipid composition and metabolite profiles, representing an adaptive response to sub-MIC biocide exposure. The changes in various lipids and metabolites facilitate the bacteria’s effective adaptation and thriving under the selective pressure of biocide exposure.

### 3.1. Lipid and Metabolite Profiles in Untreated P. oleovorans P4A and P. oleovorans 1045

**Figure 2:**
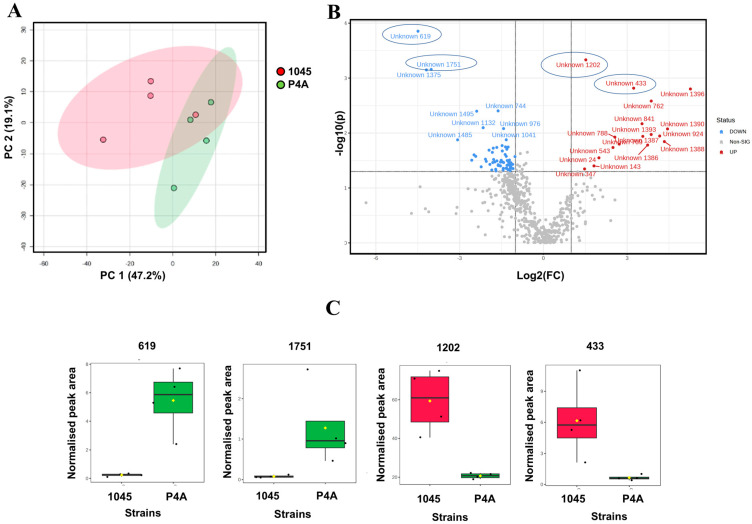
(**A**). PCA plot for lipids in P4A vs. 1045 nontreated strains. (**B**). Volcano Plot displaying the variations in lipids between the two strains (*p* < 0.05). (**C**). Selected compounds showing significant differences between the treatments 619 (mz = 849.5992), 1751 (mz = 826.6340), 1202 (mz = 339.3725), and 433 (mz = 846.6372) (*p* < 0.05).

In our analysis of *P. oleovorans* P4A, isolated from contaminated coating material, and 1045 reference strain, we investigated the lipid and metabolite profiles. We detected a total of 606 lipids and 1637 metabolites in both samples. Among these, 22 lipids and 40 metabolites were identified, while the remaining lipids and metabolites remained unknown. The principal component analysis (PCA) of the lipid profiles from the two groups of bacteria, showed notable differences (Figure 2A). To further investigate these differences, we constructed a Volcano plot (Figure 2B), showing the differences in the lipids between the strains. Detailed information on these compounds can be found in the Supplementary Material (Table S3).

At a nominal *p*-value, 66 compounds showed significant differences (*p* < 0.05); however, after applying a false discovery rate (FDR) correction with an FDR threshold of <0.1, only one unidentified lipid, unknown 619, exhibited a statistically significant difference between the two strains. 

**Figure 3:**
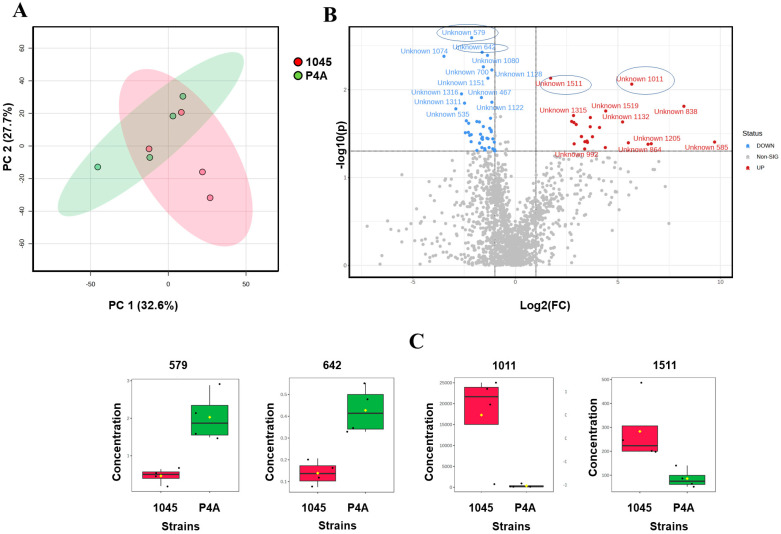
(**A**). PCA plot of polar metabolites in P4A and 1045 nontreated strains. (**B**). Volcano Plot illustrating the differences in polar metabolite profiles between *P. oleovorans* P4A and *P. oleovorans* 1045 strains, with a significance threshold of *p* < 0.05. (**C**). Selected Polar Metabolites, showing the significance (*p* < 0.05) of unknown compounds 579 (mz = 842.5731), 642 (mz = 703.4649), 1011 (mz = 730.4988), and 1511 (mz = 677.4415).

The polar metabolite profiles in the two bacterial groups also showed a distinct difference between the two *Pseudomonas* strains (Figure 3). Volcano Plot shows the variations in polar metabolites between *P. oleovorans* P4A and *P. oleovorans* 1045 (*p* < 0.05) (Figure 3B). Supplementary Material (Table S4) provides comprehensive details of these compounds. At a nominal *p*-value level, we found 70 compounds showing significant differences (*p* < 0.05), all of which were unidentified. However, after applying the false discovery rate (FDR) correction at a threshold of <0.1, none of the polar metabolites exhibited statistically significant differences.

### 3.2. Lipid and Metabolite Profiles in Biocide-Treated P. oleovorans 1045 Reference Strains

**Figure 4:**
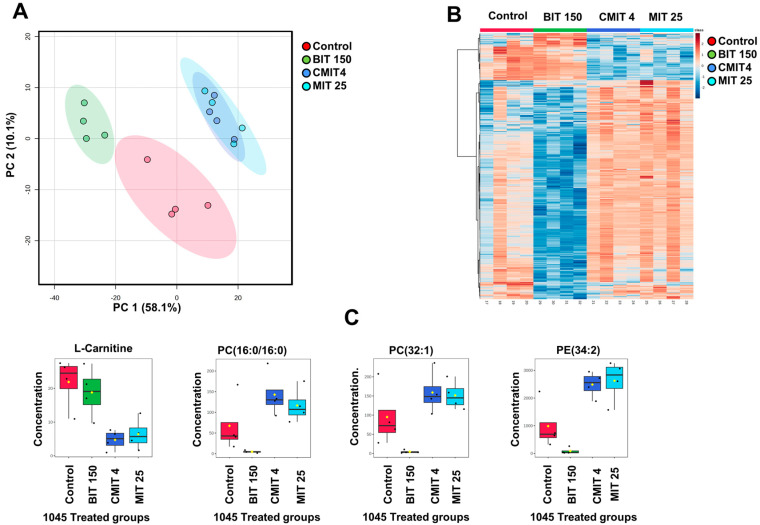
(**A**). PCA plots of Lipid Profiles in nontreated control and treated 1045 groups, highlighting the distinctions in lipidomic profiles between the different treatment groups and the non-treated group. (**B**). A Heatmap featuring 452 significantly altered lipids (ANOVA, *p* < 0.05) with samples organized based on exposure vs. untreated control groups of 1045. (**C**). Selected Lipids (*p* < 0.05): L-Carnitine, PC (16:0/16:0), PC (32:1) and PE (34:2).

First, we investigated the effects of biocide treatments on the metabolic profiles of the biocide-sensitive reference strain, *P. oleovorans* 1045, looking at both lipids and polar/semipolar metabolite profiles. The PCA of the lipid profiles in the bacterial groups subjected to biocide treatments compared to the untreated control (Figure 4A) revealed some similarities in the lipidomic profiles between the CMIT and MIT-exposed *P. oleovorans* 1045, contrasting with the BIT-exposed and untreated control groups. The BIT-exposure group exhibited a clear separation from the unexposed group in the PCA, as well as from the CMIT and MIT-exposed groups.

As presented by the heatmap (Figure 4B), ANOVA analysis (*p* < 0.05) showed significant differences at a nominal *p*-value level for 452 lipids out of a total of 606 across the *P. oleovorans* 1045 groups (control, CMIT-exposed, MIT-exposed, and BIT-exposed groups). Notably, distinctive patterns of lipids in the group exposed to BIT were observed compared to the other treatments. Interestingly, BIT induces opposite changes in the lipidome compared to the effects of the other two treatments. Highlighted in Figure 4C are four lipids that exhibited significant differences. Detailed information about these compounds is provided in Supplementary Table S5.

**Figure 5:**
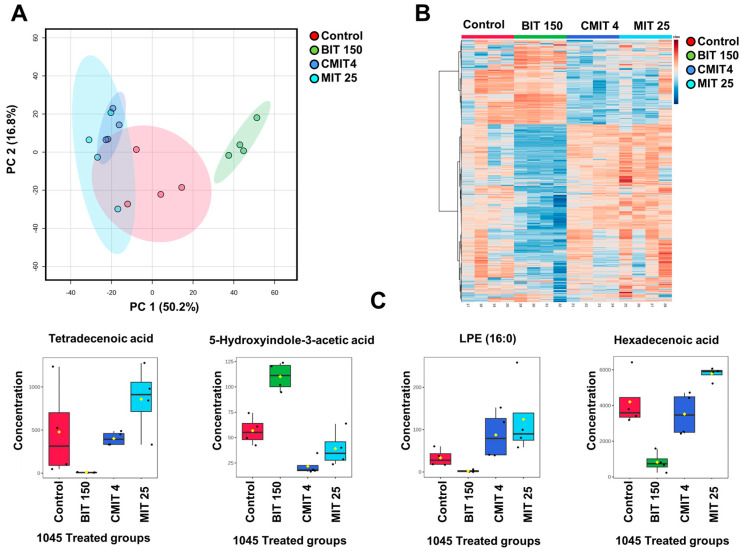
(**A**). PCA Plots of polar metabolite profiles in nontreated control and treated 1045 groups, showing the separation between the different treatment groups and the non-treated group. (**B**). A heatmap displaying significantly altered metabolites (ANOVA, *p* < 0.05) with samples organized based on exposure vs. nontreated groups of 1045. (**C**). Selected four metabolites (*p* < 0.05): tetradecenoic acid, 5-hydroxyindole-3-acetic acid, LPE (16:0), and hexadecenoic acid, showing statistically significant differences between the treatments.

The impact of biocide treatments on polar metabolic profiles (Figure 5A), also presented a clear separation between the different treatment groups. Again, the BIT-exposed group was clearly separated from the other groups in the PCA (Figure 5A). Similar to the lipidomic profiles, the separation can be explained by the opposite effect of the treatments in the polar metabolites, as demonstrated in a heatmap featuring 1000 metabolites selected based on ANOVA analysis (*p* < 0.05) (Figure 5B). This demonstrates that the BIT-exposed group displayed a markedly different polar metabolite pattern compared to the other groups. At a nominal *p*-value, the analysis revealed significant differences in 1161 out of a total of 1637 polar metabolites across the *P. oleovorans* 1045 groups, including the unexposed, CMIT-exposed, MIT-exposed, and BIT-exposed groups. Figure 5C highlights four known lipids that exhibited significant differences; detailed information about these compounds can be found in Supplementary Table S6. 

### 3.3. Lipids and Metabolites Profiles of Biocide-Treated P. oleovorans P4A Strain

**Figure 6:**
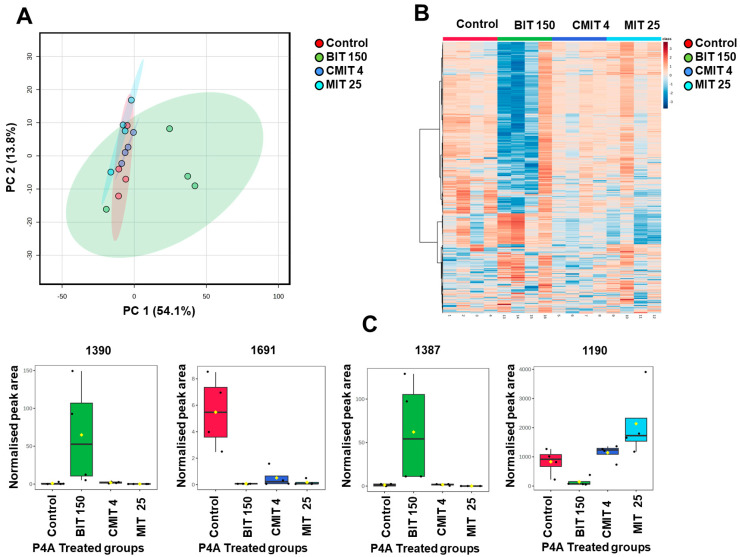
(**A**). PCA plots of lipid profiles in nontreated control and treated P4A groups, showing the differences in lipid profiles between the untreated control group and those exposed to MIT, BIT, and CMIT. (**B**). A Heatmap displaying 15 significantly altered lipids (ANOVA, *p* < 0.05) with samples organized based on exposure vs. untreated control groups of *P. oleovorans* P4A. (**C**). Selected four unknown lipid components (*p* < 0.05): 1390 (mms = 660.2531), 1691 (mz = 922.6007), 1387 (mz = 638.2711), and 1190 (mz = 313.3585). These unknown lipids exhibited statistically significant differences.

The PCA analysis of lipid profiles showed that the BIT-exposed group was mainly separated from the untreated control group, as well as groups exposed to MIT and CMIT (Figure 6A). 

To further investigate these differences, we generated a heatmap based on ANOVA (*p* < 0.05) (Figure 6B). As can be seen, the BIT-exposed group shows clearly different lipid patterns than the other groups. Overall, 15 lipids out of a total of 606 exhibited significant differences in *P. oleovorans* P4A across the treatment groups. Figure 6C shows four unidentified lipids that demonstrate significant differences. Detailed information about these compounds is available in Supplementary Table S7. The majority of these unknown compounds have a low retention time and small mz, indicating that they are small and polar compounds.

**Figure 7:**
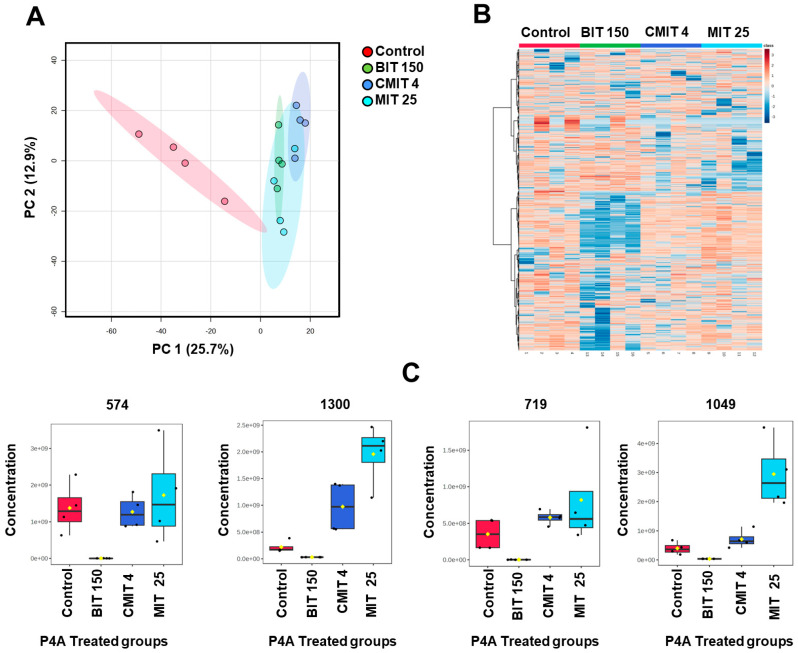
(**A**). PCA Plots of polar metabolite profiles in untreated control and treated P4A groups. (**B**). A heatmap displaying significantly altered metabolites (ANOVA, *p* < 0.05). (**C**). Selected unknown polar metabolite components (*p* < 0.05): 574 (mz = 642.3264), 1300 (mz = 551.26232), 719 (mz = 669.2799), and 1049 (mz = 539.3528). These unknown polar metabolites exhibited statistically significant differences.

The metabolic profiles across all P4A groups are shown in the PCA (Figure 7A). In the PCA plot, the untreated control group, as well as the CMIT and MIT groups, are clustered together, indicating similarities in their metabolic profiles while the BIT-exposed group is clearly separated, suggesting a stronger metabolic response to the treatment when compared to the other groups.

A heatmap constructed based on ANOVA, displaying 1000 metabolites with the most distinct patterns, (*p* < 0.05), further illustrates a more pronounced response to BIT exposure in comparison to other groups. We identified 332 unknown polar metabolites showing significant differences out of a total of 1637 metabolite components. Examples of the most significantly changed metabolites are presented in Figure 7C, while detailed information can be found in Supplementary Table S8.

### 3.4. Metabolic Pathway Analysis of BIT-Exposed P. oleovorans Strains

**Figure 8:**
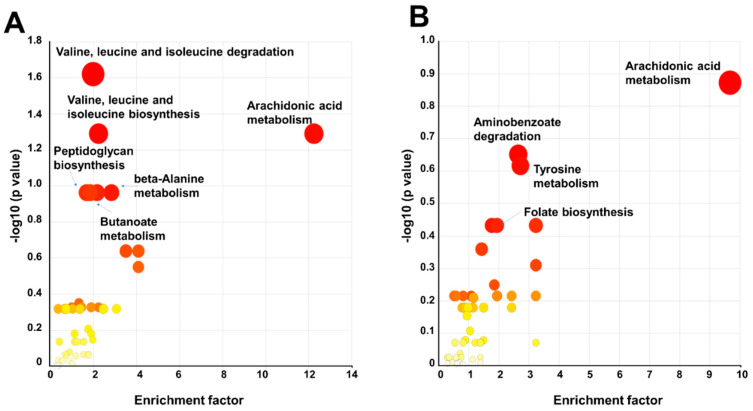
Metabolic pathway analysis of the (**A**) BIT-exposed 1045 strain and (**B**) the BIT-exposed P4A strain. Red color is showing significant changes.

We conducted metabolic pathway analysis to assess the impact of BIT treatment on the 1045 reference strain in comparison to the P4A strain, relative to their untreated control groups. In this analysis, we utilized the data of polar and semipolar metabolites due to limitations of lipidomic pathway mapping for lipids. The Mummichog Pathway Analysis Plot revealed significant enrichment in multiple metabolic pathways, namely in valine, leucine, isoleucine degradation, and valine, leucine, and isoleucine biosynthesis, arachidonic acid metabolism, Aminoacyl–tRNA biosynthesis, peptidoglycan biosynthesis, butanoate metabolism, pantothenate and CoA biosynthesis, ubiquinone and another terpenoid-quinone biosynthesis, and porphyrin and chlorophyll metabolism (Figure 8A,B, Table 2 and Table 3, Supplementary Table S9). We further checked the identity of those metabolites identified by the pathway analysis to be significant and could confirm several additional metabolites, in addition to those that we had in our target list. 

These findings indicate that the BIT treatment had clear effects on these metabolic pathways in the 1045 strain, suggesting that biocide treatment induces changes in amino acid and fatty acid metabolism. The results of the pathway analysis are presented in Table 1 and Table 2. We also tested the pathway analysis for the lipidomics data; however, this approach proved to be not feasible, probably due to the challenges in mapping lipids into specific pathways when the exact fatty acid composition is not known. Several of the putatively identified lipids that showed response to the BIT were oxidized phospholipids, and several of them contained arachidonic acid in their structure. Due to a lack of reference spectra, full identification was not possible. 

BIT treatment, and a large number of oxidized phospholipids, several of them having arachidonic acid in their structure, were found. However, due to the lack of reference standards and experimental reference spectrum, we could not verify the identifications.

The outcomes of our KEGG metabolomic pathway analysis on the P4A strain exposed to 150 ppm of BIT are presented in Figure 8B and Table 2. The analysis revealed notable effects on specific metabolic pathways, including arachidonic acid metabolism, tyrosine metabolism, aminobenzoate degradation, folate biosynthesis, ubiquinone and other terpenoid-quinone biosynthesis, and Aminoacyl–tRNA biosynthesis due to the BIT treatment. Additionally, dysregulation of arachidonic acid metabolism was observed, mirroring the changes observed in the 1045 strain, although the difference did not reach statistical significance.

## 4. Discussion

In this study, our initial focus was on investigating potential disparities in the metabolic profiles of two *Pseudomonas oleovorans* strains. Subsequently, we investigated how exposure to biocides, using similar conditions that industry applies in their microbiological tests, impacts the metabolome of these strains, with a specific emphasis on lipid, polar, and semipolar metabolites. Lipids play a crucial role in cell structure and signaling, while polar and semipolar metabolites serve as key intermediates in various metabolic pathways. By focusing on these metabolite classes, we aimed to gain specific insights into the adaptive responses of *Pseudomonas strains* to biocide exposure. While our metabolic coverage does include all main molecular lipids and key metabolic pathways, there are some limitations in the coverage of metabolites, which are either present at very low concentrations or which would require specific sample extraction methods due to, e.g., instability. 

The present study demonstrated a significant influence of three biocides on the metabolic profiles of an industrial biocide-resistant strain of *P. oleovorans* P4A and a biocide-sensitive reference strain *P. oleovorans* 1045. The biocide concentrations were sub-MIC to prevent the inhibition of bacterial growth but permitted the analysis of the effects of the biocides on the microbes. While the conditions chosen were similar to those used by coating companies in their microbiological tests, they do not fully mimic the real-life conditions related to possible fluctuations in temperature, substrate and nutrient exposure, pH variations, and the presence of other microorganisms that can influence bacterial metabolism. The two strains had similar overall lipidomic and metabolic profiles, with none of the differences reaching statistical significance. This suggests that the reference strain (1045) could be used as a model when investigating the impact of various treatments on the metabolic level. 

Our findings demonstrated that biocides used in the study had different effects on bacteria metabolism. This aligns with previous research indicating that different biocidal agents operate through distinct mechanisms of action, resulting in varying capacities to hinder or eradicate microbial communities. These mechanisms include, but are not limited to, the degradation of cellular membranes, disruption of intracellular proteins, and the inactivation of specific enzymatic pathways within the microorganism [1]. Similar metabolic changes have been reported in other *Pseudomonas* strains after exposure to antibiotics. Particularly, changes in phospholipids are a specific feature of stress response in *Pseudomonas*, which adapts to environmental stress by converting *cis* unsaturated membrane fatty acids to their *trans* configuration, rapidly altering phospholipids [39]. *Pseudomonas* also uses vesiculation to defend against chemical stress, increasing its surface hydrophobicity and enhancing biofilm formation. *P. aeruginosa*, a well-studied strain, shows metabolic changes in response to antibiotics like Polymyxin B, which alters lipid profiles, including decreases in free fatty acids and time- and dose-dependent changes in phospholipids [40]. In antibiotic-resistant strains, enhanced fatty acid biosynthesis is a key feature of CIP-resistant *P. aeruginosa* [39,41].

Overall, the metabolic changes observed after biocide treatment were more pronounced in the 1045 strain than in the P4A strain (1613 lipids and metabolites showing significant changes in the 1045 strain, 347 in the P4A strain), with both lipids and more polar metabolites showing differences. 

At the level of individual metabolites, several changes related to adaptive and/or stress responses, such as oxidative stress were observed. The critical involvement of L-carnitine in lipid metabolism and its significance as a fatty acid transporter are well-documented in the literature. L-carnitine plays a central role in facilitating the transport of fatty acids, thus contributing to energy production in aerobic organisms [42,43]. This process is particularly important during periods of heightened metabolic demands or stress conditions, ensuring the balance of lipid oxidation within cells. In the context of *P. oleovorans*, exposure to biocides may induce a stress response that perturbs normal metabolic pathways, including those regulated by L-carnitine. Biocides are known to disrupt bacterial cellular processes and induce stress, potentially leading to alterations in metabolic pathways [44]. The observed decrease in L-carnitine levels in *P. oleovorans* post-biocide exposure suggests interference with lipid metabolism, which could adversely affect energy production and bacterial survival. The findings align with previous studies highlighting the impact of biocides on microbial metabolism. Biocides can disrupt cellular homeostasis and metabolic pathways, including those associated with lipid metabolism [45]. 

It is also involved in the elimination of cellular metabolic byproducts. L-carnitine is involved in regulating stress responses, energy metabolism, and protein folding, irrespective of whether bacteria inhabit aerobic or anaerobic environments [46]. An important function of L-carnitine is its ability to mitigate osmotic stress in bacteria by acting as an osmolyte, thus helping maintain cellular fluid levels and preventing cellular damage [47]. 

The significant alteration we observed on several phospholipids could imply modification of bacterial membranes, which can impact the structural attributes, morphology, and content of cell membranes [48]. Phosphatidylcholine (PC) lipids are associated with bacterial survival and adaptation to stress environments [49]. *P. oleovorans* 1045 subjected to CMIT and MIT exposure exhibited elevated concentrations of PCs, while exposure to BIT displayed a notable reduction in PC levels. *P. oleovorans* traditionally incorporate phospholipids other than phosphatidylcholine (PC) into their membrane structure as an adaptive mechanism to fine-tune their membrane composition in order to meet specific environmental and metabolic demands [49]. The capacity of *Pseudomonas* species to assimilate PC into their membranes could confer advantages regarding heightened toxicity, since PC-rich membranes may enhance stability and fluidity, potentially providing a competitive edge to these bacteria [49]. The presence of PC in bacterial membranes is rare and considered a distinctive feature among bacteria. Alterations in PC levels may also have consequences on membrane permeability, potentially affecting the processes of biocide uptake and efflux [50]. Thus, alteration in PC concentrations is related to cellular membrane characteristics, and the fundamental mechanisms underlying biocide resistance. The changes observed in phosphatidylethanolamines (PEs), such as PE (34:2), after biocide treatment can also be linked with changes in bacterial membranes. The PE (34:2) levels in *P. oleovorans* 1045 exposed to BIT resulted in a reduction, whereas exposure to CMIT and MIT induced increased levels of this lipid. PE (34:2) is a component found within the bacterial cell membrane, contributing to its structural integrity, selective permeability, and protein anchoring capabilities. The responsive nature of PE (34:2) to biocidal exposure suggests its involvement in the bacterium’s adaptive mechanisms in the face of environmental stressors [51]. PEs function as a discerning permeability barrier in *Pseudomonas* species and selectively facilitate the entrance of vital nutrients and metabolites into the cell while impeding the passage of deleterious substances [52]. PE (34:2) potentially contributes to the controlled translocation of molecules across the membrane and serves as an anchor for membrane proteins associated with nutrient transport, signal transduction, and enzymatic catalysis [53].

We also show dysregulation of a monounsaturated free fatty acid, namely, tetradecanoyl acid, which showed elevated concentrations in *P. oleovorans* P4A exposed to CMIT and MIT, while reduced levels in BIT exposure. Tetradecenoic acid influences membrane fluidity and functionality [28]. Previous studies have suggested that the presence and metabolic utilization of tetradecenoic acid are contingent upon growth conditions and adaptive responses [54]. The composition of free fatty acids within bacterial membranes can vary significantly, influenced by the growth medium, temperature, and other environmental parameters [55]. 

Other metabolites than those belonging to lipids also showed differences. 5-Hydroxyindole-3-acetic acid (5-HIAA) was downregulated in the P4A strain exposed to CMIT and MIT, whereas significantly higher concentrations were detected after BIT exposure. The biosynthesis of 5-HIAA in *Pseudomonas* species primarily occurs through the conversion of tryptophan to indole-3-acetic acid (IAA), facilitated by the enzyme tryptophan decarboxylase (TrpDC) [56]. While the precise functions of 5-HIAA in *Pseudomonas* are not fully understood, evidence exists that it is involved in responses to environmental stresses, such as oxidative stress, and exposure to toxic compounds. It is plausible that 5-HIAA functions as a signaling molecule in stress adaptation mechanisms [57]. 

On the metabolic pathway level, several key metabolic pathways showed dysregulation after treatment with BIT. It should be noted that, while the metabolic pathway analysis methodology links metabolites to biological pathways, the functional outcomes are limited by the pathway libraries and the analytical method [58]. Among the pathways identified after BIT exposure, two pathways significantly altered in both *P. oleovorans* strains were arachidonic acid metabolism and ubiquinone and other terpenoid-quinone biosynthesis. Arachidonic acid (AA) is a polyunsaturated fatty acid primarily found in eukaryotic cell membranes and serves as a precursor for bioactive lipid mediators, including prostaglandins and leukotrienes, which regulate inflammatory and immune responses. While AA did not show any significant differences between the treatments, several oxylipins, such as 9,10-diHOME, showed significantly increased levels especially in the P4A strain (FC = 4.5, *p* = 0.001). In addition, the lipidomics data showed that several putatively identified AA fatty acyl containing oxidized phospholipids were significantly altered after BIT treatment. In *Pseudomonas* species, the AA metabolism involves transport systems for its uptake from the environment and enzymatic processes, such as β-oxidation, to break it down into acyl-CoA molecules [59]. While there is currently a limited amount of information on the role of AA in *Pseudomonas oleovorans*, there are some studies on *Pseudomonas aeruginosa,* which shares genetic similarities with *P. oleovorans*. Specific enzymes (such as CYP168A1) that act as fatty acid hydroxylases and metabolize arachidonic acid into oxylipins have been identified in *Pseudomonas aeruginosa*. The activity of this enzyme is inhibited by the antifungal agent ketoconazole [58]. This inhibition response could be linked to the changes in the arachidonic acid pathways observed in our study. It could be also linked with alteration in phospholipid fatty acids composition, which has been reported in the response to different environmental cues for *Pseudomonas putida* [60], as well as specific enzymes involved in the oxidative stress response among different strains of *P. putida* [61]. Of the other pathways affected, the ubiquinone and other terpenoid-quinone biosynthesis pathways could also potentially be associated with a stress response to the biocide treatment. Ubiquinone, a critical terpenoid quinone in *P. oleovorans*, acts as an electron carrier in cellular respiration and serves in antioxidant defence and apoptosis [62]. Its biosynthesis from chorismite involves several enzymatic steps, similar to the pathway for menaquinone, essential for the bacterium’s survival [63]. 

Several pathways that are linked with amino acid metabolism were also dysregulated by the BIT treatment. Aminoacyl–tRNA biosynthesis, facilitated by Aminoacyl–tRNA synthetases (aaRSs), ensures accurate protein translation in *P. oleovorans.* Both strains showed alterations to the Aminoacyl–tRNA biosynthesis pathway. Disrupted amino acid metabolism can potentially have implications for the bacteria’s functioning, particularly in relation to protein synthesis and metabolic activities. In bacteria, when there is a disturbance in the way amino acids (the building blocks of proteins) are managed, it can be linked to issues with multiple essential amino acids, specifically valine, leucine, and isoleucine. These amino acids are crucial for making proteins and carrying out important metabolic functions in bacteria [64]. Three metabolites in this pathway were identified/putatively identified, of those two were downregulated and leucine/isoleucine was upregulated. The degradation of these amino acids was significant in the 1045 strain, while it was altered, although not statistically significant (*p* = 0.07) in the P4A strain. Valine, leucine, and isoleucine undergo catabolism in a series of enzymatic reactions leading to the production of key intermediates such as isobutyl-CoAisobutyl-CoA, isovaleryl-CoA, and β-methylcrotonyl-CoA. These intermediates are converted into acetyl-CoA, which enters the Citric acid cycle for energy generation. Tyrosine metabolism in P4A after BIT exposure was also altered. The tyrosine metabolism pathway in *P. oleovorans* provides amino acids and other molecules necessary for growth and development. In addition to the main tyrosine metabolism pathway, *Pseudomonas* species also have several other enzymes that can metabolize tyrosine. For example, some *Pseudomonas* species have enzymes that can convert tyrosine to catechol, which is a precursor to a variety of siderophores [65]. 

Several pathways that suggest responses related to bacterial growth and survival were also observed. Dysregulation was observed in the aminobenzoate degradation pathway, which in *P. oleovorans* is an important pathway for the degradation of aromatic compounds and contributes to the bacterium’s ability to grow in environments that are contaminated with aromatic compounds [66,67]. This pathway was impacted in the P4A strain by the BIT treatment is linked with catechol metabolism by cleavage of aminobenzoate and 2-amino-3-ketobutyrate. This pathway is also linked with amino acid metabolism as *P. oleovorans* has several enzymes that degrade catechol to further metabolic products such as glutamate which is a central metabolite in energy production, protein synthesis, and amino acid metabolism [68,69]. Only one of the three metabolites in this pathway, identified in pathway analysis could be putatively identified, showing upregulation after exposure. Vitamin B metabolism, which is vital in the growth and survival of organisms [70], was also affected in both two strains, with the 1045 strain showing dysregulation of pantothenate metabolism and the P4A strain in folate metabolism due to BIT treatment. Both pathways are also connected to multiple amino acid pathways. In both pathways, two metabolites identified by the pathway analysis were putatively identified, with pantetheine showing downregulation and its direct derivative pantothenic acid upregulation. Particularly, the changes in the folate pathway can also be important in the survival of bacteria in diverse environmental conditions, as folate is central to one-carbon metabolism, facilitating the assimilation of carbon sources and nitrogen metabolism [51,71]. Folate (vitamin B9) is a crucial cofactor essential for various cellular processes, and while some bacteria cannot synthesize folate de novo, *P. oleovorans* possesses the genetic machinery for de novo folate biosynthesis [72]. 

The increased peptidoglycan biosynthesis in P4A may be a mechanism of resistance to biocides as peptidoglycan may contribute to the protection of the cell from the biocides, and this may make the cell more resistant. Peptidoglycan biosynthesis was affected in both *P. oleovorans* strains after biocide treatment, but the response level was different. This difference may be because P4A is resistant to biocides, while 1045 is not. Peptidoglycan is a complex polymer that is essential for the growth and survival of bacteria. It protects the external environment and maintains the shape of the cell [73]. While we could not verify the identity of the metabolites identified by the pathway analysis in this pathway, due to a lack of reference spectra, all four compounds in this pathway showed significant upregulation (FC 3–10).

The butanoate metabolic pathway, which is linked with both energy metabolism and amino acid and fatty acid metabolism, and thus, also to growth, was affected in both strains exposed to BIT exposure but in different levels of response. Of identified metabolites, 4-aminobutanoate was upregulated in both strains. Butanoate metabolism in *P. oleovorans* is the conversion of butanoate, a four-carbon fatty acid, into either energy or other useful cellular metabolites. Butanoate can be broken down to produce acetyl-CoA, which is an intermediate in the citric acid cycle and part of the energy production in aerobic organisms [74]. *P. oleovorans* can also use butanoate to produce other cellular metabolites, such as amino acids, fatty acids, and polyhydroxyalkanoates. Butanoate metabolism is part of the metabolic versatility of *P. oleovorans* by allowing the bacteria to grow on a variety of different carbon sources, including butanoate, other fatty acids, and hydrocarbons.

In summary, our study showed heterogeneous effects of biocides on the metabolic profiles of the two bacterial strains, with the biocide-sensitive strain (1045) exhibiting more pronounced alterations compared to the biocide-resistant strain (P4A). The observed changes indicate alteration in cellular membranes and bacterial metabolism, thereby potentially influencing bacterial growth and survival. Overall, the observed changes in lipids and other metabolites implied potential adaptive responses to biocides. The main finding of our study was that the three biocide mixtures triggered different types of metabolic changes, with BIT showing the strongest metabolic response. This could explain why it has been successfully applied in long-term preservation while the two other biocides are used for short-term preservation, with their impact diminishing rapidly. Here, it should also be noted that the exposure concentration for BIT was higher than for CIT and MIT. In addition, BIT is clearly more hydrophobic than the other two biocides, and that could potentially contribute to its strong impact on particularly lipid metabolism. Our study also demonstrates the possibilities of using metabolomics as a tool for investigating the biological responses of bacteria after biocide treatment, which could be utilized in the targeted development of more efficient biocide treatments, with benefits both for industry and potentially, also for the environment. Future research should prioritize identifying synergistic biocide combinations against biocide-resistant microorganisms, elucidating their mechanisms of action, formulating new delivery systems, and assessing their safety and efficacy. 

## Figures and Tables

**Table 1:** LC–MS conditions for the two methods used in the study.

Conditions	Polar/Semipolar Compounds	Lipidomics
Injection volume	10 µL	1 µL
Column	C18 precolumn (Waters Corporation, Wexford, Ireland) and an inline filter, pore size 0.2 µm (Waters Corporation, Wexford, Ireland). + ACQUITY UPLC^®^ BEH C18 column (2.1 mm × 100 mm, particle size 1.7 µm) by Waters (Milford, MA, USA)	C18 precolumn (Waters Corporation, Wexford, Ireland) and an inline filter, pore size 0.2 µm (Waters Corporation, Wexford, Ireland). + ACQUITY UPLC^®^ BEH C18 column (2.1 mm × 100 mm, particle size 1.7 µm) by Waters (Milford, MA, USA)
Mobile phases	A H_2_O:MeOH (*v*/*v* 70:30) with 2 mM ammonium acetate B MeOH with containing 2 mM ammonium acetate	A 10 mM ammonium acetate and 0.1% Formic Acid in H_2_O B Acetonitrile:Isopropanol (*v*/*v* 1:1) with 0.1% Formic Acid and 10 mM ammonium acetate
Gradient	0–1.5 min: B was increased from 5% to 30% 1.5–4.5 min, B increased to 70%; 4.5–7.5 min, B increased to 100% and held for 5.5 min. A post-time of 6 min	0–2 min, B was increased from 35% to 80% 2–7 min, B increased to 100% 7–14 min, B was held at 100%. A post-time of 7 min
Flow rate	0.4 mL min^−1^	0.4 mL min^−1^
MS conditions	Dual ESI ionization source with capillary voltage 4.5 kV, nozzle voltage 1500 V, N_2_ pressure in the nebulized was 21 psi and the N_2_ flow rate and temperature as sheath gas was 11 L min^−1^ and 379 °C, respectively. The drying gas flow was set to 10 L min^−1^ and the temperature to 150 °C. m/z range 100–1700 in negative ion mode.	Dual ESI ionization source with capillary voltage 3.64 kV, nozzle voltage 1500 V, N_2_ pressure in the nebulized was 21 psi and the N_2_ flow rate and temperature as sheath gas was 11 L min^−1^ and 379 °C, respectively. The drying gas flow was set to 10 L min^−1^ and temperature to 193 °C. m/z range 100–1700 in positive ion mode

**Table 2:** KEGG pathways were identified as the output of the mummichog analysis in this analysis of 1045 strain untreated control samples vs. 1045 strain 150 ppm BIT-exposed sample.

Pathway Analysis for 1045	Pathway Total	Hits. Total	Hits. Sig	P. Fisher	P. EASE	P. Gamma
Valine, leucine, and isoleucine degradation	31	5	5	0.0239	0.1531	0.0052
Valine, leucine, and isoleucine biosynthesis	22	4	4	0.0512	0.2724	0.0071
Arachidonic acid metabolism	4	4	4	0.0512	0.2724	0.0071
beta-Alanine metabolism	13	3	3	0.1088	0.4613	0.0121
Peptidoglycan biosynthesis	17	3	3	0.1088	0.4613	0.0121
Butanoate metabolism	22	3	3	0.1088	0.4613	0.0121
Pantothenate and CoA biosynthesis	20	3	3	0.1088	0.4613	0.0121
Ubiquinone and other terpenoid-quinone biosynthesis	9	4	3	0.2816	0.6503	0.0220
Porphyrin and chlorophyll metabolism	56	11	6	0.4463	0.6711	0.0237

**Table 3:** KEGG pathway was identified as the output of the mummichog analysis in this analysis of P4A strain untreated control samples vs. P4A 150 ppm Bit-exposed sample groups.

Pathway Analysis for P4A	Pathway Total	Hits. Total	Hits. Sig	P. Fisher	P. EASE	P. Gamma
Arachidonic acid metabolism	4	4	4	0.1341	0.4786	0.0158
Tyrosine metabolism	25	9	7	0.2417	0.4876	0.0162
Aminobenzoate degradation	11	3	3	0.2230	0.6527	0.0261
Folate biosynthesis	34	7	5	0.4371	0.7191	0.0323
Ubiquinone and other terpenoid-quinone biosynthesis	9	4	3	0.4898	0.8267	0.0485
Aminoacyl–tRNA biosynthesis	21	6	4	0.5632	0.8289	0.0489
Porphyrin and chlorophyll metabolism	56	11	6	0.7807	0.9118	0.0738
Valine, leucine, and isoleucine degradation	31	5	3	0.7004	0.9184	0.0769
Biotin metabolism	6	2	2	0.3693	0.8436	0.0521
Peptidoglycan biosynthesis	17	3	2	0.6619	0.9391	0.0885
Butanoate metabolism	22	3	2	0.6619	0.9391	0.0885
Pantothenate and CoA biosynthesis	20	3	2	0.6619	0.9391	0.0885
Valine, leucine, and isoleucine biosynthesis	22	4	2	0.8340	0.9765	0.1271

## Data Availability

The original contributions presented in the study are included in the article/Supplementary Material, further inquiries can be directed to the corresponding author.

## Supplementary Materials

The following supporting information can be downloaded at: https://www.mdpi.com/article/10.3390/metabo14060326/s1, Table S1: Internal standards added to the extraction solution for polar metabolite and lipidomic analysis; Table S2: Compounds used in calibration curves; Table S3: Lipid showing significant differences at nominal *p* value level in nontreated *P. oleovorans*. 1045 vs. P4A strains; Table S4: Polar metabolites showing significant differences at nominal *p* value level between *P. oleovorans*. 1045 vs. P4A strains; Table S5: ANOVA analysis of lipids in biocide treated *P. oleovorans* 1045 strain; Table S6: ANOVA analysis of polar metabolites biocide treated *P. oleovorans* 1045 strain; Table S7: ANOVA analysis of lipids in biocide treated *P. oleovorans* P4A strain; Table S8: ANOVA analysis of polar metabolites in biocide treated *P. oleovorans* P4A strain; Table S9: Identification of Metabolites Associated with Specific Metabolic Pathways. The compounds identification is done via an automatic annotation based on the possible adducts proposed by MetaboAnalyst. Compounds marked in bold have been identified manually based on their mass spectrum and by comparison with reference library (hmbd.ca) or standards, if available.
