# Supplementary material for: Metabolic Changes in Pseudomonas oleovorans Isolated from Contaminated Construction Material Exposed to Varied Biocide Treatments
Source: Metabolites. 2024 Jun 10;14(6):326. doi: 10.3390/metabo14060326 (PMC11205842; doi:10.3390/metabo14060326)
Supplement: Supplementary file 1 [file metabolites-14-00326-s001.zip › metabolites-2990168-supplementary.pdf]

Supplementary material

Supplementary Table S1: Internal standards added to the extraction solution for polar metabolite and lipidomic analysis.

Compound	C µg/mL
**POLAR METABOLITES**	
Hexanoic acid-d3 (MeOH) *	10
Heptadecanoic acid	10
Betaine-d11	5
Tryptophan (indole)-d5	30
Valine-d8	20
Indole-d6	23.33
Benzoic acid-d5	13.33
Succinic acid-d4	20
Taurocholic acid (TCA)-d4	1
Lithocholic acid (LCA)-d4	1
Glycolithocholic acid (GLCA)-d4	1
Glycoursodeoxycholic acid (GUDCA)-d4	1
Deoxycholic acid (DCA)-d4	1
Glycochenodeoxycholic acid (GDCA)-d4	1
Glycocholic acid (GCA)-d4	1
Cholic acid (CA)-d4	1
Ursodeoxycholic acid (UDCA)-d4	1
M8PFOA-13C8	0.2
MPFNA-13C5	0.2
M7PFUnDA-13C7	0.2
M3PFHxS-13C3	0.2
M8PFOS-13C8	0.2
**LIPIDOMICS**	
Cer (d18:1/17:0)	1
LPC (17:0)	1
PC (16:0/d31/18:1)	1
PC (17:0/17:0)	1
PE (17:0/17:0)	1
SM(d18:1/17:0)	1
TG (17:0/17:0/17:0)	1

Supplementary Table S2: Compounds used in calibration curves

LIPIDOMICS	POLAR METABOLITES
CE (18:0)	7-oxo-hyocholic acid (7-oxo-HCA)
CE (18:2)	7-oxo-deoxycholic acid (7-oxo-DCA)
Cer(d18:0/18:1(9Z))	alpha-Muricholic acid(αMCA)
DG (18:0/18:2)	beta-Muricholic acid(βMCA)
LPC (16:0)	omega-uricholic acid(ωMCA)
LPC (18:0)	Cholic acid (CA)
LPC (18:1)	Deoxycholic acid (DCA)
LPE (18:1)	Glycocholic acid (GCA)
PC (16:0e/18:1(9Z)	Glycochenodeoxycholic acid (GCDCA)
PC (18:0p/18:1(9Z)	Glycodeoxycholic acid (GDCA)
PC (18:0p/22:6)	Glycolitocholic acid (GLCA)
PE (16:0/18:1)	Glycoursodeoxycholic acid (GUDCA)
PE (16:0/18:1(9Z)	Hyodeoxycholic acid (HDCA)
TG (16:0/16:0/16:0)	Litocholic acid (LCA)
TG (18:0/18:0/18:0)	Tauro-alpha-muricholic acid (TαMCA)
	Tauro-beta-muricholic acid (TβMCA)
	Tauro-omega-muricholic acid (TωMCA)
	Taurocholic acid (TCA)
	Taurochenodeoxycholic acid (TCDCA)
	Taurodeoxycjolic acid (TDCA)
	Taurohyodeoxycholic acid (THDCA)
	Taurolitocholic acid (TLCA)
	Tauroursodeoxycholic acid (TUDCA)
	Alanine
	Asparagine
	Aspartic acid
	Glutamic acid
	Glutamine
	Glycine
	Isoleucine
	Leucine
	Methionine
	Ornithine
	Phenylalanine
	Proline
	Serine
	Threonine
	Valine
	2-hydroxybutyric acid
	3-hydroxybutyric acid Arachidonic acid
	Citric acid
	Fumaric acid
	Isocitric acid
	Lactic acid
	Linoleic acid
	Malic acid
	Octanoic acid
	Oleic acid
	Palmitic acid
	Stearic acid
	Succinic acid
	Cholesterol
	Fructose
	Glycerol-3-phosphate

Supplementary Table S3: Lipid showing significant differences at nominal p value level in nontreated *P. oleovorans*. 1045 vs. P4A strains.

Lipid Name	m/z	Retention time	t.stat	p.value	FDR
Unknown 619	849.5992	6.61	-8.55	0.0001	0.085
Unknown 1202	339.3725	3.57	6.88	0.0005	0.1075
Unknown 1751	826.6340	6.85	-6.38	0.0007	0.1075
Unknown 1375	165.0451	0.81	-6.36	0.0007	0.1075
Unknown 433	846.6372	7.53	5.49	0.0015	0.1594
Unknown 1396	583.7646	0.92	5.46	0.0016	0.1594
Unknown 762	652.4041	0.8	4.93	0.0026	0.2267
Unknown 744	197.0700	0.84	-4.54	0.004	0.2698
Unknown 1495	585.2466	3.49	-4.52	0.004	0.2698
Unknown 841	134.1168	0.73	4.04	0.0068	0.3133
Unknown 1132	563.2647	3.5	-3.9	0.008	0.3133
Unknown 976	509.3612	7.73	-3.87	0.0083	0.3133
Unknown 1390	660.2531	0.86	3.85	0.0084	0.3133
Unknown 1393	558.3275	0.9	3.66	0.0106	0.3133
Unknown 924	598.3310	0.86	3.6	0.0113	0.3133
Unknown 1387	638.2711	0.85	3.59	0.0115	0.3133
Unknown 788	137.0676	0.73	3.56	0.0119	0.3133
Unknown 1041	671.5026	7.72	-3.47	0.0133	0.3133
Unknown 1485	355.4040	3.4	-3.47	0.0133	0.3133
Unknown 1388	620.3135	0.86	3.41	0.0143	0.3133
Unknown 709	723.5232	11.15	3.33	0.0159	0.3133
Unknown 1386	576.3494	0.85	3.29	0.0166	0.3133
Unknown 1648	930.5659	6.15	-3.28	0.0169	0.3133
Unknown 835	659.5049	7.72	-3.23	0.0179	0.3133
Unknown 607	387.2519	7.71	-3.23	0.018	0.3133
Unknown 907	772.5847	7.29	-3.21	0.0183	0.3133
Unknown 543	723.5235	7.51	3.21	0.0183	0.3133
Unknown 1068	1002.7726	7.86	-3.19	0.0188	0.3133
Unknown 1073	535.3775	7.72	-3.15	0.0197	0.3133
Unknown 1191	122.0704	0.74	-3.12	0.0205	0.3133
Unknown 403	725.5557	6.33	-3.1	0.0212	0.3133
Unknown 961	921.7436	8.07	-3.05	0.0224	0.3133
Unknown 1622	903.5348	6.04	-3.05	0.0225	0.3133
Unknown 1798	839.6667	7.71	-3.01	0.0236	0.3133
Unknown 1043	1004.7875	8.15	-2.98	0.0246	0.3133
Unknown 1501	355.4037	3.67	-2.97	0.025	0.3133
Unknown 1156	713.5501	7.71	-2.92	0.0266	0.3133
Unknown 808	831.5900	6.84	-2.92	0.0266	0.3133
Unknown 1685	896.4600	6.35	-2.91	0.0268	0.3133
Unknown 24	723.5236	9.39	2.87	0.0283	0.3133
Unknown 963	828.6502	6.92	-2.83	0.0299	0.3133
Unknown 1262	769.4982	5.94	-2.82	0.0303	0.3133
Unknown 1313	795.6279	7.72	-2.8	0.0311	0.3133
Unknown 1757	718.5406	6.9	-2.8	0.0313	0.3133

Unknown 949	1035.7593	8.09	-2.8	0.0313	0.3133
Unknown 245	763.6016	7.68	-2.79	0.0317	0.3133
Unknown 1295	764.5426	5.9	-2.78	0.0322	0.3133
Unknown 1744	750.5716	6.79	-2.76	0.0328	0.3133
Unknown 1145	523.3769	7.71	-2.75	0.0334	0.3133
Unknown 1193	710.4724	5.89	-2.75	0.0335	0.3133
Unknown 1807	1028.7889	7.79	-2.74	0.0336	0.3133
Unknown 1005	734.5330	6.1	-2.74	0.0336	0.3133
Unknown 1160	1030.8035	8.08	-2.74	0.0338	0.3133
Unknown 1157	575.5021	6.35	-2.72	0.0344	0.3133
Unknown 1756	1167.0218	6.89	-2.72	0.0344	0.3133
Unknown 1801	812.6550	7.72	-2.72	0.0348	0.3133
Unknown 1243	734.4699	6.32	-2.7	0.0357	0.3133
Unknown 799	766.5352	6.89	-2.7	0.0358	0.3133
Unknown 1299	712.4882	6.31	-2.67	0.0372	0.3133
Unknown 303	265.1114	0.77	-2.67	0.0373	0.3133
Unknown 1173	603.4399	7.72	-2.66	0.0373	0.3133
Unknown 1251	575.5017	5.91	-2.65	0.0378	0.3133
Unknown 1106	710.4726	5.94	-2.65	0.038	0.3133
Unknown 1728	856.5041	6.69	-2.65	0.0381	0.3133
Unknown 1260	797.5289	6.27	-2.64	0.0385	0.3133
Unknown 1085	962.7830	8.34	-2.64	0.0387	0.3133

Supplementary Table S4: Polar metabolites showing significant differences at nominal p value level between *P. oleovorans*. 1045 vs. P4A strains

Polar Mutabolies Name	m/z	Retention time	t.stat	p.value	FDR
Unknown 579	842.5731	8.7185	-4.96	0.0026	0.7056
Unknown 642	703.4649	8.2555	-4.59	0.0037	0.7056
Unknown 1080	730.4988	8.2554	-4.51	0.0041	0.7056
Unknown 1074	677.4415	8.1787	-4.49	0.0042	0.7056
Unknown 700	761.4926	7.7642	-4.23	0.0055	0.7056
Unknown 1128	763.5148	7.9645	-4.16	0.006	0.7056
Unknown 1511	344.2187	1.5328	3.97	0.0074	0.7056
Unknown 1151	702.4727	8.3862	-3.97	0.0074	0.7056
Unknown 1011	570.3281	2.8473	3.83	0.0086	0.7056
Unknown 1316	842.599	8.4465	-3.61	0.0112	0.7056
Unknown 467	687.4139	6.7169	-3.54	0.0123	0.7056
Unknown 1122	438.5993	0.5940	-3.44	0.0139	0.7056
Unknown 1311	247.0995	3.3976	-3.42	0.0142	0.7056
Unknown 838	669.3945	3.4581	3.35	0.0154	0.7056
Unknown 1014	728.4874	8.3766	-3.32	0.0161	0.7056
Unknown 535	585.3683	7.7909	-3.3	0.0165	0.7056
Unknown 1519	682.377	1.5301	3.25	0.0175	0.7056
Unknown 930	522.3371	7.8613	-3.19	0.0189	0.7056
Unknown 1315	513.321	7.6436	3.16	0.0197	0.7056
Unknown 813	294.1302	0.9072	-3.13	0.0203	0.7056
Unknown 1495	477.3308	7.9104	-3.12	0.0206	0.7056
Unknown 602	666.3823	2.2502	3.11	0.0207	0.7056
Unknown 1171	789.527	7.9828	-3.1	0.0211	0.7056
Unknown 1449	342.1897	2.7935	-3.05	0.0226	0.7056
Unknown 1187	330.2031	1.6645	3.03	0.023	0.7056
Unknown 524	487.2925	7.7877	-3.03	0.023	0.7056
Unknown 1338	742.5042	8.5139	-3.02	0.0233	0.7056
Unknown 1132	768.4662	3.8924	3.02	0.0233	0.7056
Unknown 1455	410.1778	0.9302	3.01	0.0236	0.7056
Unknown 44	730.4991	8.4890	-3	0.0241	0.7056
Unknown 1262	630.2602	0.8480	2.97	0.0249	0.7056
Unknown 795	449.577	0.5560	-2.93	0.0263	0.7056
Unknown 1189	437.2765	2.2355	2.93	0.0264	0.7056
Unknown 1528	399.2558	2.2401	2.91	0.0269	0.7056
Unknown 187	361.2358	6.8968	-2.9	0.0275	0.7056
Unknown 973	728.4871	8.3019	-2.86	0.0289	0.7056
Unknown 641	761.4974	8.1855	-2.83	0.0301	0.7056
Unknown 1437	789.5274	8.3954	-2.81	0.0306	0.7056
Unknown 1498	619.3194	6.9835	-2.81	0.0309	0.7056
Unknown 1333	810.4913	8.2977	-2.78	0.0321	0.7056
Unknown 1201	629.5163	6.4142	-2.77	0.0325	0.7056
Unknown 1170	502.0437	0.7856	-2.76	0.0331	0.7056
Unknown 1419	613.5206	7.0448	-2.75	0.0334	0.7056
Unknown 990	730.5036	8.7344	-2.74	0.0336	0.7056
Unknown 125	121.0342	1.3545	2.73	0.0341	0.7056
Unknown 1027	709.3943	3.1915	2.73	0.0342	0.7056
Unknown 600	583.2846	0.9150	2.73	0.0343	0.7056
Unknown 572	399.3163	6.3839	-2.72	0.0347	0.7056

Unknown 879	418.2738	6.6756	-2.71	0.0353	0.7056
Unknown 1340	511.3044	6.9382	-2.7	0.0357	0.7056
Unknown 1274	633.4545	8.5067	-2.69	0.036	0.7056
Unknown 875	569.0515	0.6329	-2.69	0.0362	0.7056
Unknown 1403	245.043	8.5559	-2.69	0.0363	0.7056
Unknown 1401	756.5179	8.5399	-2.68	0.0368	0.7056
Unknown 1366	583.2493	0.9197	2.64	0.0385	0.7056
Unknown 1376	832.5688	8.2816	-2.63	0.039	0.7056
Unknown 650	550.3577	3.1316	2.63	0.0391	0.7056
Unknown 1092	849.4895	8.4959	-2.62	0.0394	0.7056
Unknown 585	464.1156	2.1861	2.62	0.0394	0.7056
Unknown 1320	704.4848	8.2856	-2.62	0.0395	0.7056
Unknown 1344	157.1232	3.8498	-2.62	0.0396	0.7056
Unknown 567	538.3232	2.2480	2.62	0.0398	0.7056
Unknown 918	575.3075	2.3785	2.61	0.0402	0.7056
Unknown 284	635.394	7.7405	-2.61	0.0404	0.7056
Unknown 910	707.4497	7.8959	-2.6	0.0407	0.7056
Unknown 1205	808.4504	3.7088	2.59	0.0412	0.7056
Unknown 1200	447.1649	0.8169	2.59	0.0414	0.7056
Unknown 1298	599.5069	7.0337	-2.59	0.0414	0.7056
Unknown 864	807.9489	3.7081	2.58	0.0418	0.7056
Unknown 439	240.2023	6.3330	-2.52	0.0456	0.7056
Unknown 555	560.8065	2.8508	2.52	0.0456	0.7056
Unknown 1343	812.5082	7.0941	-2.51	0.0456	0.7056
Unknown 1052	812.507	8.7347	-2.49	0.0469	0.7056
Unknown 546	363.2256	7.0631	-2.49	0.0471	0.7056
Unknown 992	405.1936	1.0508	2.49	0.0473	0.7056
Unknown 1188	717.4727	8.3116	-2.48	0.0476	0.7056
Unknown 911	540.0558	0.6741	-2.47	0.0482	0.7056
Unknown 1479	763.505	7.6731	-2.47	0.0487	0.7056
Unknown 113	451.3464	8.0870	-2.46	0.0488	0.7056
Unknown 1436	571.3403	2.7107	-2.46	0.0492	0.7056
Unknown 788	581.2425	4.3955	-2.45	0.0499	0.7056

Supplementary Table S5: ANOVA analysis of lipids in biocide treated *P. oleovorans* 1045 strain.

Lipid Name	m/z	Retention time	f.value	p.value	FDR	Tukey's HSD
L-Carnitine	162.1112	0.73	6.2500	0.0085	0.0130	1045- CIT 4-1045 Control
PC(16:0/16:0)	734.5685	6.66	26.4000	0.0000	0.0001	1045- BIT 150-1045 Control
PC(32:1)	732.5524	6.18	34.0000	0.0000	0.0000	1045- BIT 150-1045 Control
PC(32:2)	730.5373	5.77	44.9000	0.0000	0.0000	1045- BIT 150-1045 Control
PC(32:2).1	730.5373	5.77	15.5000	0.0002	0.0005	1045- BIT 150-1045 Control
PC(34:1)	760.5840	6.61	27.4000	0.0000	0.0001	1045- BIT 150-1045 Control
PC(34:2)	758.5689	6.16	35.3000	0.0000	0.0000	1045- BIT 150-1045 Control
PC(34:2).1	758.5689	6.23	32.9000	0.0000	0.0000	1045- BIT 150-1045 Control
PC(35:2)	772.5819	6.43	10.7000	0.0011	0.0020	1045- BIT 150-1045 Control
PC(35:2).1	772.5834	6.49	22.4000	0.0000	0.0001	1045- BIT 150-1045 Control
PC(36:1)	788.6145	7.04	14.3000	0.0003	0.0007	1045- BIT 150-1045 Control
PC(36:2)	786.5997	6.58	29.8000	0.0000	0.0000	1045- BIT 150-1045 Control
PC(36:4)	782.5667	6.6	25.1000	0.0000	0.0001	1045- BIT 150-1045 Control
PC(37:2)	800.6159	6.89	5.7700	0.0111	0.0166	1045- BIT 150-1045 Control
PE(34:2)	716.5222	6.29	26.6000	0.0000	0.0001	1045- BIT 150-1045 Control
PS(41:4)	876.5661	6.58	17.3000	0.0001	0.0003	1045- BIT 150-1045 Control
PS(41:4).1	876.5658	6.56	20.4000	0.0001	0.0002	1045- BIT 150-1045 Control
Unknown 1001	704.5228	5.75	9.0800	0.0021	0.0037	1045- CIT 4-1045- BIT 150
Unknown 1002	313.3574	5.84	4.2700	0.0287	0.0396	1045- MIT 25-1045 Control
Unknown 1003	595.2919	3.17	12.2000	0.0006	0.0012	1045- BIT 150-1045 Control
Unknown 1005	734.5330	6.1	17.8000	0.0001	0.0003	1045- CIT 4-1045 Control
Unknown 1007	374.2429	0.79	12.9000	0.0005	0.0010	1045- BIT 150-1045 Control
Unknown 1008	749.5472	7.3	20.1000	0.0001	0.0002	1045- BIT 150-1045 Control
Unknown 1011	551.5039	6.8	26.9000	0.0000	0.0001	1045- BIT 150-1045 Control
Unknown 1014	183.0936	0.89	8.3200	0.0029	0.0050	1045- BIT 150-1045 Control
Unknown 1015	375.3326	3.39	17.3000	0.0001	0.0003	1045- BIT 150-1045 Control
Unknown 1018	774.5987	6.96	13.6000	0.0004	0.0008	1045- BIT 150-1045 Control
Unknown 1019	339.3370	3.39	20.8000	0.0001	0.0002	1045- BIT 150-1045 Control
Unknown 1023	549.5713	5.53	11.6000	0.0007	0.0015	1045- BIT 150-1045 Control
Unknown 1024	385.3777	3.44	4.8200	0.0199	0.0284	1045- MIT 25-1045- BIT 150
Unknown 1025	645.4883	7.71	40.3000	0.0000	0.0000	1045- BIT 150-1045 Control
Unknown 1027	284.2116	0.82	13.9000	0.0003	0.0008	1045- BIT 150-1045 Control
Unknown 1028	776.4588	6.34	10.8000	0.0010	0.0019	1045- BIT 150-1045 Control
Unknown 1030	714.5063	6.18	7.3300	0.0047	0.0077	1045- CIT 4-1045- BIT 150
Unknown 1033	551.5038	9.65	14.9000	0.0002	0.0006	1045- BIT 150-1045 Control
Unknown 1035	603.5331	9.72	7.6600	0.0040	0.0067	1045- CIT 4-1045- BIT 150
Unknown 1038	136.0484	0.73	38.8000	0.0000	0.0000	1045- CIT 4-1045 Control
Unknown 1039	349.3171	3.36	11.3000	0.0008	0.0016	1045- BIT 150-1045 Control
Unknown 1041	671.5026	7.72	26.5000	0.0000	0.0001	1045- BIT 150-1045 Control
Unknown 1042	807.5124	6.64	18.2000	0.0001	0.0003	1045- BIT 150-1045 Control
Unknown 1043	1004.7875	8.15	6.8700	0.0060	0.0096	1045- CIT 4-1045- BIT 150
Unknown 1045	324.3617	3.43	32.4000	0.0000	0.0000	1045- BIT 150-1045 Control

Unknown 1046	264.1144	0.87	7.6500	0.0040	0.0067	1045- CIT 4-1045- BIT 150
Unknown 1048	579.5336	6.81	21.7000	0.0000	0.0001	1045- BIT 150-1045 Control
Unknown 1050	802.6051	6.68	31.6000	0.0000	0.0000	1045- BIT 150-1045 Control
Unknown 1051	343.1292	0.88	13.2000	0.0004	0.0009	1045- CIT 4-1045 Control
Unknown 1055	392.2528	2.41	35.1000	0.0000	0.0000	1045- BIT 150-1045 Control
Unknown 1060	782.6442	7.66	16.2000	0.0002	0.0004	1045- BIT 150-1045 Control
Unknown 1065	273.1319	0.79	37.1000	0.0000	0.0000	1045- CIT 4-1045 Control
Unknown 1068	1002.7726	7.86	10.7000	0.0010	0.0020	1045- MIT 25-1045 Control
Unknown 1069	575.5020	9.67	19.2000	0.0001	0.0002	1045- BIT 150-1045 Control
Unknown 1071	864.5698	6.61	6.6600	0.0067	0.0106	1045- CIT 4-1045- BIT 150
Unknown 1073	535.3775	7.72	47.1000	0.0000	0.0000	1045- BIT 150-1045 Control
Unknown 1076	764.5421	6.08	7.6300	0.0041	0.0067	1045- BIT 150-1045 Control
Unknown 1077	298.1158	0.81	36.6000	0.0000	0.0000	1045- CIT 4-1045 Control
Unknown 1080	577.5180	8.09	8.3300	0.0029	0.0050	1045- CIT 4-1045- BIT 150
Unknown 1082	256.1730	0.75	8.5100	0.0027	0.0046	1045- CIT 4-1045 Control
Unknown 1085	962.7830	8.34	35.2000	0.0000	0.0000	1045- BIT 150-1045 Control
Unknown 1086	808.6013	6.31	23.8000	0.0000	0.0001	1045- BIT 150-1045 Control
Unknown 1089	692.5218	6.97	11.3000	0.0008	0.0016	1045- BIT 150-1045 Control
Unknown 1090	119.0889	0.72	5.2300	0.0154	0.0225	1045- CIT 4-1045 Control
Unknown 1091	195.1141	0.75	6.0000	0.0097	0.0148	1045- CIT 4-1045 Control
Unknown 1092	747.5710	6.41	9.9500	0.0014	0.0027	1045- CIT 4-1045- BIT 150
Unknown 1093	579.5267	9.21	12.7000	0.0005	0.0011	1045- BIT 150-1045 Control
Unknown 1102	338.1434	0.74	11.6000	0.0007	0.0015	1045- BIT 150-1045 Control
Unknown 1105	120.0809	0.8	16.0000	0.0002	0.0004	1045- CIT 4-1045 Control
Unknown 1106	710.4726	5.94	27.1000	0.0000	0.0001	1045- BIT 150-1045 Control
Unknown 1107	264.1303	0.82	53.6000	0.0000	0.0000	1045- CIT 4-1045 Control
Unknown 1110	807.5832	6.3	12.3000	0.0006	0.0012	1045- CIT 4-1045 Control
Unknown 1113	335.1980	2.69	14.1000	0.0003	0.0007	1045- CIT 4-1045 Control
Unknown 1114	114.0661	0.74	31.1000	0.0000	0.0000	1045- CIT 4-1045 Control
Unknown 1118	530.2968	1.04	78.0000	0.0000	0.0000	1045- CIT 4-1045 Control
Unknown 1125	589.5177	6.16	22.6000	0.0000	0.0001	1045- BIT 150-1045 Control
Unknown 1126	802.4578	6.33	28.1000	0.0000	0.0000	1045- BIT 150-1045 Control
Unknown 1131	707.5386	6.18	14.4000	0.0003	0.0007	1045- BIT 150-1045 Control
Unknown 1132	563.2647	3.5	14.3000	0.0003	0.0007	1045- BIT 150-1045 Control
Unknown 1133	251.1503	0.79	35.6000	0.0000	0.0000	1045- CIT 4-1045 Control
Unknown 1135	752.5425	6.1	25.4000	0.0000	0.0001	1045- BIT 150-1045 Control
Unknown 1138	936.7675	8.4	43.9000	0.0000	0.0000	1045- BIT 150-1045 Control
Unknown 1139	798.5397	6.59	14.3000	0.0003	0.0007	1045- BIT 150-1045 Control
Unknown 1143	785.4680	5.84	42.1000	0.0000	0.0000	1045- BIT 150-1045 Control
Unknown 1144	228.1460	0.74	5.1100	0.0166	0.0239	1045- CIT 4-1045 Control
Unknown 1145	523.3769	7.71	51.4000	0.0000	0.0000	1045- BIT 150-1045 Control
Unknown 1146	735.5375	6.19	40.6000	0.0000	0.0000	1045- BIT 150-1045 Control
Unknown 1148	327.3735	3.44	71.1000	0.0000	0.0000	1045- BIT 150-1045 Control
Unknown 1150	848.5377	6.14	18.9000	0.0001	0.0002	1045- BIT 150-1045 Control

Unknown 1152	727.5652	7.32	15.9000	0.0002	0.0005	1045- BIT 150-1045 Control
Unknown 1153	467.3145	7.72	41.0000	0.0000	0.0000	1045- BIT 150-1045 Control
Unknown 1154	577.5177	9.98	15.5000	0.0002	0.0005	1045- BIT 150-1045 Control
Unknown 1156	713.5501	7.71	63.9000	0.0000	0.0000	1045- BIT 150-1045 Control
Unknown 1157	575.5021	6.35	30.6000	0.0000	0.0000	1045- BIT 150-1045 Control
Unknown 1158	678.5047	6.56	46.2000	0.0000	0.0000	1045- BIT 150-1045 Control
Unknown 1159	855.6986	7.73	28.1000	0.0000	0.0000	1045- BIT 150-1045 Control
Unknown 1160	1030.8035	8.08	12.8000	0.0005	0.0010	1045- CIT 4-1045- BIT 150
Unknown 1161	591.5338	6.65	12.0000	0.0006	0.0013	1045- BIT 150-1045 Control
Unknown 1162	549.4866	5.96	13.5000	0.0004	0.0008	1045- BIT 150-1045 Control
Unknown 1163	313.3574	5.59	4.5300	0.0241	0.0340	1045- MIT 25-1045 Control
Unknown 1164	603.5331	9.55	46.3000	0.0000	0.0000	1045- BIT 150-1045 Control
Unknown 1166	549.4882	9.34	8.3400	0.0029	0.0050	1045- CIT 4-1045- BIT 150
Unknown 1168	752.5425	6.19	25.7000	0.0000	0.0001	1045- BIT 150-1045 Control
Unknown 1169	714.5064	6.11	18.3000	0.0001	0.0003	1045- BIT 150-1045 Control
Unknown 1173	603.4399	7.72	54.2000	0.0000	0.0000	1045- BIT 150-1045 Control
Unknown 1175	885.6726	8.08	82.7000	0.0000	0.0000	1045- BIT 150-1045 Control
Unknown 1176	783.5141	6.19	23.1000	0.0000	0.0001	1045- BIT 150-1045 Control
Unknown 1179	792.5759	6.41	6.9600	0.0057	0.0091	1045- CIT 4-1045- BIT 150
Unknown 1182	577.5180	9.52	19.5000	0.0001	0.0002	1045- BIT 150-1045 Control
Unknown 1183	780.5734	6.63	13.2000	0.0004	0.0009	1045- BIT 150-1045 Control
Unknown 1184	754.5355	6.96	4.0000	0.0346	0.0466	
Unknown 1185	710.4724	5.82	37.8000	0.0000	0.0000	1045- BIT 150-1045 Control
Unknown 1186	551.5033	6.36	32.7000	0.0000	0.0000	1045- BIT 150-1045 Control
Unknown 1190	313.3585	3.64	5.9600	0.0100	0.0151	1045- MIT 25-1045- BIT 150
Unknown 1191	122.0704	0.74	21.8000	0.0000	0.0001	1045- BIT 150-1045 Control
Unknown 1193	710.4724	5.89	46.4000	0.0000	0.0000	1045- BIT 150-1045 Control
Unknown 1197	778.5584	6.16	16.8000	0.0001	0.0004	1045- BIT 150-1045 Control
Unknown 1199	740.5196	6.94	14.4000	0.0003	0.0007	1045- BIT 150-1045 Control
Unknown 1202	339.3725	3.57	16.1000	0.0002	0.0004	1045- BIT 150-1045 Control
Unknown 1203	169.0768	0.89	9.0300	0.0021	0.0038	1045- BIT 150-1045 Control
Unknown 1207	314.3604	3.48	20.9000	0.0001	0.0001	1045- CIT 4-1045 Control
Unknown 1208	140.0683	0.74	10.3000	0.0012	0.0023	1045- CIT 4-1045 Control
Unknown 1209	118.0861	0.71	4.8800	0.0191	0.0273	1045- CIT 4-1045 Control
Unknown 1210	754.5361	6.17	26.5000	0.0000	0.0001	1045- BIT 150-1045 Control
Unknown 1211	226.1544	0.72	9.7100	0.0016	0.0029	1045- CIT 4-1045 Control
Unknown 1213	577.6021	6.05	4.2600	0.0288	0.0397	
Unknown 1218	732.5536	6.95	4.4100	0.0261	0.0365	1045- CIT 4-1045- BIT 150
Unknown 1219	662.4755	5.87	30.9000	0.0000	0.0000	1045- BIT 150-1045 Control
Unknown 1220	257.1008	0.74	33.2000	0.0000	0.0000	1045- CIT 4-1045 Control
Unknown 1222	575.5021	9.09	24.5000	0.0000	0.0001	1045- BIT 150-1045 Control
Unknown 1223	908.5848	6.12	13.6000	0.0004	0.0008	1045- BIT 150-1045 Control
Unknown 1224	908.5849	6.16	10.9000	0.0010	0.0019	1045- BIT 150-1045 Control
Unknown 1225	738.5264	5.92	24.2000	0.0000	0.0001	1045- BIT 150-1045 Control

Unknown 1228	603.5335	6.26	70.4000	0.0000	0.0000	1045- BIT 150-1045 Control
Unknown 1231	880.7170	8.08	78.6000	0.0000	0.0000	1045- BIT 150-1045 Control
Unknown 1232	662.4752	5.81	45.5000	0.0000	0.0000	1045- BIT 150-1045 Control
Unknown 1233	734.4705	6.38	44.7000	0.0000	0.0000	1045- BIT 150-1045 Control
Unknown 1234	603.5333	9.39	28.1000	0.0000	0.0000	1045- BIT 150-1045 Control
Unknown 1235	549.4879	8.89	42.4000	0.0000	0.0000	1045- BIT 150-1045 Control
Unknown 1236	296.3306	2.98	21.7000	0.0000	0.0001	1045- BIT 150-1045 Control
Unknown 1237	322.3462	3.03	45.4000	0.0000	0.0000	1045- BIT 150-1045 Control
Unknown 1240	808.5801	6.58	42.5000	0.0000	0.0000	1045- BIT 150-1045 Control
Unknown 1242	747.5172	5.92	35.0000	0.0000	0.0000	1045- BIT 150-1045 Control
Unknown 1243	734.4699	6.32	26.8000	0.0000	0.0001	1045- BIT 150-1045 Control
Unknown 1244	549.4869	5.86	38.0000	0.0000	0.0000	1045- BIT 150-1045 Control
Unknown 1245	327.3740	3.17	41.3000	0.0000	0.0000	1045- BIT 150-1045 Control
Unknown 1248	736.4861	6.78	56.4000	0.0000	0.0000	1045- BIT 150-1045 Control
Unknown 1249	780.5512	6.16	29.1000	0.0000	0.0000	1045- BIT 150-1045 Control
Unknown 1251	575.5017	5.91	32.3000	0.0000	0.0000	1045- BIT 150-1045 Control
Unknown 1252	776.5904	6.74	181.0000	0.0000	0.0000	1045- BIT 150-1045 Control
Unknown 1254	786.5996	6.67	18.6000	0.0001	0.0002	1045- CIT 4-1045 Control
Unknown 1256	910.6015	6.57	11.9000	0.0007	0.0013	1045- BIT 150-1045 Control
Unknown 1257	743.4823	5.86	34.7000	0.0000	0.0000	1045- BIT 150-1045 Control
Unknown 1258	578.5214	9.47	9.9100	0.0014	0.0027	1045- BIT 150-1045 Control
Unknown 1260	797.5289	6.27	50.1000	0.0000	0.0000	1045- BIT 150-1045 Control
Unknown 1261	756.4927	6.73	145.0000	0.0000	0.0000	1045- BIT 150-1045 Control
Unknown 1262	769.4982	5.94	32.8000	0.0000	0.0000	1045- BIT 150-1045 Control
Unknown 1263	578.5215	9.52	27.7000	0.0000	0.0001	1045- BIT 150-1045 Control
Unknown 1264	749.5327	6.32	40.9000	0.0000	0.0000	1045- BIT 150-1045 Control
Unknown 1265	577.6021	6.02	23.3000	0.0000	0.0001	1045- BIT 150-1045 Control
Unknown 1266	747.5168	5.85	60.1000	0.0000	0.0000	1045- BIT 150-1045 Control
Unknown 1267	744.5534	6.89	10.1000	0.0013	0.0025	1045- BIT 150-1045 Control
Unknown 1268	325.3567	3.05	48.0000	0.0000	0.0000	1045- BIT 150-1045 Control
Unknown 1269	575.5019	5.84	46.6000	0.0000	0.0000	1045- BIT 150-1045 Control
Unknown 1270	760.5739	6.2	9.3300	0.0019	0.0034	1045- BIT 150-1045 Control
Unknown 1271	833.5838	7.71	20.5000	0.0001	0.0002	1045- BIT 150-1045 Control
Unknown 1273	576.5056	8.95	25.3000	0.0000	0.0001	1045- BIT 150-1045 Control
Unknown 1274	854.6898	7.71	26.5000	0.0000	0.0001	1045- BIT 150-1045 Control
Unknown 1275	754.5355	7.04	13.9000	0.0003	0.0008	1045- BIT 150-1045 Control
Unknown 1276	792.5740	6.27	90.5000	0.0000	0.0000	1045- BIT 150-1045 Control
Unknown 1280	738.5048	6.35	54.0000	0.0000	0.0000	1045- BIT 150-1045 Control
Unknown 1281	738.5266	5.85	57.2000	0.0000	0.0000	1045- BIT 150-1045 Control
Unknown 1282	853.6811	7.71	36.6000	0.0000	0.0000	1045- BIT 150-1045 Control
Unknown 1283	761.4894	6.34	55.1000	0.0000	0.0000	1045- BIT 150-1045 Control
Unknown 1285	732.5541	7.04	9.1300	0.0020	0.0037	1045- BIT 150-1045 Control
Unknown 1286	299.3416	3	39.4000	0.0000	0.0000	1045- BIT 150-1045 Control
Unknown 1287	712.4883	6.38	57.2000	0.0000	0.0000	1045- BIT 150-1045 Control

Unknown 1288	692.5215	6.78	40.2000	0.0000	0.0000	1045- BIT 150-1045 Control
Unknown 1289	769.4983	5.86	44.8000	0.0000	0.0000	1045- BIT 150-1045 Control
Unknown 1290	714.5040	6.79	46.3000	0.0000	0.0000	1045- BIT 150-1045 Control
Unknown 1293	764.5428	5.84	34.4000	0.0000	0.0000	1045- BIT 150-1045 Control
Unknown 1295	764.5426	5.9	25.0000	0.0000	0.0001	1045- BIT 150-1045 Control
Unknown 1296	313.3574	2.97	16.7000	0.0001	0.0004	1045- BIT 150-1045 Control
Unknown 1298	339.3725	3.22	28.9000	0.0000	0.0000	1045- BIT 150-1045 Control
Unknown 1299	712.4882	6.31	57.3000	0.0000	0.0000	1045- BIT 150-1045 Control
Unknown 1300	235.1192	0.72	45.7000	0.0000	0.0000	1045- CIT 4-1045 Control
Unknown 1302	313.3574	3.26	22.0000	0.0000	0.0001	1045- BIT 150-1045 Control
Unknown 1303	771.5145	6.32	57.4000	0.0000	0.0000	1045- BIT 150-1045 Control
Unknown 1304	730.5376	6.59	13.7000	0.0004	0.0008	1045- BIT 150-1045 Control
Unknown 1305	730.5376	6.61	13.2000	0.0004	0.0009	1045- BIT 150-1045 Control
Unknown 1306	314.3604	2.97	27.9000	0.0000	0.0001	1045- BIT 150-1045 Control
Unknown 1307	254.1610	0.74	6.8800	0.0060	0.0095	1045- CIT 4-1045 Control
Unknown 1308	690.5067	6.38	29.5000	0.0000	0.0000	1045- BIT 150-1045 Control
Unknown 1309	577.5182	6.3	42.7000	0.0000	0.0000	1045- BIT 150-1045 Control
Unknown 1310	315.3638	2.98	28.4000	0.0000	0.0000	1045- BIT 150-1045 Control
Unknown 1311	339.3726	3.03	38.3000	0.0000	0.0000	1045- BIT 150-1045 Control
Unknown 1312	342.3915	3.42	44.9000	0.0000	0.0000	1045- BIT 150-1045 Control
Unknown 1313	795.6279	7.72	42.9000	0.0000	0.0000	1045- BIT 150-1045 Control
Unknown 1314	719.5420	6.74	55.9000	0.0000	0.0000	1045- BIT 150-1045 Control
Unknown 1315	690.5064	6.31	41.1000	0.0000	0.0000	1045- BIT 150-1045 Control
Unknown 1317	766.5583	6.31	49.5000	0.0000	0.0000	1045- BIT 150-1045 Control
Unknown 1318	340.3759	3.03	42.8000	0.0000	0.0000	1045- BIT 150-1045 Control
Unknown 1321	341.3884	3.42	47.2000	0.0000	0.0000	1045- BIT 150-1045 Control
Unknown 1329	237.1318	0.73	24.9000	0.0000	0.0001	1045- CIT 4-1045 Control
Unknown 1330	354.1173	0.74	5.3900	0.0140	0.0207	1045- BIT 150-1045 Control
Unknown 1339	137.0466	0.75	4.3000	0.0282	0.0391	1045- CIT 4-1045- BIT 150
Unknown 1356	297.0405	0.78	22.6000	0.0000	0.0001	1045- CIT 4-1045 Control
Unknown 1359	299.0385	0.78	9.3800	0.0018	0.0033	1045- CIT 4-1045- BIT 150
Unknown 1361	524.3070	0.78	13.5000	0.0004	0.0009	1045- BIT 150-1045 Control
Unknown 1368	270.1919	0.79	5.7700	0.0111	0.0166	1045- CIT 4-1045 Control
Unknown 1373	354.0992	0.8	5.1800	0.0159	0.0230	1045- CIT 4-1045- BIT 150
Unknown 1375	165.0451	0.81	44.4000	0.0000	0.0000	1045- CIT 4-1045 Control
Unknown 1386	576.3494	0.85	46.5000	0.0000	0.0000	1045- CIT 4-1045 Control
Unknown 1387	638.2711	0.85	81.2000	0.0000	0.0000	1045- CIT 4-1045 Control
Unknown 1388	620.3135	0.86	35.5000	0.0000	0.0000	1045- CIT 4-1045 Control
Unknown 1390	660.2531	0.86	54.1000	0.0000	0.0000	1045- CIT 4-1045 Control
Unknown 1393	558.3275	0.9	62.0000	0.0000	0.0000	1045- CIT 4-1045 Control
Unknown 1396	583.7646	0.92	56.3000	0.0000	0.0000	1045- CIT 4-1045 Control
Unknown 1397	272.2122	0.92	24.8000	0.0000	0.0001	1045- BIT 150-1045 Control
Unknown 1398	686.3970	0.92	58.5000	0.0000	0.0000	1045- BIT 150-1045 Control
Unknown 1400	439.2905	0.94	14.6000	0.0003	0.0006	1045- CIT 4-1045 Control

Unknown 1404	627.3489	0.95	27.1000	0.0000	0.0001	1045- CIT 4-1045 Control
Unknown 1411	152.0153	1.04	75.3000	0.0000	0.0000	1045- BIT 150-1045 Control
Unknown 1447	409.2793	2.41	23.3000	0.0000	0.0001	1045- BIT 150-1045 Control
Unknown 1448	285.3259	2.54	20.3000	0.0001	0.0002	1045- BIT 150-1045 Control
Unknown 1450	311.3414	2.64	17.2000	0.0001	0.0003	1045- BIT 150-1045 Control
Unknown 1451	294.3146	2.64	38.5000	0.0000	0.0000	1045- BIT 150-1045 Control
Unknown 1453	329.3522	2.69	69.7000	0.0000	0.0000	1045- BIT 150-1045 Control
Unknown 1455	616.1757	2.74	12.0000	0.0006	0.0013	1045- BIT 150-1045 Control
Unknown 1456	299.3416	2.75	69.4000	0.0000	0.0000	1045- BIT 150-1045 Control
Unknown 1458	325.3573	2.86	60.3000	0.0000	0.0000	1045- BIT 150-1045 Control
Unknown 147	718.2270	5.31	4.1900	0.0302	0.0411	1045- CIT 4-1045- BIT 150
Unknown 1471	353.3883	3.04	27.6000	0.0000	0.0001	1045- BIT 150-1045 Control
Unknown 1478	336.3341	3.2	20.6000	0.0001	0.0002	1045- CIT 4-1045 Control
Unknown 1479	353.3882	3.22	48.3000	0.0000	0.0000	1045- BIT 150-1045 Control
Unknown 1485	355.4040	3.4	22.1000	0.0000	0.0001	1045- CIT 4-1045 Control
Unknown 1493	367.4040	3.43	42.9000	0.0000	0.0000	1045- BIT 150-1045 Control
Unknown 1495	585.2466	3.49	12.0000	0.0006	0.0013	1045- BIT 150-1045 Control
Unknown 1501	355.4037	3.67	23.4000	0.0000	0.0001	1045- BIT 150-1045 Control
Unknown 1533	450.4303	4.41	8.6500	0.0025	0.0044	1045- BIT 150-1045 Control
Unknown 1567	1006.7650	5.36	5.6000	0.0123	0.0184	1045- BIT 150-1045 Control
Unknown 1598	688.4907	5.82	60.4000	0.0000	0.0000	1045- BIT 150-1045 Control
Unknown 1601	995.4407	5.83	49.3000	0.0000	0.0000	1045- BIT 150-1045 Control
Unknown 1602	927.4546	5.84	41.9000	0.0000	0.0000	1045- BIT 150-1045 Control
Unknown 1603	859.4673	5.84	55.5000	0.0000	0.0000	1045- BIT 150-1045 Control
Unknown 1604	791.4799	5.84	88.8000	0.0000	0.0000	1045- BIT 150-1045 Control
Unknown 1609	765.4661	5.86	42.3000	0.0000	0.0000	1045- BIT 150-1045 Control
Unknown 1610	927.4543	5.89	24.6000	0.0000	0.0001	1045- BIT 150-1045 Control
Unknown 1611	859.4673	5.89	63.8000	0.0000	0.0000	1045- BIT 150-1045 Control
Unknown 1612	688.4911	5.9	62.1000	0.0000	0.0000	1045- BIT 150-1045 Control
Unknown 1614	791.4799	5.9	44.7000	0.0000	0.0000	1045- BIT 150-1045 Control
Unknown 1615	765.4664	5.91	33.6000	0.0000	0.0000	1045- BIT 150-1045 Control
Unknown 1617	688.4916	5.98	36.2000	0.0000	0.0000	1045- BIT 150-1045 Control
Unknown 1620	603.6177	6.01	12.5000	0.0005	0.0011	1045- BIT 150-1045 Control
Unknown 1622	903.5348	6.04	10.1000	0.0013	0.0025	1045- CIT 4-1045 Control
Unknown 1629	688.4930	6.08	30.2000	0.0000	0.0000	1045- BIT 150-1045 Control
Unknown 1635	702.5077	6.09	39.7000	0.0000	0.0000	1045- BIT 150-1045 Control
Unknown 1641	952.5486	6.12	8.8800	0.0023	0.0040	1045- BIT 150-1045 Control
Unknown 1643	930.5662	6.13	13.1000	0.0004	0.0009	1045- BIT 150-1045 Control
Unknown 1645	952.5485	6.14	8.8300	0.0023	0.0041	1045- BIT 150-1045 Control
Unknown 1646	702.5073	6.14	37.6000	0.0000	0.0000	1045- BIT 150-1045 Control
Unknown 1647	882.5693	6.15	19.2000	0.0001	0.0002	1045- BIT 150-1045 Control
Unknown 1648	930.5659	6.15	14.1000	0.0003	0.0007	1045- BIT 150-1045 Control
Unknown 1650	873.4830	6.16	21.0000	0.0001	0.0001	1045- BIT 150-1045 Control
Unknown 1652	882.5692	6.18	22.0000	0.0000	0.0001	1045- BIT 150-1045 Control

Unknown 1660	828.4727	6.28	23.6000	0.0000	0.0001	1045- BIT 150-1045 Control
Unknown 1661	896.4601	6.28	21.5000	0.0000	0.0001	1045- BIT 150-1045 Control
Unknown 1662	964.4476	6.29	26.2000	0.0000	0.0001	1045- BIT 150-1045 Control
Unknown 1663	787.4852	6.29	29.8000	0.0000	0.0000	1045- BIT 150-1045 Control
Unknown 1665	754.4761	6.3	37.9000	0.0000	0.0000	1045- BIT 150-1045 Control
Unknown 1666	800.4753	6.3	45.2000	0.0000	0.0000	1045- BIT 150-1045 Control
Unknown 1667	822.4570	6.3	14.0000	0.0003	0.0007	1045- BIT 150-1045 Control
Unknown 1669	793.4961	6.31	55.2000	0.0000	0.0000	1045- BIT 150-1045 Control
Unknown 1670	929.4702	6.31	22.6000	0.0000	0.0001	1045- BIT 150-1045 Control
Unknown 1671	861.4832	6.31	33.5000	0.0000	0.0000	1045- BIT 150-1045 Control
Unknown 1672	686.4715	6.31	38.6000	0.0000	0.0000	1045- BIT 150-1045 Control
Unknown 1673	997.4565	6.31	4.6100	0.0228	0.0323	1045- BIT 150-1045 Control
Unknown 1674	664.4897	6.31	20.2000	0.0001	0.0002	1045- BIT 150-1045 Control
Unknown 1675	870.4452	6.32	4.4800	0.0250	0.0352	1045- BIT 150-1045 Control
Unknown 1683	828.4728	6.35	26.0000	0.0000	0.0001	1045- BIT 150-1045 Control
Unknown 1685	896.4600	6.35	31.8000	0.0000	0.0000	1045- BIT 150-1045 Control
Unknown 1700	726.5041	6.53	15.8000	0.0002	0.0005	1045- BIT 150-1045 Control
Unknown 1703	936.6162	6.55	8.8200	0.0023	0.0041	1045- BIT 150-1045 Control
Unknown 1708	954.5643	6.58	26.2000	0.0000	0.0001	1045- BIT 150-1045 Control
Unknown 1709	932.5815	6.58	28.7000	0.0000	0.0000	1045- BIT 150-1045 Control
Unknown 1711	798.1624	6.59	32.2000	0.0000	0.0000	1045- BIT 150-1045 Control
Unknown 1712	842.4922	6.59	7.0100	0.0056	0.0089	1045- BIT 150-1045 Control
Unknown 1713	910.4783	6.6	5.4000	0.0139	0.0206	1045- BIT 150-1045 Control
Unknown 1715	752.5205	6.6	34.0000	0.0000	0.0000	1045- BIT 150-1045 Control
Unknown 1717	818.6372	6.61	30.2000	0.0000	0.0000	1045- BIT 150-1045 Control
Unknown 1718	850.5542	6.61	35.5000	0.0000	0.0000	1045- BIT 150-1045 Control
Unknown 1719	986.5279	6.61	27.7000	0.0000	0.0001	1045- BIT 150-1045 Control
Unknown 1722	771.5742	6.62	4.9800	0.0180	0.0258	1045- CIT 4-1045- BIT 150
Unknown 1723	704.5229	6.62	12.4000	0.0006	0.0012	1045- BIT 150-1045 Control
Unknown 1728	856.5041	6.69	42.4000	0.0000	0.0000	1045- BIT 150-1045 Control
Unknown 1736	966.4628	6.77	18.0000	0.0001	0.0003	1045- BIT 150-1045 Control
Unknown 1738	966.4630	6.77	17.3000	0.0001	0.0003	1045- BIT 150-1045 Control
Unknown 1742	768.5680	6.78	20.9000	0.0001	0.0001	1045- BIT 150-1045 Control
Unknown 1743	804.4741	6.79	5.8300	0.0107	0.0162	1045- BIT 150-1045 Control
Unknown 1744	750.5716	6.79	5.3300	0.0145	0.0213	1045- MIT 25-1045- BIT 150
Unknown 1747	753.5039	6.84	12.6000	0.0005	0.0011	1045- BIT 150-1045 Control
Unknown 1748	1152.0223	6.84	26.5000	0.0000	0.0001	1045- BIT 150-1045 Control
Unknown 175	937.5787	4.37	6.0700	0.0094	0.0144	1045- BIT 150-1045 Control
Unknown 1751	826.6340	6.85	8.0600	0.0033	0.0056	1045- CIT 4-1045- BIT 150
Unknown 1756	1167.0218	6.89	20.9000	0.0001	0.0001	1045- CIT 4-1045 Control
Unknown 1757	718.5406	6.9	8.7900	0.0023	0.0041	1045- CIT 4-1045- BIT 150
Unknown 1772	776.5187	7.04	24.1000	0.0000	0.0001	1045- BIT 150-1045 Control
Unknown 1777	768.5496	7.13	70.7000	0.0000	0.0000	1045- BIT 150-1045 Control
Unknown 1789	744.5918	7.31	25.1000	0.0000	0.0001	1045- BIT 150-1045 Control

Unknown 1798	839.6667	7.71	13.3000	0.0004	0.0009	1045- BIT 150-1045 Control
Unknown 1800	817.6104	7.72	33.8000	0.0000	0.0000	1045- BIT 150-1045 Control
Unknown 1801	812.6550	7.72	45.7000	0.0000	0.0000	1045- BIT 150-1045 Control
Unknown 1807	1028.7889	7.79	14.3000	0.0003	0.0007	1045- CIT 4-1045 Control
Unknown 1808	901.6463	8.06	32.0000	0.0000	0.0000	1045- BIT 150-1045 Control
Unknown 245	763.6016	7.68	5.3200	0.0146	0.0214	1045- BIT 150-1045 Control
Unknown 246	411.3931	3.51	8.9500	0.0022	0.0039	1045- CIT 4-1045- BIT 150
Unknown 255	149.0231	2.88	6.5500	0.0071	0.0112	1045- BIT 150-1045 Control
Unknown 260	132.1016	0.74	8.7400	0.0024	0.0042	1045- CIT 4-1045 Control
Unknown 266	110.0706	0.72	14.5000	0.0003	0.0007	1045- CIT 4-1045 Control
Unknown 283	631.5373	5.8	8.6600	0.0025	0.0044	1045- CIT 4-1045- BIT 150
Unknown 311	631.5539	7.67	5.1000	0.0167	0.0240	1045- CIT 4-1045- BIT 150
Unknown 317	863.6871	8.09	9.3300	0.0018	0.0034	1045- MIT 25-1045 Control
Unknown 318	798.7437	8.91	7.1200	0.0053	0.0085	1045- BIT 150-1045 Control
Unknown 325	335.1992	2.89	12.1000	0.0006	0.0013	1045- CIT 4-1045 Control
Unknown 332	231.0850	0.75	8.2900	0.0030	0.0051	1045- CIT 4-1045- BIT 150
Unknown 347	354.3721	3.85	7.0300	0.0055	0.0089	1045- CIT 4-1045- BIT 150
Unknown 367	430.3293	3.96	5.5700	0.0125	0.0186	1045- BIT 150-1045 Control
Unknown 399	325.3208	3.05	4.2000	0.0302	0.0411	
Unknown 402	353.3524	3.42	4.2000	0.0300	0.0411	1045- CIT 4-1045- BIT 150
Unknown 403	725.5557	6.33	5.1600	0.0161	0.0233	1045- MIT 25-1045 Control
Unknown 410	557.4444	3.2	6.4900	0.0074	0.0115	1045- BIT 150-1045 Control
Unknown 433	846.6372	7.53	13.3000	0.0004	0.0009	1045- BIT 150-1045 Control
Unknown 456	100.0763	0.77	4.2000	0.0300	0.0411	1045- CIT 4-1045- BIT 150
Unknown 481	355.2929	2.9	20.5000	0.0001	0.0002	1045- CIT 4-1045 Control
Unknown 486	651.3415	4.34	6.3400	0.0081	0.0125	1045- BIT 150-1045 Control
Unknown 505	279.2312	2.71	4.1200	0.0318	0.0431	1045- MIT 25-1045 Control
Unknown 507	747.5745	7.18	6.0200	0.0096	0.0147	1045- CIT 4-1045- BIT 150
Unknown 513	327.3367	3.35	5.5100	0.0130	0.0192	1045- CIT 4-1045- BIT 150
Unknown 516	629.3595	4.34	9.5600	0.0017	0.0031	1045- BIT 150-1045 Control
Unknown 537	382.3774	2.7	10.6000	0.0011	0.0021	1045- BIT 150-1045 Control
Unknown 541	591.5339	9.84	4.0400	0.0337	0.0454	1045- MIT 25-1045- BIT 150
Unknown 554	361.3187	3.38	9.8300	0.0015	0.0028	1045- BIT 150-1045 Control
Unknown 574	745.5066	6.49	5.1900	0.0158	0.0230	1045- CIT 4-1045- BIT 150
Unknown 579	258.1076	0.73	29.3000	0.0000	0.0000	1045- CIT 4-1045 Control
Unknown 603	687.4129	4.35	7.4800	0.0044	0.0072	1045- BIT 150-1045 Control
Unknown 605	229.1022	0.86	17.1000	0.0001	0.0003	1045- CIT 4-1045 Control
Unknown 607	387.2519	7.71	7.3000	0.0048	0.0078	1045- MIT 25-1045 Control
Unknown 609	706.5361	6.94	4.0600	0.0331	0.0448	
Unknown 613	797.5303	6.48	10.9000	0.0010	0.0019	1045- CIT 4-1045- BIT 150
Unknown 617	719.5436	7.02	6.7800	0.0063	0.0100	1045- CIT 4-1045 Control
Unknown 619	849.5992	6.61	4.2800	0.0285	0.0394	1045- CIT 4-1045 Control
Unknown 637	455.3144	7.7	10.9000	0.0010	0.0019	1045- MIT 25-1045 Control
Unknown 647	786.6280	7.31	8.1400	0.0032	0.0054	1045- BIT 150-1045 Control

Unknown 664	374.1793	0.74	6.3600	0.0080	0.0124	1045- CIT 4-1045 Control
Unknown 670	356.3135	2.68	8.8500	0.0023	0.0041	1045- CIT 4-1045 Control
Unknown 675	736.5033	5.43	7.4700	0.0044	0.0072	1045- CIT 4-1045 Control
Unknown 678	319.2730	2.65	16.0000	0.0002	0.0004	1045- CIT 4-1045 Control
Unknown 682	104.1068	0.73	7.0500	0.0055	0.0088	1045- CIT 4-1045- BIT 150
Unknown 692	615.4918	6.84	6.1700	0.0088	0.0136	1045- MIT 25-1045 Control
Unknown 693	577.5196	6.79	4.2600	0.0290	0.0398	
Unknown 697	722.5170	7.66	7.6600	0.0040	0.0067	1045- BIT 150-1045 Control
Unknown 703	369.4204	3.96	8.1100	0.0032	0.0055	1045- CIT 4-1045- BIT 150
Unknown 71	296.2569	2.68	8.5700	0.0026	0.0045	1045- CIT 4-1045 Control
Unknown 710	176.0661	0.76	12.0000	0.0006	0.0013	1045- CIT 4-1045 Control
Unknown 712	166.0902	0.79	6.0500	0.0094	0.0144	1045- MIT 25-1045 Control
Unknown 715	155.0809	0.77	16.0000	0.0002	0.0004	1045- CIT 4-1045 Control
Unknown 721	194.1254	0.74	5.2500	0.0152	0.0222	1045- CIT 4-1045 Control
Unknown 730	737.4947	6.76	9.6200	0.0016	0.0030	1045- BIT 150-1045 Control
Unknown 733	816.6207	6.19	4.9700	0.0181	0.0260	1045- CIT 4-1045- BIT 150
Unknown 734	692.5232	7.13	5.2000	0.0157	0.0229	1045- MIT 25-1045- BIT 150
Unknown 738	459.2595	0.88	12.7000	0.0005	0.0011	1045- BIT 150-1045 Control
Unknown 741	181.0966	0.87	18.2000	0.0001	0.0003	1045- CIT 4-1045 Control
Unknown 744	197.0700	0.84	16.5000	0.0002	0.0004	1045- BIT 150-1045 Control
Unknown 745	250.1158	0.82	14.4000	0.0003	0.0007	1045- CIT 4-1045 Control
Unknown 749	211.1397	0.99	18.7000	0.0001	0.0002	1045- CIT 4-1045 Control
Unknown 753	706.5365	6.98	3.9200	0.0365	0.0491	1045- MIT 25-1045- BIT 150
Unknown 758	575.5005	6.17	6.2900	0.0083	0.0128	1045- CIT 4-1045- BIT 150
Unknown 760	288.1487	0.78	4.1800	0.0305	0.0414	1045- CIT 4-1045- BIT 150
Unknown 762	652.4041	0.8	30.8000	0.0000	0.0000	1045- CIT 4-1045 Control
Unknown 765	197.1281	0.84	25.1000	0.0000	0.0001	1045- CIT 4-1045 Control
Unknown 768	265.0994	1.2	7.8600	0.0036	0.0061	1045- BIT 150-1045 Control
Unknown 774	198.1039	0.99	6.6100	0.0069	0.0109	1045- CIT 4-1045- BIT 150
Unknown 779	503.3270	5.31	7.0200	0.0056	0.0089	1045- BIT 150-1045 Control
Unknown 780	297.2903	2.65	28.6000	0.0000	0.0000	1045- CIT 4-1045 Control
Unknown 781	319.2242	2.69	11.5000	0.0008	0.0015	1045- CIT 4-1045 Control
Unknown 786	631.4696	6.85	7.8000	0.0038	0.0063	1045- CIT 4-1045 Control
Unknown 788	137.0676	0.73	4.6300	0.0225	0.0320	1045- CIT 4-1045- BIT 150
Unknown 792	816.6206	6.26	4.4400	0.0255	0.0359	1045- CIT 4-1045- BIT 150
Unknown 799	766.5352	6.89	5.3500	0.0143	0.0210	1045- CIT 4-1045- BIT 150
Unknown 803	169.0964	0.79	9.0700	0.0021	0.0037	1045- CIT 4-1045 Control
Unknown 805	454.2924	3.41	7.7200	0.0039	0.0065	1045- CIT 4-1045 Control
Unknown 813	688.4224	4.34	4.4300	0.0258	0.0362	1045- CIT 4-1045- BIT 150
Unknown 830	355.2936	2.68	10.2000	0.0013	0.0025	1045- CIT 4-1045 Control
Unknown 835	659.5049	7.72	17.4000	0.0001	0.0003	1045- CIT 4-1045 Control
Unknown 841	134.1168	0.73	31.5000	0.0000	0.0000	1045- BIT 150-1045 Control
Unknown 845	699.5343	7.71	12.3000	0.0006	0.0012	1045- CIT 4-1045 Control
Unknown 851	796.5817	6.84	8.6600	0.0025	0.0044	1045- BIT 150-1045 Control

Unknown 852	953.7974	8.38	21.3000	0.0000	0.0001	1045- BIT 150-1045 Control
Unknown 854	758.5666	6.57	6.0300	0.0096	0.0146	1045- CIT 4-1045- BIT 150
Unknown 856	708.5170	6.23	8.2600	0.0030	0.0051	1045- CIT 4-1045- BIT 150
Unknown 857	214.1352	1.07	4.2900	0.0283	0.0392	1045- CIT 4-1045- BIT 150
Unknown 858	549.5715	5.58	9.4700	0.0017	0.0032	1045- BIT 150-1045 Control
Unknown 859	610.5403	6.84	11.9000	0.0007	0.0013	1045- CIT 4-1045- BIT 150
Unknown 862	450.3065	2.42	12.7000	0.0005	0.0011	1045- BIT 150-1045 Control
Unknown 863	276.1411	0.75	4.3600	0.0271	0.0377	1045- CIT 4-1045 Control
Unknown 869	785.6198	7.32	15.8000	0.0002	0.0005	1045- BIT 150-1045 Control
Unknown 871	229.0801	1.28	13.3000	0.0004	0.0009	1045- BIT 150-1045 Control
Unknown 879	676.4928	6.09	7.5500	0.0042	0.0070	1045- CIT 4-1045- BIT 150
Unknown 882	326.2072	0.81	15.8000	0.0002	0.0005	1045- BIT 150-1045 Control
Unknown 884	209.1277	0.74	5.9000	0.0103	0.0156	1045- CIT 4-1045 Control
Unknown 890	262.0975	0.85	10.1000	0.0013	0.0025	1045- CIT 4-1045- BIT 150
Unknown 893	748.5595	6.35	4.7200	0.0212	0.0302	1045- MIT 25-1045 Control
Unknown 895	824.5394	6.65	6.5900	0.0070	0.0110	1045- CIT 4-1045- BIT 150
Unknown 896	577.4224	7.72	19.9000	0.0001	0.0002	1045- BIT 150-1045 Control
Unknown 898	414.2352	2.41	12.0000	0.0007	0.0013	1045- BIT 150-1045 Control
Unknown 905	301.1203	0.83	24.3000	0.0000	0.0001	1045- BIT 150-1045 Control
Unknown 906	360.1917	0.83	7.8400	0.0037	0.0062	1045- BIT 150-1045 Control
Unknown 907	772.5847	7.29	11.6000	0.0007	0.0015	1045- BIT 150-1045 Control
Unknown 909	502.2901	3.46	12.0000	0.0007	0.0013	1045- CIT 4-1045 Control
Unknown 913	273.0753	0.74	5.7100	0.0115	0.0173	1045- MIT 25-1045 Control
Unknown 915	730.5375	5.92	21.1000	0.0000	0.0001	1045- BIT 150-1045 Control
Unknown 916	979.8096	8.34	12.2000	0.0006	0.0012	1045- BIT 150-1045 Control
Unknown 918	563.5021	6.11	18.3000	0.0001	0.0003	1045- BIT 150-1045 Control
Unknown 919	958.7498	8.39	8.1600	0.0031	0.0054	1045- BIT 150-1045 Control
Unknown 920	603.5328	6.47	17.5000	0.0001	0.0003	1045- CIT 4-1045 Control
Unknown 924	598.3310	0.86	41.4000	0.0000	0.0000	1045- CIT 4-1045 Control
Unknown 928	229.1530	0.8	10.7000	0.0010	0.0020	1045- CIT 4-1045 Control
Unknown 930	549.4868	6.31	19.7000	0.0001	0.0002	1045- BIT 150-1045 Control
Unknown 932	563.5020	6.18	23.4000	0.0000	0.0001	1045- BIT 150-1045 Control
Unknown 939	700.4876	6.56	9.0100	0.0021	0.0038	1045- MIT 25-1045 Control
Unknown 949	1035.7593	8.09	11.1000	0.0009	0.0018	1045- CIT 4-1045- BIT 150
Unknown 951	676.4932	6.14	7.3000	0.0048	0.0078	1045- CIT 4-1045- BIT 150
Unknown 956	209.1034	0.69	34.2000	0.0000	0.0000	1045- CIT 4-1045 Control
Unknown 961	921.7436	8.07	49.8000	0.0000	0.0000	1045- BIT 150-1045 Control
Unknown 962	805.4973	6.16	15.2000	0.0002	0.0005	1045- BIT 150-1045 Control
Unknown 963	828.6502	6.92	8.4000	0.0028	0.0049	1045- CIT 4-1045- BIT 150
Unknown 966	473.2742	1.03	11.1000	0.0009	0.0017	1045- CIT 4-1045 Control
Unknown 972	267.0771	0.75	34.1000	0.0000	0.0000	1045- CIT 4-1045 Control
Unknown 973	410.4089	3.12	17.1000	0.0001	0.0003	1045- BIT 150-1045 Control
Unknown 974	339.3373	3.41	7.5300	0.0043	0.0071	1045- BIT 150-1045 Control
Unknown 976	509.3612	7.73	42.3000	0.0000	0.0000	1045- BIT 150-1045 Control

Unknown 977	371.2390	0.74	4.4000	0.0262	0.0366	1045- CIT 4-1045 Control
Unknown 978	730.5374	5.82	24.6000	0.0000	0.0001	1045- BIT 150-1045 Control
Unknown 980	724.4895	6.14	11.1000	0.0009	0.0018	1045- CIT 4-1045- BIT 150
Unknown 982	704.5225	5.7	6.5200	0.0073	0.0114	1045- CIT 4-1045- BIT 150
Unknown 983	245.1294	0.97	11.5000	0.0008	0.0015	1045- CIT 4-1045 Control
Unknown 985	103.0537	0.8	18.3000	0.0001	0.0003	1045- CIT 4-1045 Control
Unknown 986	728.4614	6.3	15.0000	0.0002	0.0006	1045- BIT 150-1045 Control
Unknown 987	806.5871	5.85	15.8000	0.0002	0.0005	1045- BIT 150-1045 Control
Unknown 988	313.3572	6.52	18.6000	0.0001	0.0002	1045- CIT 4-1045 Control
Unknown 993	320.1831	2.45	13.0000	0.0005	0.0010	1045- CIT 4-1045 Control
Unknown 995	509.5029	4.45	33.1000	0.0000	0.0000	1045- BIT 150-1045 Control
Unknown 997	591.4395	7.73	39.6000	0.0000	0.0000	1045- BIT 150-1045 Control
Unknown 998	856.7040	7.73	16.6000	0.0001	0.0004	1045- BIT 150-1045 Control
Unknown 999	869.6754	6.8	3.9100	0.0368	0.0493	1045- MIT 25-1045- BIT 150

Supplementary Table S6: ANOVA analysis of polar metabolites biocide treated *P. oleovorans* 1045 strain.

Polar metabolite Name	m/z	Retention time	f.value	p.value	FDR	Tukey's HSD (top 1000)
Tetradecenoic acid	225.1858	5.9112	24	0	0.0002	1045- BIT 150-1045 -Control
5-Hydroxyindole-3-acetic acid	190.0479	0.8027	21	0	0.0003	1045- BIT 150-1045 -Control
Tetradecenoic acid	225.1858	5.9112	19.4	0.0001	0.0004	1045- BIT 150-1045 -Control
LPE (16:0)	451.2673	6.7466	18.2	0.0001	0.0005	1045- BIT 150-1045 -Control
Hexadecenoic acid	253.2171	6.7187	17.2	0.0001	0.0006	1045- BIT 150-1045 -Control
Uric acid	167.0245	0.7616	14.1	0.0003	0.0012	1045- BIT 150-1045 -Control
myristic acid	227.2015	6.4492	13.9	0.0003	0.0013	1045- BIT 150-1045 -Control
Genistein	269.0472	3.2876	11	0.0009	0.0027	1045- BIT 150-1045 -Control
5-Oxoproline	128.0324	0.6508	10.1	0.0014	0.0036	1045- CIT 4-1045 -Control
tryptophan	203.0658	0.9257	8.11	0.0032178	0.0072	1045- CIT 4-1045 -Control
GLCA-3S	512.2704	1.039	7.3	0.0048204	0.0095	1045- CIT 4-1045 -Control
Phenylalanine	164.0771	0.865	7.21	0.0050459	0.0098	1045- CIT 4-1045 -Control
Phenylalanine.1	164.0776	0.8834	7.19	0.0050864	0.0098	1045- CIT 4-1045 -Control
C18:2	279.2327	6.8975	6.8	0.006241	0.0116	1045- MIT 25-1045 -Control
5-Methyluridine	257.063	0.5996	6.71	0.006547	0.012	1045- CIT 4-1045- BIT 150
Methionine	148.0486	0.8031	6.43	0.0076338	0.0136	1045- CIT 4-1045 -Control
Glutamic acid	146.0471	0.645	5.76	0.011158	0.0187	1045- CIT 4-1045 -Control
Valine	116.0678	0.7636	5.13	0.01633	0.0256	
Aminobutyric acid	102.055	0.6358	4.91	0.018785	0.0286	
palmitic acid	255.2328	7.1274	4.22	0.029597	0.0426	
Genistein	269.0472	3.2876	4.19	0.03034	0.0435	
Unknown 1	92.9279	0.6836	12.5	0.0005	0.0018	1045- BIT 150-1045 -Control
Unknown 10	677.4178	6.5649	4.01	0.03442	0.0487	
Unknown 1001	669.5287	8.741	23.1	0	0.0002	1045- BIT 150-1045 -Control
Unknown 1002	574.3157	7.647	6.21	0.0086199	0.0151	1045- CIT 4-1045- BIT 150
Unknown 1006	296.2561	6.9179	6.89	0.005961	0.0112	1045- BIT 150-1045 -Control
Unknown 1007	567.2863	6.5022	6.26	0.008397	0.0147	1045- CIT 4-1045- BIT 150
Unknown 1008	690.3356	2.4146	6.98	0.0056931	0.0107	1045- CIT 4-1045 -Control
Unknown 1009	360.2156	0.8219	6.93	0.0058386	0.011	1045- CIT 4-1045- BIT 150
Unknown 101	470.2101	0.861	6.05	0.009469	0.0161	1045- CIT 4-1045 -Control
Unknown 1010	365.1922	4.9725	8.68	0.0024727	0.0058	1045- CIT 4-1045- BIT 150
Unknown 1011	570.3281	2.8473	8.12	0.0032015	0.0071	1045- CIT 4-1045 -Control
Unknown 1013	735.4763	7.2651	5.54	0.012775	0.0208	
Unknown 1014	728.4874	8.3766	18.8	0.0001	0.0005	1045- BIT 150-1045 -Control
Unknown 1015	434.0559	0.8069	15.2	0.0002	0.001	1045- BIT 150-1045 -Control
Unknown 1018	631.3364	6.9312	7.26	0.0049222	0.0096	1045- BIT 150-1045 -Control
Unknown 1019	575.3731	7.8449	25	0	0.0002	1045- BIT 150-1045 -Control
Unknown 102	421.2264	6.0477	4.88	0.019119	0.0291	
Unknown 1021	810.3857	2.6614	11.7	0.0007	0.0022	1045- CIT 4-1045 -Control
Unknown 1023	586.2841	1.0209	5.5	0.013024	0.0212	
Unknown 1025	355.1435	5.0424	10.6	0.0011	0.0031	1045- BIT 150-1045 -Control
Unknown 1027	709.3943	3.1915	13.6	0.0004	0.0013	1045- CIT 4-1045 -Control
Unknown 1028	655.4253	8.4	18.3	0.0001	0.0005	1045- BIT 150-1045 -Control
Unknown 1029	539.4291	4.9547	4.57	0.023502	0.0349	
Unknown 103	611.4267	6.6424	51.4	0	0	1045- BIT 150-1045 -Control
Unknown 1030	283.2321	5.293	7.89	0.0035836	0.0077	1045- MIT 25-1045 -Control

Unknown 1031	547.4339	6.4329	11.3	0.0008	0.0025	1045- MIT 25-1045 -Control
Unknown 1032	595.4938	5.8486	10	0.0014	0.0037	1045- BIT 150-1045 -Control
Unknown 1033	281.2485	7.5826	8.83	0.0023046	0.0055	1045- BIT 150-1045 -Control
Unknown 1036	785.4611	8.2958	33.5	0	0.0001	1045- BIT 150-1045 -Control
Unknown 1037	285.1957	4.4119	5.95	0.01003	0.017	1045- CIT 4-1045- BIT 150
Unknown 104	693.4294	6.6374	23.6	0	0.0002	1045- BIT 150-1045 -Control
Unknown 1040	653.2657	2.4727	6.65	0.006765	0.0123	1045- BIT 150-1045 -Control
Unknown 1041	391.1951	0.9668	5.11	0.016604	0.026	
Unknown 1042	669.2588	2.7703	4.12	0.031879	0.0454	
Unknown 1043	417.2301	7.0001	12.5	0.0005	0.0018	1045- MIT 25-1045 -Control
Unknown 1045	483.194	4.1007	8.96	0.0022	0.0052	1045- CIT 4-1045- BIT 150
Unknown 1047	558.2574	0.9784	5.2	0.015698	0.0248	
Unknown 1048	420.0408	0.7818	11.6	0.0007	0.0023	1045- BIT 150-1045 -Control
Unknown 1049	539.3528	7.7799	56	0	0	1045- BIT 150-1045 -Control
Unknown 1050	629.3205	2.3454	7.47	0.0044172	0.0089	1045- CIT 4-1045 -Control
Unknown 1052	812.507	8.7347	18	0.0001	0.0005	1045- CIT 4-1045 -Control
Unknown 1053	673.2936	6.6127	25.8	0	0.0002	1045- BIT 150-1045 -Control
Unknown 1054	213.1494	3.0805	12.6	0.0005	0.0018	1045- BIT 150-1045 -Control
Unknown 1055	604.3998	8.2805	19	0.0001	0.0005	1045- BIT 150-1045 -Control
Unknown 1056	293.1772	6.0998	11	0.0009	0.0027	1045- CIT 4-1045- BIT 150
Unknown 1057	770.5313	7.3893	17.7	0.0001	0.0006	1045- BIT 150-1045 -Control
Unknown 1059	386.3271	7.8849	4.22	0.029662	0.0427	
Unknown 106	549.2443	5.973	13.3	0.0004	0.0014	1045- BIT 150-1045 -Control
Unknown 1060	731.5	8.3298	10.3	0.0012	0.0034	1045- BIT 150-1045 -Control
Unknown 1061	731.4955	8.3971	14.1	0.0003	0.0012	1045- BIT 150-1045 -Control
Unknown 1062	368.1058	4.1993	9.5	0.0017	0.0044	1045- BIT 150-1045 -Control
Unknown 1063	627.5385	7.5824	6.91	0.005887	0.011	1045- MIT 25-1045- BIT 150
Unknown 1064	522.2717	0.9125	6.4	0.0077865	0.0138	1045- BIT 150-1045 -Control
Unknown 1065	200.0836	1.0919	20.7	0	0.0004	1045- CIT 4-1045 -Control
Unknown 1068	354.1974	1.4785	29.4	0	0.0001	1045- BIT 150-1045 -Control
Unknown 1069	323.0911	0.9328	4.09	0.032535	0.0462	
Unknown 1071	244.1047	0.7833	8.83	0.0023055	0.0055	1045- CIT 4-1045 -Control
Unknown 1072	444.3464	8.0755	5.84	0.010679	0.0179	1045- BIT 150-1045 -Control
Unknown 1077	683.0552	0.6304	8.81	0.002329	0.0055	1045- BIT 150-1045 -Control
Unknown 1079	810.4911	8.3596	21	0	0.0003	1045- BIT 150-1045 -Control
Unknown 108	627.4206	6.6377	45.9	0	0	1045- BIT 150-1045 -Control
Unknown 1080	730.4988	8.2554	27.6	0	0.0001	1045- BIT 150-1045 -Control
Unknown 1081	658.5385	7.0456	9.18	0.002	0.0049	1045- BIT 150-1045 -Control
Unknown 1082	275.133	1.3558	12.6	0.0005	0.0018	1045- CIT 4-1045 -Control
Unknown 1083	353.229	5.3754	12.8	0.0005	0.0017	1045- BIT 150-1045 -Control
Unknown 1085	428.1879	0.8757	5.79	0.010959	0.0183	1045- BIT 150-1045 -Control
Unknown 1088	602.4171	7.9707	30.5	0	0.0001	1045- BIT 150-1045 -Control
Unknown 1089	644.3309	7.7076	11.8	0.0007	0.0022	1045- MIT 25-1045 -Control
Unknown 109	412.0566	0.7842	14	0.0003	0.0013	1045- BIT 150-1045 -Control
Unknown 1091	585.4914	6.7239	4.52	0.02425	0.0357	
Unknown 1092	849.4895	8.4959	144	0	0	1045- BIT 150-1045 -Control
Unknown 1094	534.2933	2.3583	7.26	0.0049158	0.0096	1045- CIT 4-1045 -Control
Unknown 1095	767.4884	8.4832	95.4	0	0	1045- BIT 150-1045 -Control

Unknown 1098	492.0878	0.8313	11.8	0.0007	0.0022	1045- BIT 150-1045 -Control
Unknown 1099	561.2529	6.766	55.5	0	0	1045- BIT 150-1045 -Control
Unknown 11	439.3041	6.3843	12.7	0.0005	0.0017	1045- BIT 150-1045 -Control
Unknown 1101	257.1746	3.3595	13.3	0.0004	0.0015	1045- BIT 150-1045 -Control
Unknown 1102	277.1834	5.9508	12	0.0006	0.0021	1045- BIT 150-1045 -Control
Unknown 1104	279.187	5.9201	7.55	0.0042484	0.0087	1045- CIT 4-1045- BIT 150
Unknown 1106	718.2859	7.3351	18.2	0.0001	0.0005	1045- BIT 150-1045 -Control
Unknown 1108	277.1221	3.0729	13.1	0.0004	0.0015	1045- CIT 4-1045 -Control
Unknown 1109	485.2659	1.122	4.95	0.018284	0.028	
Unknown 111	299.2587	5.9762	19.2	0.0001	0.0004	1045- BIT 150-1045 -Control
Unknown 1110	615.4056	8.245	19.2	0.0001	0.0004	1045- BIT 150-1045 -Control
Unknown 1111	277.1396	2.1998	8.7	0.0024434	0.0058	1045- BIT 150-1045 -Control
Unknown 1112	706.5408	7.151	9.39	0.0018	0.0045	1045- BIT 150-1045 -Control
Unknown 1114	552.3305	6.6117	38.3	0	0	1045- BIT 150-1045 -Control
Unknown 1118	152.0394	0.9727	7.69	0.0039528	0.0083	1045- CIT 4-1045- BIT 150
Unknown 1119	439.3009	6.4335	8.15	0.0031686	0.0071	1045- BIT 150-1045 -Control
Unknown 112	506.252	6.4455	27.1	0	0.0001	1045- BIT 150-1045 -Control
Unknown 1120	395.3247	7.3157	6.19	0.0087387	0.0152	1045- BIT 150-1045 -Control
Unknown 1121	127.1093	4.5761	8.48	0.0027016	0.0062	1045- BIT 150-1045 -Control
Unknown 1122	438.5993	0.594	48.2	0	0	1045- BIT 150-1045 -Control
Unknown 1123	201.1122	0.8378	14.3	0.0003	0.0012	1045- CIT 4-1045 -Control
Unknown 1124	730.5001	7.1056	24.6	0	0.0002	1045- BIT 150-1045 -Control
Unknown 1125	236.085	1.0311	7.57	0.0042023	0.0086	1045- CIT 4-1045 -Control
Unknown 1128	763.5148	7.9645	43.8	0	0	1045- BIT 150-1045 -Control
Unknown 113	411.2072	6.6484	11.5	0.0008	0.0024	1045- BIT 150-1045 -Control
Unknown 1130	164.07	1.1962	6.66	0.0067382	0.0123	1045- CIT 4-1045 -Control
Unknown 1133	425.2282	6.9823	7.51	0.0043271	0.0088	1045- MIT 25-1045- BIT 150
Unknown 1134	777.5172	7.5691	9.29	0.0019	0.0047	1045- BIT 150-1045 -Control
Unknown 1135	323.1894	6.4484	10.6	0.0011	0.0031	1045- CIT 4-1045- BIT 150
Unknown 1137	239.1499	3.8759	12.5	0.0005	0.0018	1045- BIT 150-1045 -Control
Unknown 1139	126.0204	5.12	7.57	0.0042047	0.0086	1045- CIT 4-1045- BIT 150
Unknown 1140	383.0543	0.6639	7.25	0.0049383	0.0096	1045- CIT 4-1045 -Control
Unknown 1143	443.1902	0.8403	6.19	0.0087341	0.0152	1045- CIT 4-1045 -Control
Unknown 1146	167.1366	3.1243	14.2	0.0003	0.0012	1045- BIT 150-1045 -Control
Unknown 1149	494.2936	6.871	7.74	0.0038569	0.0082	1045- CIT 4-1045- BIT 150
Unknown 1150	455.2389	5.8227	36.7	0	0.0001	1045- BIT 150-1045 -Control
Unknown 1151	702.4727	8.3862	53.1	0	0	1045- BIT 150-1045 -Control
Unknown 1153	257.1733	3.6642	16.1	0.0002	0.0008	1045- BIT 150-1045 -Control
Unknown 1154	382.2208	5.6331	7.32	0.0047697	0.0094	1045- BIT 150-1045 -Control
Unknown 1155	213.1124	3.5537	4.72	0.021213	0.0318	
Unknown 1157	311.2618	5.9893	19.5	0.0001	0.0004	1045- BIT 150-1045 -Control
Unknown 116	144.0606	0.6365	4.63	0.022539	0.0336	
Unknown 1162	496.0071	5.7297	7.97	0.0034473	0.0075	1045- BIT 150-1045 -Control
Unknown 1163	468.0108	6.3297	4.33	0.027613	0.0401	
Unknown 1164	435.2414	6.2484	10.2	0.0013	0.0035	1045- CIT 4-1045- BIT 150
Unknown 1165	253.2089	7.9725	8.61	0.0025534	0.0059	1045- CIT 4-1045 -Control
Unknown 1166	611.1866	0.835	7.75	0.0038346	0.0081	1045- BIT 150-1045 -Control
Unknown 1167	225.08	8.3117	4.82	0.019951	0.0303	

Unknown 1168	298.1681	0.816	5.37	0.014114	0.0227	
Unknown 1169	496.0064	8.3501	6.44	0.0076124	0.0136	1045- BIT 150-1045 -Control
Unknown 117	309.172	4.45	10.1	0.0013	0.0036	1045- CIT 4-1045- BIT 150
Unknown 1170	502.0437	0.7856	13.6	0.0004	0.0014	1045- BIT 150-1045 -Control
Unknown 1171	789.527	7.9828	39.1	0	0	1045- BIT 150-1045 -Control
Unknown 1172	655.244	0.6837	9.67	0.0016	0.0041	1045- MIT 25-1045 -Control
Unknown 1174	422.2894	6.8923	7.28	0.0048556	0.0095	1045- BIT 150-1045 -Control
Unknown 1175	522.371	8.9302	5.47	0.013322	0.0216	
Unknown 1176	249.1037	4.117	7.47	0.0044251	0.0089	1045- CIT 4-1045 -Control
Unknown 1178	364.174	0.841	6.49	0.0073749	0.0133	1045- MIT 25-1045- BIT 150
Unknown 1179	485.2387	6.6727	6.82	0.0061953	0.0115	1045- BIT 150-1045 -Control
Unknown 1180	641.554	7.5843	44.2	0	0	1045- BIT 150-1045 -Control
Unknown 1181	468.0466	0.8246	6.66	0.0067488	0.0123	1045- BIT 150-1045 -Control
Unknown 1184	209.0945	3.6413	18.2	0.0001	0.0005	1045- BIT 150-1045 -Control
Unknown 1186	701.4018	2.9693	11	0.0009	0.0027	1045- CIT 4-1045 -Control
Unknown 1187	330.2031	1.6645	10.2	0.0013	0.0034	1045- CIT 4-1045- BIT 150
Unknown 1188	717.4727	8.3116	20.3	0.0001	0.0004	1045- BIT 150-1045 -Control
Unknown 1189	437.2765	2.2355	18.6	0.0001	0.0005	1045- CIT 4-1045 -Control
Unknown 1190	361.1888	6.4912	6.15	0.0089179	0.0155	1045- BIT 150-1045 -Control
Unknown 1191	279.1636	5.8031	10.3	0.0012	0.0034	1045- CIT 4-1045 -Control
Unknown 1192	277.1235	2.1893	12.7	0.0005	0.0017	1045- BIT 150-1045 -Control
Unknown 1194	391.2051	1.4576	11.3	0.0008	0.0025	1045- CIT 4-1045 -Control
Unknown 1195	209.0645	3.6774	24	0	0.0002	1045- BIT 150-1045 -Control
Unknown 1196	505.1461	0.8163	13.1	0.0004	0.0016	1045- BIT 150-1045 -Control
Unknown 1197	201.1494	3.8284	5.28	0.014913	0.0238	
Unknown 1199	691.4635	8.273	13.9	0.0003	0.0013	1045- BIT 150-1045 -Control
Unknown 12	255.1244	3.2368	20.1	0.0001	0.0004	1045- BIT 150-1045 -Control
Unknown 120	481.2589	5.9797	43.6	0	0	1045- BIT 150-1045 -Control
Unknown 1200	447.1649	0.8169	10	0.0014	0.0037	1045- CIT 4-1045 -Control
Unknown 1201	629.5163	6.4142	20.6	0.0001	0.0004	1045- CIT 4-1045 -Control
Unknown 1205	808.4504	3.7088	11.5	0.0008	0.0024	1045- CIT 4-1045 -Control
Unknown 1206	584.049	0.7887	7.65	0.0040413	0.0084	1045- BIT 150-1045 -Control
Unknown 1207	601.3985	7.8866	10.4	0.0012	0.0033	1045- CIT 4-1045- BIT 150
Unknown 1208	458.1039	0.8135	8.47	0.0027174	0.0062	1045- BIT 150-1045 -Control
Unknown 1209	361.1806	3.337	14.9	0.0002	0.001	1045- BIT 150-1045 -Control
Unknown 121	441.3095	6.8304	4.97	0.018113	0.0278	
Unknown 1211	261.1178	1.2622	8.78	0.0023585	0.0056	1045- CIT 4-1045- BIT 150
Unknown 1212	425.3612	8.0797	7.59	0.0041634	0.0086	1045- BIT 150-1045 -Control
Unknown 1213	563.1622	0.8121	9.13	0.002	0.0049	1045- BIT 150-1045 -Control
Unknown 1214	496.0062	4.7011	5	0.017742	0.0273	
Unknown 1215	319.1299	1.4931	18.8	0.0001	0.0005	1045- BIT 150-1045 -Control
Unknown 1216	411.151	3.2338	7.15	0.0051917	0.01	1045- CIT 4-1045- BIT 150
Unknown 1219	223.1701	5.624	14.5	0.0003	0.0011	1045- BIT 150-1045 -Control
Unknown 1220	253.2152	7.1285	6.27	0.0083399	0.0147	1045- MIT 25-1045- BIT 150
Unknown 1223	529.4059	4.6607	8.37	0.0028532	0.0065	1045- CIT 4-1045- BIT 150
Unknown 1224	499.2973	8.6063	5.2	0.015634	0.0247	
Unknown 1226	774.4149	3.0703	5.17	0.015992	0.0251	
Unknown 1229	709.5554	4.3475	4.76	0.020748	0.0312	

Unknown 1230	468.0108	5.7241	4.75	0.020844	0.0313	
Unknown 1233	546.6099	0.5932	21.5	0	0.0003	1045- BIT 150-1045 -Control
Unknown 1235	562.3233	7.7597	31.2	0	0.0001	1045- BIT 150-1045 -Control
Unknown 1237	672.3133	1.1628	3.99	0.034938	0.0494	
Unknown 1238	651.5166	8.4972	11.2	0.0009	0.0025	1045- BIT 150-1045 -Control
Unknown 1239	213.0812	0.664	5.64	0.011972	0.0198	1045- CIT 4-1045 -Control
Unknown 124	188.0455	0.7936	18.9	0.0001	0.0005	1045- BIT 150-1045 -Control
Unknown 1240	408.3355	7.4822	4.22	0.029691	0.0427	
Unknown 1241	453.3688	7.8295	15.7	0.0002	0.0008	1045- BIT 150-1045 -Control
Unknown 1243	647.278	6.4686	25.1	0	0.0002	1045- BIT 150-1045 -Control
Unknown 1244	773.4609	8.2644	46.7	0	0	1045- BIT 150-1045 -Control
Unknown 1247	813.4914	8.5017	8.75	0.0023911	0.0056	1045- BIT 150-1045 -Control
Unknown 1248	297.0426	4.2315	5.52	0.012933	0.0211	
Unknown 125	396.1999	1.5565	31.5	0	0.0001	1045- BIT 150-1045 -Control
Unknown 1251	704.4844	8.2191	21.9	0	0.0003	1045- BIT 150-1045 -Control
Unknown 1252	481.3301	7.7587	5.1	0.016706	0.0261	
Unknown 1253	231.1599	3.2826	15.5	0.0002	0.0009	1045- BIT 150-1045 -Control
Unknown 1255	503.2462	3.6679	18.2	0.0001	0.0005	1045- BIT 150-1045 -Control
Unknown 1257	488.195	0.6439	8.87	0.0023	0.0054	1045- CIT 4-1045 -Control
Unknown 1258	657.3261	1.5308	18.8	0.0001	0.0005	1045- BIT 150-1045 -Control
Unknown 1259	397.2443	1.3056	22.3	0	0.0003	1045- BIT 150-1045 -Control
Unknown 126	307.1882	5.908	29.7	0	0.0001	1045- BIT 150-1045 -Control
Unknown 1260	595.3187	7.0479	12	0.0006	0.0021	1045- BIT 150-1045 -Control
Unknown 1261	598.0606	0.7991	14.3	0.0003	0.0012	1045- BIT 150-1045 -Control
Unknown 1262	630.2602	0.848	6.11	0.0091538	0.0157	1045- CIT 4-1045 -Control
Unknown 1263	427.3428	7.7698	20	0.0001	0.0004	1045- BIT 150-1045 -Control
Unknown 1264	692.3918	2.5076	8.6	0.0025545	0.0059	1045- BIT 150-1045 -Control
Unknown 1266	158.0696	0.8648	6.64	0.0068077	0.0123	1045- CIT 4-1045 -Control
Unknown 1268	514.2574	0.8509	10.2	0.0013	0.0034	1045- CIT 4-1045 -Control
Unknown 1269	448.2185	1.0269	6.79	0.0062931	0.0117	1045- CIT 4-1045 -Control
Unknown 127	417.2222	6.6555	9.6	0.0016	0.0042	1045- BIT 150-1045 -Control
Unknown 1271	390.1269	1.9062	17.2	0.0001	0.0006	1045- BIT 150-1045 -Control
Unknown 1272	639.5057	5.5245	7.75	0.0038439	0.0082	1045- BIT 150-1045 -Control
Unknown 1274	633.4545	8.5067	42	0	0	1045- BIT 150-1045 -Control
Unknown 1276	655.3952	7.891	8.78	0.0023526	0.0056	1045- CIT 4-1045- BIT 150
Unknown 1278	426.7093	0.8503	10.3	0.0012	0.0034	1045- CIT 4-1045 -Control
Unknown 1279	655.3812	2.8376	7.44	0.0044875	0.009	1045- CIT 4-1045 -Control
Unknown 128	174.0315	0.7625	5.71	0.011512	0.0191	1045- CIT 4-1045- BIT 150
Unknown 1280	620.3093	7.3499	27.2	0	0.0001	1045- BIT 150-1045 -Control
Unknown 1281	655.3759	7.8938	8.5	0.0026809	0.0062	1045- CIT 4-1045- BIT 150
Unknown 1283	227.0284	3.4101	4.99	0.017884	0.0275	
Unknown 1284	700.5907	7.0587	8.79	0.0023451	0.0056	1045- BIT 150-1045 -Control
Unknown 1285	789.6764	7.1406	7.29	0.0048438	0.0095	1045- BIT 150-1045 -Control
Unknown 1287	496.4112	7.4503	6.91	0.0058845	0.011	1045- BIT 150-1045 -Control
Unknown 1289	281.2483	7.7374	9.85	0.0015	0.0039	1045- MIT 25-1045- BIT 150
Unknown 1291	496.0059	4.4159	6.23	0.0085419	0.015	1045- MIT 25-1045- BIT 150
Unknown 1293	496.0062	5.0294	6.37	0.0078976	0.014	1045- BIT 150-1045 -Control
Unknown 1294	576.294	6.7994	9.71	0.0016	0.0041	1045- CIT 4-1045- BIT 150

Unknown 1295	199.1327	2.6244	6.21	0.0086278	0.0151	1045- CIT 4-1045- BIT 150
Unknown 1296	397.3449	7.4837	9.15	0.002	0.0049	1045- BIT 150-1045 -Control
Unknown 1298	599.5069	7.0337	13.9	0.0003	0.0013	1045- BIT 150-1045 -Control
Unknown 1299	338.2675	6.0834	8.12	0.0032013	0.0071	1045- BIT 150-1045 -Control
Unknown 13	147.0522	0.8895	7.37	0.0046543	0.0092	1045- CIT 4-1045 -Control
Unknown 130	349.2694	7.5684	5.67	0.011793	0.0195	1045- MIT 25-1045- BIT 150
Unknown 1300	551.2623	6.4565	26.9	0	0.0001	1045- BIT 150-1045 -Control
Unknown 1301	235.1182	1.2297	6.75	0.0064168	0.0118	1045- CIT 4-1045 -Control
Unknown 1303	582.3492	0.9642	5.84	0.010695	0.0179	1045- CIT 4-1045 -Control
Unknown 1304	704.4888	8.7035	6.44	0.0076027	0.0136	1045- CIT 4-1045- BIT 150
Unknown 1307	165.1236	3.2296	7.3	0.0048138	0.0095	1045- CIT 4-1045- BIT 150
Unknown 1308	433.2334	6.7876	4.16	0.030919	0.0442	
Unknown 131	805.6227	6.7439	55.6	0	0	1045- BIT 150-1045 -Control
Unknown 1310	466.3087	7.0352	26.6	0	0.0002	1045- BIT 150-1045 -Control
Unknown 1311	247.0995	3.3976	35.7	0	0.0001	1045- BIT 150-1045 -Control
Unknown 1312	803.3829	2.1643	18.2	0.0001	0.0005	1045- BIT 150-1045 -Control
Unknown 1314	459.1973	6.4576	10.5	0.0011	0.0031	1045- BIT 150-1045 -Control
Unknown 1315	513.321	7.6436	5	0.017741	0.0273	
Unknown 1316	842.599	8.4465	17.8	0.0001	0.0006	1045- BIT 150-1045 -Control
Unknown 1317	202.999	3.0708	4.26	0.028826	0.0416	
Unknown 1318	516.062	0.8045	13	0.0004	0.0016	1045- BIT 150-1045 -Control
Unknown 1319	687.5737	7.6645	4.19	0.03029	0.0434	
Unknown 132	197.1545	5.1221	47.3	0	0	1045- BIT 150-1045 -Control
Unknown 1320	704.4848	8.2856	18.2	0.0001	0.0005	1045- BIT 150-1045 -Control
Unknown 1321	253.2108	8.8142	11.5	0.0008	0.0023	1045- CIT 4-1045- BIT 150
Unknown 1322	585.4914	6.6475	11.3	0.0008	0.0024	1045- BIT 150-1045 -Control
Unknown 1324	754.5533	5.5284	9.42	0.0018	0.0045	1045- BIT 150-1045 -Control
Unknown 1326	277.1225	8.228	11.4	0.0008	0.0024	1045- CIT 4-1045- BIT 150
Unknown 1329	691.5447	5.4799	5.2	0.01571	0.0248	
Unknown 1330	579.7942	2.8384	9.07	0.0021	0.005	1045- CIT 4-1045- BIT 150
Unknown 1333	810.4913	8.2977	30.1	0	0.0001	1045- BIT 150-1045 -Control
Unknown 1336	176.0179	0.7631	6.55	0.0071551	0.0129	1045- CIT 4-1045 -Control
Unknown 1337	685.3642	2.1684	23.7	0	0.0002	1045- BIT 150-1045 -Control
Unknown 1338	742.5042	8.5139	7.66	0.0040201	0.0084	1045- CIT 4-1045- BIT 150
Unknown 1339	240.1218	1.0108	7.86	0.0036302	0.0078	1045- CIT 4-1045 -Control
Unknown 134	277.0943	1.043	27.5	0	0.0001	1045- BIT 150-1045 -Control
Unknown 1340	511.3044	6.9382	20.3	0.0001	0.0004	1045- BIT 150-1045 -Control
Unknown 1341	329.0736	3.3149	15.1	0.0002	0.001	1045- BIT 150-1045 -Control
Unknown 1342	433.2272	6.7486	9.05	0.0021	0.0051	1045- BIT 150-1045 -Control
Unknown 1343	812.5082	7.0941	19	0.0001	0.0005	1045- CIT 4-1045 -Control
Unknown 1344	157.1232	3.8498	5.27	0.015006	0.0239	
Unknown 1345	843.5588	8.8115	6.75	0.006411	0.0118	1045- CIT 4-1045 -Control
Unknown 1346	641.1867	0.8804	17.2	0.0001	0.0006	1045- BIT 150-1045 -Control
Unknown 1347	738.3487	2.4159	13.1	0.0004	0.0015	1045- CIT 4-1045 -Control
Unknown 1348	154.059	0.8559	5.67	0.011817	0.0195	1045- CIT 4-1045 -Control
Unknown 1349	488.2588	1.1948	7.29	0.0048391	0.0095	1045- CIT 4-1045 -Control
Unknown 1350	219.9729	0.6453	4.77	0.020607	0.031	
Unknown 1353	281.2483	7.9345	30.7	0	0.0001	1045- CIT 4-1045 -Control

Unknown 1354	577.2778	6.615	21.3	0	0.0003	1045- BIT 150-1045 -Control
Unknown 1355	130.0875	2.0831	5.2	0.015648	0.0247	
Unknown 1356	553.2329	1.0087	13.1	0.0004	0.0016	1045- CIT 4-1045- BIT 150
Unknown 1357	474.265	7.0495	7.48	0.0043842	0.0089	1045- BIT 150-1045 -Control
Unknown 1358	471.2412	6.7076	4.91	0.018758	0.0286	
Unknown 1359	471.2419	6.7173	4.44	0.025569	0.0374	
Unknown 1360	169.0851	0.9341	5.24	0.015332	0.0243	
Unknown 1364	687.4187	3.2049	10.8	0.001	0.0029	1045- CIT 4-1045 -Control
Unknown 1365	433.1419	3.2392	4.77	0.020532	0.031	
Unknown 1366	583.2493	0.9197	12.1	0.0006	0.002	1045- CIT 4-1045 -Control
Unknown 1369	595.666	0.6001	50.7	0	0	1045- BIT 150-1045 -Control
Unknown 137	424.3419	8.3116	8.26	0.0029978	0.0068	1045- CIT 4-1045- BIT 150
Unknown 1370	245.043	8.4981	27.8	0	0.0001	1045- BIT 150-1045 -Control
Unknown 1373	287.1877	3.6675	3.97	0.035412	0.0499	
Unknown 1374	335.2095	4.4333	11.7	0.0007	0.0022	1045- MIT 25-1045 -Control
Unknown 1375	672.5542	7.1481	16.2	0.0002	0.0008	1045- BIT 150-1045 -Control
Unknown 1376	832.5688	8.2816	27	0	0.0001	1045- BIT 150-1045 -Control
Unknown 1379	291.1908	5.1791	8.54	0.0026348	0.0061	1045- CIT 4-1045- BIT 150
Unknown 138	532.3096	7.0538	7.25	0.004949	0.0096	1045- BIT 150-1045 -Control
Unknown 1382	286.1707	0.9568	7.54	0.0042658	0.0087	1045- CIT 4-1045 -Control
Unknown 1384	506.9571	5.0862	5.37	0.014134	0.0227	
Unknown 1385	474.1764	0.6344	5.74	0.01132	0.0189	1045- CIT 4-1045- BIT 150
Unknown 1386	758.533	8.468	34.3	0	0.0001	1045- BIT 150-1045 -Control
Unknown 1389	680.0605	0.7835	10.4	0.0012	0.0033	1045- BIT 150-1045 -Control
Unknown 139	283.1573	0.807	6.25	0.0084595	0.0148	1045- CIT 4-1045 -Control
Unknown 1391	489.2314	0.9795	8.14	0.0031723	0.0071	1045- CIT 4-1045 -Control
Unknown 1392	574.0873	0.8203	4.11	0.031989	0.0455	
Unknown 1394	621.1851	0.6183	7.61	0.0041235	0.0085	1045- CIT 4-1045 -Control
Unknown 1395	425.2729	2.0904	44.4	0	0	1045- BIT 150-1045 -Control
Unknown 1398	551.5038	7.3681	6.31	0.0081647	0.0144	1045- MIT 25-1045- BIT 150
Unknown 1399	529.1674	0.8098	11	0.0009	0.0027	1045- BIT 150-1045 -Control
Unknown 14	483.2748	6.5907	26.8	0	0.0001	1045- BIT 150-1045 -Control
Unknown 140	368.3144	7.0579	7.98	0.0034278	0.0075	1045- BIT 150-1045 -Control
Unknown 1400	599.2284	0.926	7.02	0.0055708	0.0106	1045- CIT 4-1045 -Control
Unknown 1401	756.5179	8.5399	10.6	0.0011	0.003	1045- BIT 150-1045 -Control
Unknown 1403	245.043	8.5559	41.3	0	0	1045- BIT 150-1045 -Control
Unknown 1405	348.1924	2.0974	21.7	0	0.0003	1045- BIT 150-1045 -Control
Unknown 1406	454.3567	7.7223	24.1	0	0.0002	1045- BIT 150-1045 -Control
Unknown 1407	149.0751	1.0044	4.56	0.02358	0.035	
Unknown 1408	361.1209	3.2777	12.1	0.0006	0.002	1045- BIT 150-1045 -Control
Unknown 1409	601.3882	8.1594	7.76	0.0038242	0.0081	1045- CIT 4-1045- BIT 150
Unknown 141	478.2927	7.334	13.6	0.0004	0.0013	1045- BIT 150-1045 -Control
Unknown 1410	629.5163	5.8507	18	0.0001	0.0005	1045- BIT 150-1045 -Control
Unknown 1411	321.1211	0.9635	24.8	0	0.0002	1045- BIT 150-1045 -Control
Unknown 1413	430.2452	0.7706	5.99	0.0097683	0.0166	1045- CIT 4-1045 -Control
Unknown 1414	130.0872	1.822	13.3	0.0004	0.0015	1045- BIT 150-1045 -Control
Unknown 1416	729.442	2.3001	11.7	0.0007	0.0022	1045- BIT 150-1045 -Control
Unknown 1417	534.252	1.0333	8.58	0.0025836	0.006	1045- CIT 4-1045 -Control

Unknown 1418	401.2933	7.6761	25.3	0	0.0002	1045- BIT 150-1045 -Control
Unknown 1419	613.5206	7.0448	9.34	0.0018	0.0046	1045- BIT 150-1045 -Control
Unknown 1421	253.2074	8.3616	17.7	0.0001	0.0006	1045- BIT 150-1045 -Control
Unknown 1422	433.3464	8.8024	10.5	0.0011	0.0031	1045- BIT 150-1045 -Control
Unknown 1423	444.2357	0.9421	7.98	0.0034358	0.0075	1045- CIT 4-1045 -Control
Unknown 1426	253.2089	8.4837	13.3	0.0004	0.0015	1045- BIT 150-1045 -Control
Unknown 1427	328.1904	1.126	9.97	0.0014	0.0037	1045- CIT 4-1045 -Control
Unknown 1428	252.9697	3.7603	7.34	0.0047074	0.0093	1045- CIT 4-1045- BIT 150
Unknown 1429	827.5072	8.5013	10.6	0.0011	0.003	1045- BIT 150-1045 -Control
Unknown 143	185.1181	2.6855	7.37	0.0046457	0.0092	1045- BIT 150-1045 -Control
Unknown 1430	614.2716	6.7033	9.1	0.002	0.005	1045- BIT 150-1045 -Control
Unknown 1431	787.5115	8.0285	22.3	0	0.0003	1045- BIT 150-1045 -Control
Unknown 1432	688.3291	1.3228	14.8	0.0002	0.001	1045- BIT 150-1045 -Control
Unknown 1433	616.9146	0.5884	15.3	0.0002	0.0009	1045- BIT 150-1045 -Control
Unknown 1434	614.2723	6.6593	8.94	0.0022	0.0053	1045- BIT 150-1045 -Control
Unknown 1435	669.2599	2.6894	4.61	0.022837	0.034	
Unknown 1437	789.5274	8.3954	19.9	0.0001	0.0004	1045- CIT 4-1045 -Control
Unknown 1438	199.1701	8.3266	5	0.017736	0.0273	
Unknown 144	251.2014	6.2826	44	0	0	1045- BIT 150-1045 -Control
Unknown 1441	618.2432	1.484	38.9	0	0	1045- BIT 150-1045 -Control
Unknown 1442	387.2207	1.0754	7.39	0.0045908	0.0091	1045- CIT 4-1045 -Control
Unknown 1443	462.3196	7.1544	12.9	0.0005	0.0016	1045- BIT 150-1045 -Control
Unknown 1444	254.127	3.692	10.9	0.001	0.0028	1045- MIT 25-1045 -Control
Unknown 1449	342.1897	2.7935	5.86	0.010549	0.0177	1045- MIT 25-1045- BIT 150
Unknown 1450	114.9945	0.6438	5.49	0.013106	0.0213	
Unknown 1451	143.0204	2.1594	13.7	0.0003	0.0013	1045- BIT 150-1045 -Control
Unknown 1452	559.4754	6.4512	23.3	0	0.0002	1045- BIT 150-1045 -Control
Unknown 1453	692.2851	2.695	6.4	0.0077511	0.0138	1045- CIT 4-1045 -Control
Unknown 1454	745.5016	8.5058	13.7	0.0003	0.0013	1045- BIT 150-1045 -Control
Unknown 1455	410.1778	0.9302	13.9	0.0003	0.0013	1045- CIT 4-1045 -Control
Unknown 1459	563.2598	8.4956	46.9	0	0	1045- BIT 150-1045 -Control
Unknown 1460	340.1747	1.0839	4.77	0.02053	0.031	
Unknown 1461	591.291	8.5115	26.9	0	0.0001	1045- BIT 150-1045 -Control
Unknown 1462	271.1383	1.1749	5.8	0.010915	0.0183	1045- CIT 4-1045 -Control
Unknown 1463	590.3998	8.4078	34.6	0	0.0001	1045- BIT 150-1045 -Control
Unknown 1464	253.2093	8.0701	12.6	0.0005	0.0018	1045- MIT 25-1045 -Control
Unknown 1465	834.5886	8.4653	14.5	0.0003	0.0011	1045- BIT 150-1045 -Control
Unknown 1466	573.4912	6.4514	42.9	0	0	1045- BIT 150-1045 -Control
Unknown 1468	732.5128	8.4359	13.4	0.0004	0.0014	1045- BIT 150-1045 -Control
Unknown 147	395.2269	5.66	5.26	0.015062	0.024	
Unknown 1470	130.0868	1.1284	4.37	0.026853	0.0391	
Unknown 1471	655.5692	7.7355	13.7	0.0003	0.0013	1045- BIT 150-1045 -Control
Unknown 1473	645.446	1.8187	26.5	0	0.0002	1045- BIT 150-1045 -Control
Unknown 1474	431.3117	7.6556	4.43	0.025685	0.0375	
Unknown 1475	495.1986	0.9745	5.97	0.0098975	0.0168	1045- CIT 4-1045- BIT 150
Unknown 1476	551.3151	1.6342	7.46	0.0044355	0.0089	1045- CIT 4-1045 -Control
Unknown 1477	374.207	2.3463	13.7	0.0003	0.0013	1045- CIT 4-1045 -Control
Unknown 1479	763.505	7.6731	22.3	0	0.0003	1045- BIT 150-1045 -Control

Unknown 148	428.3166	7.138	4.85	0.019489	0.0296	
Unknown 1480	449.1509	0.9337	6.1	0.0091734	0.0157	1045- CIT 4-1045 -Control
Unknown 1481	197.0744	3.1449	7.01	0.0055833	0.0106	1045- CIT 4-1045- BIT 150
Unknown 1484	402.1182	1.2692	22.2	0	0.0003	1045- BIT 150-1045 -Control
Unknown 1486	363.2226	6.7429	6.14	0.0089648	0.0155	1045- CIT 4-1045- BIT 150
Unknown 1487	623.3399	7.5174	8.07	0.0032885	0.0073	1045- CIT 4-1045- BIT 150
Unknown 1488	803.383	2.1386	15.7	0.0002	0.0008	1045- BIT 150-1045 -Control
Unknown 1489	306.0524	0.6791	8.06	0.0032972	0.0073	1045- CIT 4-1045 -Control
Unknown 149	425.3661	7.5402	22.8	0	0.0003	1045- BIT 150-1045 -Control
Unknown 1491	650.3978	7.5264	31.9	0	0.0001	1045- BIT 150-1045 -Control
Unknown 1492	632.2947	2.4174	16.6	0.0001	0.0007	1045- CIT 4-1045 -Control
Unknown 1494	277.1286	1.58	27	0	0.0001	1045- BIT 150-1045 -Control
Unknown 1496	780.4229	2.0203	7.55	0.0042421	0.0087	1045- CIT 4-1045- BIT 150
Unknown 1497	696.3119	2.0357	56.4	0	0	1045- BIT 150-1045 -Control
Unknown 1498	619.3194	6.9835	14.1	0.0003	0.0012	1045- CIT 4-1045 -Control
Unknown 1499	614.2681	7.3394	7.8	0.0037532	0.008	1045- CIT 4-1045- BIT 150
Unknown 15	805.63	6.6361	80.7	0	0	1045- BIT 150-1045 -Control
Unknown 150	279.2045	5.6876	6.84	0.0061029	0.0114	1045- MIT 25-1045- BIT 150
Unknown 1500	367.2043	4.9667	13.5	0.0004	0.0014	1045- MIT 25-1045 -Control
Unknown 1502	631.4535	8.2453	7.32	0.0047602	0.0094	1045- MIT 25-1045 -Control
Unknown 1504	185.1181	2.0228	11.4	0.0008	0.0024	1045- BIT 150-1045 -Control
Unknown 1506	685.3424	2.1359	27.6	0	0.0001	1045- BIT 150-1045 -Control
Unknown 1507	795.4588	2.0991	34	0	0.0001	1045- BIT 150-1045 -Control
Unknown 1508	549.1986	6.4492	6.52	0.0072842	0.0131	1045- CIT 4-1045- BIT 150
Unknown 1509	241.1016	1.793	14.3	0.0003	0.0012	1045- BIT 150-1045 -Control
Unknown 151	386.0373	0.8574	7.96	0.0034684	0.0075	1045- BIT 150-1045 -Control
Unknown 1510	679.252	0.6673	5.08	0.016892	0.0263	
Unknown 1511	344.2187	1.5328	37.1	0	0.0001	1045- BIT 150-1045 -Control
Unknown 1512	535.6167	0.5587	32	0	0.0001	1045- BIT 150-1045 -Control
Unknown 1515	367.3066	5.9241	5.02	0.017533	0.0272	
Unknown 1516	471.262	2.4222	4.44	0.025575	0.0374	
Unknown 1519	682.377	1.5301	36.8	0	0.0001	1045- BIT 150-1045 -Control
Unknown 1520	794.5352	8.9979	12.7	0.0005	0.0017	1045- CIT 4-1045 -Control
Unknown 1521	226.1146	0.9028	10.9	0.001	0.0028	1045- CIT 4-1045 -Control
Unknown 1522	845.2207	4.7835	10.7	0.001	0.003	1045- CIT 4-1045- BIT 150
Unknown 1523	712.4915	9.0299	4.42	0.025984	0.0379	
Unknown 1526	415.308	7.6935	15.1	0.0002	0.001	1045- BIT 150-1045 -Control
Unknown 1528	399.2558	2.2401	20.2	0.0001	0.0004	1045- CIT 4-1045 -Control
Unknown 153	452.2792	7.0964	22.1	0	0.0003	1045- BIT 150-1045 -Control
Unknown 1531	543.6028	0.5712	47.9	0	0	1045- BIT 150-1045 -Control
Unknown 1532	446.1368	0.6607	7.28	0.0048684	0.0095	1045- CIT 4-1045 -Control
Unknown 1533	750.5352	7.189	8.62	0.0025317	0.0059	1045- BIT 150-1045 -Control
Unknown 1534	297.079	0.6503	10.3	0.0012	0.0034	1045- MIT 25-1045- BIT 150
Unknown 1535	519.2796	2.0452	7.71	0.0039193	0.0083	1045- CIT 4-1045- BIT 150
Unknown 1536	305.2145	6.3705	12.9	0.0005	0.0016	1045- BIT 150-1045 -Control
Unknown 1537	496.3001	7.1669	5.1	0.016683	0.0261	
Unknown 1539	723.4932	8.9554	18.6	0.0001	0.0005	1045- BIT 150-1045 -Control
Unknown 1541	447.2424	7.0694	4.14	0.031325	0.0447	

Unknown 1542	323.2124	5.77	7.22	0.0050261	0.0097	1045- CIT 4-1045- BIT 150
Unknown 1543	332.6699	1.3286	12.6	0.0005	0.0018	1045- CIT 4-1045- BIT 150
Unknown 1544	679.3076	1.5122	8.68	0.0024728	0.0058	1045- CIT 4-1045- BIT 150
Unknown 1546	209.1279	3.5895	12.8	0.0005	0.0017	1045- BIT 150-1045 -Control
Unknown 1547	648.4393	8.4689	38.8	0	0	1045- BIT 150-1045 -Control
Unknown 1548	315.2536	5.1209	11.2	0.0009	0.0026	1045- CIT 4-1045- BIT 150
Unknown 1549	428.1855	2.0304	17.7	0.0001	0.0006	1045- BIT 150-1045 -Control
Unknown 155	650.3403	0.8781	7.26	0.0049117	0.0096	1045- CIT 4-1045 -Control
Unknown 1553	381.2194	4.4338	4.63	0.022531	0.0336	
Unknown 1555	425.2392	1.2997	20.6	0.0001	0.0004	1045- BIT 150-1045 -Control
Unknown 1558	245.0951	0.6567	6.26	0.0083841	0.0147	1045- CIT 4-1045 -Control
Unknown 1559	235.1177	1.7335	8.99	0.0021	0.0052	1045- CIT 4-1045- BIT 150
Unknown 156	313.2355	3.9328	8.47	0.0027131	0.0062	1045- BIT 150-1045 -Control
Unknown 1560	263.0065	0.8581	7.66	0.0040111	0.0084	1045- CIT 4-1045 -Control
Unknown 1561	489.2709	1.8381	36.1	0	0.0001	1045- BIT 150-1045 -Control
Unknown 1562	555.3279	1.0788	9.19	0.002	0.0048	1045- CIT 4-1045 -Control
Unknown 1564	559.3421	7.5161	13.1	0.0004	0.0016	1045- BIT 150-1045 -Control
Unknown 1566	560.2757	1.4649	24.4	0	0.0002	1045- BIT 150-1045 -Control
Unknown 1567	340.2238	1.9017	11.4	0.0008	0.0024	1045- CIT 4-1045- BIT 150
Unknown 1568	227.1283	0.9606	5.31	0.01466	0.0235	
Unknown 1569	459.2254	1.4026	21.1	0	0.0003	1045- BIT 150-1045 -Control
Unknown 157	735.4669	6.9414	42.8	0	0	1045- BIT 150-1045 -Control
Unknown 1571	754.5569	7.1431	8.08	0.0032772	0.0073	1045- BIT 150-1045 -Control
Unknown 1573	616.2841	7.0919	23.3	0	0.0002	1045- BIT 150-1045 -Control
Unknown 1574	452.2966	7.4996	5.07	0.017032	0.0265	
Unknown 1575	319.2307	6.8439	11.3	0.0008	0.0025	1045- BIT 150-1045 -Control
Unknown 1576	266.134	3.417	19.7	0.0001	0.0004	1045- BIT 150-1045 -Control
Unknown 1577	582.3567	1.5113	8.74	0.002397	0.0057	1045- CIT 4-1045- BIT 150
Unknown 1578	679.3083	1.573	13.9	0.0003	0.0013	1045- BIT 150-1045 -Control
Unknown 1579	261.1185	1.2863	6.77	0.0063491	0.0118	1045- CIT 4-1045- BIT 150
Unknown 158	473.2063	1.0374	11.6	0.0007	0.0023	1045- CIT 4-1045 -Control
Unknown 1580	520.2654	7.0973	23.9	0	0.0002	1045- BIT 150-1045 -Control
Unknown 1581	645.1627	0.8057	6.35	0.0079724	0.0141	1045- BIT 150-1045 -Control
Unknown 1582	342.2393	1.9978	59.5	0	0	1045- BIT 150-1045 -Control
Unknown 1583	565.2804	6.0596	4.6	0.022977	0.0342	
Unknown 1584	496.0061	6.0758	7.43	0.0044953	0.009	1045- BIT 150-1045 -Control
Unknown 1585	133.0764	2.7803	4.53	0.024126	0.0355	
Unknown 1586	482.2977	2.0178	20.3	0.0001	0.0004	1045- BIT 150-1045 -Control
Unknown 1587	613.5196	7.0104	4.1	0.032365	0.046	
Unknown 1588	383.2294	1.3439	33.1	0	0.0001	1045- BIT 150-1045 -Control
Unknown 1589	119.0363	1.4601	4.22	0.029722	0.0427	
Unknown 159	724.3018	7.3482	9.43	0.0018	0.0045	1045- BIT 150-1045 -Control
Unknown 1590	382.1938	2.0511	22.9	0	0.0003	1045- BIT 150-1045 -Control
Unknown 1591	731.5068	8.7544	7.03	0.005522	0.0105	1045- CIT 4-1045- BIT 150
Unknown 1592	243.1631	1.0364	14.8	0.0002	0.001	1045- CIT 4-1045 -Control
Unknown 1593	496.3127	2.3136	9.77	0.0015	0.004	1045- CIT 4-1045 -Control
Unknown 1594	729.6246	7.6661	7.52	0.0043052	0.0088	1045- BIT 150-1045 -Control
Unknown 1595	284.0473	0.9063	5.01	0.017618	0.0273	

Unknown 1596	513.2417	7.0366	9.41	0.0018	0.0045	1045- BIT 150-1045 -Control
Unknown 16	529.4204	6.6419	40.7	0	0	1045- BIT 150-1045 -Control
Unknown 162	385.1433	0.8244	4.5	0.024498	0.036	
Unknown 163	576.2853	7.339	18.2	0.0001	0.0005	1045- BIT 150-1045 -Control
Unknown 164	335.2201	6.7213	12.4	0.0005	0.0018	1045- MIT 25-1045 -Control
Unknown 166	264.1076	2.7627	6.47	0.0074671	0.0134	1045- BIT 150-1045 -Control
Unknown 167	756.5158	7.2387	23.8	0	0.0002	1045- BIT 150-1045 -Control
Unknown 168	335.2202	7.0358	13.8	0.0003	0.0013	1045- BIT 150-1045 -Control
Unknown 169	233.0967	0.8028	7.04	0.0055059	0.0105	1045- CIT 4-1045 -Control
Unknown 17	377.2402	5.5527	13.7	0.0003	0.0013	1045- BIT 150-1045 -Control
Unknown 171	321.2062	6.7391	9.3	0.0019	0.0047	1045- MIT 25-1045 -Control
Unknown 172	241.1808	4.1449	16.1	0.0002	0.0008	1045- BIT 150-1045 -Control
Unknown 173	464.2849	7.0292	15	0.0002	0.001	1045- BIT 150-1045 -Control
Unknown 174	379.2574	5.5993	11.4	0.0008	0.0024	1045- BIT 150-1045 -Control
Unknown 177	383.1413	2.7755	24.4	0	0.0002	1045- BIT 150-1045 -Control
Unknown 178	695.5202	7.5834	8.3	0.002948	0.0067	1045- MIT 25-1045- BIT 150
Unknown 179	475.1852	0.7526	6.96	0.0057529	0.0108	1045- CIT 4-1045 -Control
Unknown 18	328.0747	0.8998	29.1	0	0.0001	1045- BIT 150-1045 -Control
Unknown 181	409.3206	7.1032	7.56	0.0042126	0.0086	1045- BIT 150-1045 -Control
Unknown 182	461.2525	5.8454	12.5	0.0005	0.0018	1045- BIT 150-1045 -Control
Unknown 184	313.2354	3.9135	7.48	0.0043866	0.0089	1045- CIT 4-1045- BIT 150
Unknown 186	307.1885	5.9753	70.6	0	0	1045- BIT 150-1045 -Control
Unknown 187	361.2358	6.8968	11.1	0.0009	0.0027	1045- MIT 25-1045 -Control
Unknown 188	351.2207	7.1259	8.68	0.0024661	0.0058	1045- MIT 25-1045 -Control
Unknown 19	361.1881	0.8236	12.9	0.0005	0.0016	1045- BIT 150-1045 -Control
Unknown 191	389.1973	5.8741	12	0.0006	0.002	1045- BIT 150-1045 -Control
Unknown 192	605.3099	7.016	33	0	0.0001	1045- BIT 150-1045 -Control
Unknown 193	573.4905	6.8164	4.55	0.023749	0.0352	
Unknown 194	189.0176	0.7738	12.6	0.0005	0.0018	1045- BIT 150-1045 -Control
Unknown 195	296.188	5.1334	43.9	0	0	1045- BIT 150-1045 -Control
Unknown 196	509.2883	6.5859	21	0	0.0003	1045- BIT 150-1045 -Control
Unknown 197	670.3565	6.9523	12.7	0.0005	0.0017	1045- BIT 150-1045 -Control
Unknown 198	256.1045	0.7643	6.54	0.0071938	0.013	1045- CIT 4-1045- BIT 150
Unknown 199	337.2006	4.9465	11.9	0.0007	0.0021	1045- BIT 150-1045 -Control
Unknown 2	297.1681	4.4977	15.6	0.0002	0.0009	1045- BIT 150-1045 -Control
Unknown 20	353.2264	6.2334	10.6	0.0011	0.0031	1045- MIT 25-1045 -Control
Unknown 200	663.4168	8.0376	8.44	0.0027635	0.0063	1045- CIT 4-1045- BIT 150
Unknown 201	409.1915	0.9914	11.1	0.0009	0.0027	1045- CIT 4-1045 -Control
Unknown 202	281.2108	6.4509	20.2	0.0001	0.0004	1045- MIT 25-1045 -Control
Unknown 203	691.545	5.5144	18.1	0.0001	0.0005	1045- BIT 150-1045 -Control
Unknown 205	295.1893	6.4494	21.9	0	0.0003	1045- BIT 150-1045 -Control
Unknown 206	467.3252	6.9222	9.09	0.0021	0.005	1045- BIT 150-1045 -Control
Unknown 207	211.1702	5.573	43.5	0	0	1045- BIT 150-1045 -Control
Unknown 208	547.0755	0.6507	17.9	0.0001	0.0005	1045- BIT 150-1045 -Control
Unknown 21	563.26	5.973	40.6	0	0	1045- BIT 150-1045 -Control
Unknown 210	503.2309	6.0394	5.48	0.013195	0.0214	
Unknown 211	309.2045	6.4488	16.9	0.0001	0.0007	1045- MIT 25-1045 -Control
Unknown 214	328.195	1.4398	23.9	0	0.0002	1045- BIT 150-1045 -Control

Unknown 215	281.1358	4.0087	4.36	0.026996	0.0392	
Unknown 216	291.1183	3.2502	8.58	0.0025795	0.006	1045- CIT 4-1045- BIT 150
Unknown 218	116.0569	0.8987	11.9	0.0007	0.0021	1045- CIT 4-1045 -Control
Unknown 22	450.2713	6.5982	30	0	0.0001	1045- BIT 150-1045 -Control
Unknown 220	376.1806	1.1669	7.97	0.0034403	0.0075	1045- CIT 4-1045 -Control
Unknown 221	606.2319	0.8945	8.84	0.0023	0.0055	1045- CIT 4-1045 -Control
Unknown 222	761.4945	7.9693	19.6	0.0001	0.0004	1045- BIT 150-1045 -Control
Unknown 226	261.0594	0.9579	7.31	0.0047965	0.0094	1045- CIT 4-1045- BIT 150
Unknown 227	267.2327	7.0372	18.6	0.0001	0.0005	1045- BIT 150-1045 -Control
Unknown 228	536.2522	7.3418	21.8	0	0.0003	1045- BIT 150-1045 -Control
Unknown 229	319.2189	5.6144	11.6	0.0007	0.0023	1045- BIT 150-1045 -Control
Unknown 23	580.338	7.1149	22.5	0	0.0003	1045- BIT 150-1045 -Control
Unknown 231	584.2853	6.2933	22.4	0	0.0003	1045- BIT 150-1045 -Control
Unknown 232	279.1821	5.6434	5.85	0.010609	0.0178	1045- MIT 25-1045- BIT 150
Unknown 233	269.212	5.7302	20.5	0.0001	0.0004	1045- BIT 150-1045 -Control
Unknown 234	431.2388	7.0358	10.8	0.001	0.0028	1045- BIT 150-1045 -Control
Unknown 235	423.1416	0.81	22.6	0	0.0003	1045- BIT 150-1045 -Control
Unknown 237	370.2959	7.5193	7.18	0.0051085	0.0098	1045- BIT 150-1045 -Control
Unknown 238	693.4294	6.7082	23.2	0	0.0002	1045- BIT 150-1045 -Control
Unknown 241	785.4942	6.7073	31.1	0	0.0001	1045- BIT 150-1045 -Control
Unknown 243	215.165	3.8734	24.4	0	0.0002	1045- BIT 150-1045 -Control
Unknown 245	211.0838	3.5591	17.5	0.0001	0.0006	1045- BIT 150-1045 -Control
Unknown 246	606.0754	0.6431	85.7	0	0	1045- BIT 150-1045 -Control
Unknown 247	557.4538	7.0338	30	0	0.0001	1045- BIT 150-1045 -Control
Unknown 248	297.2419	5.6455	12.8	0.0005	0.0017	1045- BIT 150-1045 -Control
Unknown 249	313.2261	5.0277	6.04	0.0095018	0.0162	1045- CIT 4-1045- BIT 150
Unknown 25	299.2585	6.8986	5.18	0.015848	0.0249	
Unknown 250	332.1639	5.1331	7.67	0.0039899	0.0084	1045- CIT 4-1045- BIT 150
Unknown 251	550.2635	2.6907	9.06	0.0021	0.005	1045- CIT 4-1045 -Control
Unknown 252	551.315	0.9023	6.19	0.0087495	0.0152	1045- CIT 4-1045 -Control
Unknown 255	714.4932	8.8577	58.9	0	0	1045- BIT 150-1045 -Control
Unknown 257	379.2637	6.3686	18	0.0001	0.0005	1045- MIT 25-1045 -Control
Unknown 259	605.4099	6.6391	33.7	0	0.0001	1045- BIT 150-1045 -Control
Unknown 260	300.192	1.1856	8.08	0.0032733	0.0073	1045- CIT 4-1045 -Control
Unknown 262	543.2522	5.6313	7.9	0.003572	0.0077	1045- BIT 150-1045 -Control
Unknown 263	223.1701	5.5585	11.7	0.0007	0.0022	1045- BIT 150-1045 -Control
Unknown 264	507.4408	6.6374	34.7	0	0.0001	1045- BIT 150-1045 -Control
Unknown 266	403.2122	6.7381	6.08	0.0093109	0.0159	1045- CIT 4-1045- BIT 150
Unknown 267	335.2202	6.6508	25.8	0	0.0002	1045- BIT 150-1045 -Control
Unknown 268	213.1494	4.0152	10.1	0.0013	0.0036	1045- BIT 150-1045 -Control
Unknown 27	646.1877	6.0799	6.65	0.0067628	0.0123	1045- BIT 150-1045 -Control
Unknown 270	136.0497	0.975	67.9	0	0	1045- BIT 150-1045 -Control
Unknown 271	393.2413	5.5984	4.5	0.024625	0.0361	
Unknown 272	468.0108	5.9611	6.98	0.005689	0.0107	1045- BIT 150-1045- Control
Unknown 273	499.2281	7.0498	25.1	0	0.0002	1045- BIT 150-1045 -Control
Unknown 275	473.353	5.908	5.61	0.012204	0.0201	1045- MIT 25-1045- BIT 150
Unknown 276	243.1963	4.6443	30.7	0	0.0001	1045- BIT 150-1045 -Control
Unknown 28	249.1319	0.9743	7.65	0.0040269	0.0084	1045- CIT 4-1045- BIT 150

Unknown 280	263.128	1.1323	20.2	0.0001	0.0004	1045- CIT 4-1045 -Control
Unknown 281	351.2136	5.7322	33.6	0	0.0001	1045- BIT 150-1045 -Control
Unknown 284	635.394	7.7405	12.9	0.0005	0.0016	1045- BIT 150-1045 -Control
Unknown 285	605.4103	6.7269	53.8	0	0	1045- BIT 150-1045 -Control
Unknown 288	409.3314	7.3124	4.13	0.031667	0.0452	
Unknown 289	330.1239	0.6706	3.97	0.035353	0.0499	
Unknown 29	363.2519	7.5621	6.74	0.0064567	0.0119	1045- MIT 25-1045- BIT 150
Unknown 290	533.3833	8.048	6.86	0.0060554	0.0113	1045- CIT 4-1045- BIT 150
Unknown 291	229.0871	2.5228	11.9	0.0006	0.0021	1045- BIT 150-1045 -Control
Unknown 292	549.3128	7.4908	16	0.0002	0.0008	1045- BIT 150-1045 -Control
Unknown 293	551.319	1.2472	7.63	0.0040799	0.0085	1045- CIT 4-1045 -Control
Unknown 295	263.1631	4.9644	4.86	0.019458	0.0296	
Unknown 296	658.4468	8.6581	112	0	0	1045- BIT 150-1045 -Control
Unknown 297	698.569	7.2046	4.04	0.033623	0.0477	
Unknown 298	395.2335	6.659	17.9	0.0001	0.0005	1045- BIT 150-1045 -Control
Unknown 299	593.4651	7.131	12.7	0.0005	0.0017	1045- MIT 25-1045 -Control
Unknown 3	239.1018	4.4135	15.8	0.0002	0.0008	1045- BIT 150-1045 -Control
Unknown 30	371.1422	4.0174	5.25	0.015205	0.0241	
Unknown 300	733.463	6.4449	11.7	0.0007	0.0022	1045- MIT 25-1045 -Control
Unknown 303	321.24	7.0252	13.5	0.0004	0.0014	1045- BIT 150-1045 -Control
Unknown 304	277.1086	1.0654	50	0	0	1045- BIT 150-1045 -Control
Unknown 305	597.4114	6.7162	27.1	0	0.0001	1045- BIT 150-1045 -Control
Unknown 307	663.2897	5.8769	21.1	0	0.0003	1045- BIT 150-1045 -Control
Unknown 308	403.2105	6.6555	5.25	0.015167	0.0241	
Unknown 309	206.0032	0.7684	8.08	0.003269	0.0073	1045- CIT 4-1045 -Control
Unknown 31	377.1535	4.0179	4.54	0.023973	0.0355	
Unknown 310	522.4269	7.5124	15.2	0.0002	0.001	1045- BIT 150-1045 -Control
Unknown 311	301.1674	6.4947	7.08	0.005395	0.0103	1045- BIT 150-1045 -Control
Unknown 313	585.4862	7.2527	9.72	0.0016	0.0041	1045- BIT 150-1045 -Control
Unknown 314	591.2905	6.5969	17.3	0.0001	0.0006	1045- BIT 150-1045 -Control
Unknown 315	394.3279	7.1666	8.67	0.0024788	0.0058	1045- BIT 150-1045 -Control
Unknown 316	227.165	4.4826	7.29	0.0048394	0.0095	1045- CIT 4-1045- BIT 150
Unknown 318	281.3012	7.2435	20.2	0.0001	0.0004	1045- BIT 150-1045 -Control
Unknown 319	439.2562	5.8629	14.1	0.0003	0.0012	1045- BIT 150-1045 -Control
Unknown 32	183.1389	4.455	16.4	0.0002	0.0007	1045- BIT 150-1045 -Control
Unknown 321	312.1925	1.2564	8.4	0.0028113	0.0064	1045- CIT 4-1045 -Control
Unknown 322	451.3055	6.8403	6.6	0.006949	0.0126	1045- BIT 150-1045 -Control
Unknown 323	610.3014	6.4414	23	0	0.0003	1045- BIT 150-1045 -Control
Unknown 325	653.4703	7.2619	11.7	0.0007	0.0022	1045- BIT 150-1045 -Control
Unknown 326	467.1937	6.4489	14.9	0.0002	0.001	1045- BIT 150-1045 -Control
Unknown 327	450.265	6.7239	20.7	0	0.0004	1045- BIT 150-1045 -Control
Unknown 328	563.5034	7.2649	19.5	0.0001	0.0004	1045- BIT 150-1045 -Control
Unknown 33	662.5205	7.274	7.57	0.0042089	0.0086	1045- CIT 4-1045- BIT 150
Unknown 330	213.1522	7.8944	14.1	0.0003	0.0012	1045- BIT 150-1045 -Control
Unknown 331	545.3951	6.6344	14.7	0.0003	0.0011	1045- BIT 150-1045 -Control
Unknown 332	427.1257	3.2371	8.14	0.0031729	0.0071	1045- CIT 4-1045- BIT 150
Unknown 335	153.1284	4.0074	4.6	0.022996	0.0342	
Unknown 336	629.3119	1.0729	8.37	0.0028515	0.0065	1045- CIT 4-1045 -Control

Unknown 339	295.264	7.567	6.13	0.0090351	0.0156	1045- MIT 25-1045- BIT 150
Unknown 34	141.1283	3.5857	4.52	0.024284	0.0357	
Unknown 340	349.2359	7.0403	13.7	0.0004	0.0013	1045- BIT 150-1045 -Control
Unknown 341	459.2696	7.5759	4.4	0.026287	0.0383	
Unknown 345	448.3023	6.6399	4.1	0.032297	0.0459	
Unknown 347	295.2276	5.5138	15.3	0.0002	0.0009	1045- BIT 150-1045 -Control
Unknown 348	297.243	5.9344	6.2	0.008683	0.0151	1045- CIT 4-1045- BIT 150
Unknown 349	260.0823	0.6589	6.24	0.0085138	0.0149	1045- CIT 4-1045 -Control
Unknown 35	323.1839	4.9526	7.15	0.0052039	0.01	1045- CIT 4-1045- BIT 150
Unknown 350	323.1811	4.1863	15.9	0.0002	0.0008	1045- BIT 150-1045 -Control
Unknown 351	425.2269	7.0228	12.6	0.0005	0.0018	1045- BIT 150-1045 -Control
Unknown 352	477.2467	5.835	5.55	0.012694	0.0208	1045- CIT 4-1045- BIT 150
Unknown 353	471.26	2.6996	5.01	0.017701	0.0273	
Unknown 355	319.225	5.8515	15.9	0.0002	0.0008	1045- BIT 150-1045 -Control
Unknown 356	321.2055	6.67	10.5	0.0011	0.0031	1045- BIT 150-1045 -Control
Unknown 357	297.2419	6.4335	33.8	0	0.0001	1045- BIT 150-1045 -Control
Unknown 358	692.5228	7.0386	10	0.0014	0.0036	1045- BIT 150-1045 -Control
Unknown 359	685.4629	5.6313	7.6	0.0041461	0.0086	1045- BIT 150-1045 -Control
Unknown 36	239.2013	6.3349	25.1	0	0.0002	1045- BIT 150-1045 -Control
Unknown 360	435.5701	0.9788	15.6	0.0002	0.0009	1045- BIT 150-1045 -Control
Unknown 362	613.5183	7.5816	7.38	0.0046321	0.0092	1045- MIT 25-1045- BIT 150
Unknown 363	618.0854	0.6438	23.7	0	0.0002	1045- BIT 150-1045 -Control
Unknown 364	271.2277	6.2661	14	0.0003	0.0013	1045- MIT 25-1045 -Control
Unknown 365	552.3306	6.6786	33.9	0	0.0001	1045- BIT 150-1045 -Control
Unknown 366	610.054	0.6662	9.29	0.0019	0.0047	1045- CIT 4-1045 -Control
Unknown 367	653.2614	2.7512	13.5	0.0004	0.0014	1045- BIT 150-1045 -Control
Unknown 368	367.2472	5.993	5.05	0.017257	0.0268	
Unknown 369	629.3435	7.8543	12.3	0.0006	0.0019	1045- BIT 150-1045 -Control
Unknown 37	164.0806	0.8833	7.21	0.0050458	0.0098	1045- CIT 4-1045 -Control
Unknown 372	653.4688	7.2551	7.33	0.0047284	0.0093	1045- BIT 150-1045 -Control
Unknown 373	241.1088	0.8673	10.2	0.0013	0.0034	1045- CIT 4-1045 -Control
Unknown 374	167.0635	3.46	36.9	0	0.0001	1045- BIT 150-1045 -Control
Unknown 375	632.3637	3.1126	9.44	0.0018	0.0045	1045- CIT 4-1045 -Control
Unknown 376	670.3562	7.0218	28.6	0	0.0001	1045- BIT 150-1045 -Control
Unknown 377	543.2561	5.8623	7.45	0.0044624	0.009	1045- BIT 150-1045 -Control
Unknown 378	452.2791	7.231	16	0.0002	0.0008	1045- BIT 150-1045 -Control
Unknown 38	492.2763	5.6852	11.3	0.0008	0.0025	1045- CIT 4-1045- BIT 150
Unknown 381	304.096	0.6704	6.83	0.0061366	0.0114	1045- CIT 4-1045 -Control
Unknown 382	733.4649	7.9738	20.7	0	0.0004	1045- BIT 150-1045 -Control
Unknown 383	525.195	0.8382	5.75	0.011251	0.0188	1045- BIT 150-1045 -Control
Unknown 384	241.0825	2.5873	37.3	0	0.0001	1045- BIT 150-1045 -Control
Unknown 385	476.3201	7.1546	11.4	0.0008	0.0024	1045- BIT 150-1045 -Control
Unknown 386	577.2779	6.6839	19.6	0.0001	0.0004	1045- BIT 150-1045 -Control
Unknown 388	291.08	3.9927	5.9	0.010337	0.0174	1045- CIT 4-1045- BIT 150
Unknown 389	644.3403	6.7802	23.7	0	0.0002	1045- BIT 150-1045 -Control
Unknown 39	365.2277	5.6518	15.1	0.0002	0.001	1045- BIT 150-1045 -Control
Unknown 390	661.4129	7.9033	4.98	0.01802	0.0277	
Unknown 392	559.4239	6.4501	21.8	0	0.0003	1045- BIT 150-1045 -Control

Unknown 394	447.2366	5.6187	9.38	0.0018	0.0045	1045- BIT 150-1045 -Control
Unknown 395	726.469	6.6264	20.8	0	0.0003	1045- BIT 150-1045 -Control
Unknown 396	485.1627	0.7943	6.64	0.0067998	0.0123	1045- CIT 4-1045 -Control
Unknown 397	300.2509	5.6552	28.3	0	0.0001	1045- BIT 150-1045 -Control
Unknown 398	385.1912	6.4503	15.8	0.0002	0.0008	1045- BIT 150-1045 -Control
Unknown 399	269.212	4.9486	5.91	0.010268	0.0173	1045- CIT 4-1045- BIT 150
Unknown 4	565.276	6.4481	44.9	0	0	1045- BIT 150-1045 -Control
Unknown 40	249.1288	0.8888	14.1	0.0003	0.0012	1045- BIT 150-1045 -Control
Unknown 400	735.4763	7.272	5.46	0.013333	0.0216	
Unknown 402	333.2099	6.2241	16.5	0.0001	0.0007	1045- BIT 150-1045 -Control
Unknown 403	607.2587	4.711	17.2	0.0001	0.0006	1045- BIT 150-1045 -Control
Unknown 404	351.1402	3.2421	16.1	0.0002	0.0008	1045- BIT 150-1045 -Control
Unknown 405	375.1925	4.0744	21.4	0	0.0003	1045- BIT 150-1045 -Control
Unknown 406	251.2013	6.2202	29.6	0	0.0001	1045- BIT 150-1045 -Control
Unknown 410	434.181	0.9495	13.4	0.0004	0.0014	1045- CIT 4-1045 -Control
Unknown 415	799.6128	6.6345	9.15	0.002	0.0049	1045- CIT 4-1045- BIT 150
Unknown 416	313.0985	1.0775	51.3	0	0	1045- BIT 150-1045 -Control
Unknown 42	224.0344	0.857	8.82	0.0023125	0.0055	1045- CIT 4-1045- BIT 150
Unknown 420	201.1495	3.9101	4.93	0.018552	0.0283	
Unknown 421	481.2603	6.0611	32.7	0	0.0001	1045- BIT 150-1045 -Control
Unknown 422	371.2359	5.9303	12	0.0006	0.0021	1045- BIT 150-1045 -Control
Unknown 423	345.1201	3.2461	12.6	0.0005	0.0018	1045- BIT 150-1045 -Control
Unknown 426	763.4695	2.9375	6.34	0.0080199	0.0142	1045- CIT 4-1045 -Control
Unknown 427	263.1031	0.8717	6.46	0.0075167	0.0135	1045- CIT 4-1045 -Control
Unknown 428	576.0363	0.6951	5.21	0.015542	0.0246	
Unknown 429	458.2922	6.5012	10.5	0.0011	0.0032	1045- BIT 150-1045 -Control
Unknown 431	377.2405	7.2608	5.87	0.010484	0.0177	1045- MIT 25-1045- BIT 150
Unknown 432	325.1974	4.6641	9.93	0.0014	0.0038	1045- CIT 4-1045- BIT 150
Unknown 434	365.1549	0.8245	9.28	0.0019	0.0047	1045- CIT 4-1045 -Control
Unknown 435	633.4467	5.6144	4.49	0.024686	0.0361	
Unknown 437	519.2252	6.0398	8.02	0.0033724	0.0074	1045- BIT 150-1045 -Control
Unknown 438	391.2065	1.4375	9.98	0.0014	0.0037	1045- CIT 4-1045- BIT 150
Unknown 439	240.2023	6.333	37.6	0	0.0001	1045- BIT 150-1045 -Control
Unknown 44	730.4991	8.489	9.1	0.002	0.005	1045- MIT 25-1045 -Control
Unknown 440	351.2149	4.9482	7.4	0.0045801	0.0091	1045- CIT 4-1045- BIT 150
Unknown 441	713.5268	5.5184	11.4	0.0008	0.0024	1045- BIT 150-1045 -Control
Unknown 443	307.224	6.6382	10	0.0014	0.0037	1045- BIT 150-1045 -Control
Unknown 444	326.1281	2.7581	10.2	0.0013	0.0034	1045- BIT 150-1045 -Control
Unknown 445	169.1597	5.1242	12.1	0.0006	0.002	1045- BIT 150-1045 -Control
Unknown 446	141.1283	3.2466	7.44	0.0044881	0.009	1045- CIT 4-1045- BIT 150
Unknown 447	324.1894	0.9653	11.7	0.0007	0.0022	1045- BIT 150-1045 -Control
Unknown 448	284.2604	7.2611	12	0.0006	0.0021	1045- BIT 150-1045 -Control
Unknown 449	632.2675	2.6944	5.67	0.011807	0.0195	1045- CIT 4-1045- BIT 150
Unknown 45	251.2012	6.3499	22.7	0	0.0003	1045- BIT 150-1045 -Control
Unknown 450	279.1569	5.0481	22.8	0	0.0003	1045- BIT 150-1045 -Control
Unknown 453	365.2315	6.4199	55.6	0	0	1045- BIT 150-1045 -Control
Unknown 454	337.2	5.7605	26.6	0	0.0002	1045- BIT 150-1045 -Control
Unknown 458	259.0931	0.9872	8.21	0.0030775	0.0069	1045- CIT 4-1045 -Control

Unknown 459	573.2444	0.8806	5.4	0.013887	0.0224	
Unknown 46	171.1024	1.3689	18.6	0.0001	0.0005	1045- BIT 150-1045 -Control
Unknown 460	460.0302	0.7684	4.2	0.03003	0.0431	
Unknown 462	197.1545	5.0423	40.9	0	0	1045- BIT 150-1045 -Control
Unknown 463	241.1806	4.9629	15.5	0.0002	0.0009	1045- BIT 150-1045 -Control
Unknown 465	781.4812	5.632	4.53	0.024098	0.0355	
Unknown 466	788.3985	2.6827	10.4	0.0012	0.0033	1045- CIT 4-1045 -Control
Unknown 467	687.4139	6.7169	68.1	0	0	1045- BIT 150-1045 -Control
Unknown 468	594.2698	0.8015	4.52	0.024269	0.0357	
Unknown 47	507.4406	6.7216	17.7	0.0001	0.0006	1045- BIT 150-1045 -Control
Unknown 470	513.3305	7.5065	29.6	0	0.0001	1045- BIT 150-1045 -Control
Unknown 471	500.2756	7.3473	6.73	0.0064919	0.0119	1045- BIT 150-1045 -Control
Unknown 472	311.1718	0.8128	11.8	0.0007	0.0022	1045- BIT 150-1045 -Control
Unknown 473	246.0724	0.879	25.1	0	0.0002	1045- BIT 150-1045 -Control
Unknown 475	648.4274	8.1787	16.3	0.0002	0.0008	1045- MIT 25-1045 -Control
Unknown 476	689.4413	8.1207	38.7	0	0	1045- BIT 150-1045 -Control
Unknown 477	267.1235	5.0195	21.5	0	0.0003	1045- BIT 150-1045 -Control
Unknown 478	339.2076	7.229	11.5	0.0008	0.0024	1045- MIT 25-1045 -Control
Unknown 48	372.2129	1.0292	10	0.0014	0.0036	1045- CIT 4-1045 -Control
Unknown 480	507.4872	8.4261	13.4	0.0004	0.0014	1045- BIT 150-1045 -Control
Unknown 482	297.2436	5.7122	17.6	0.0001	0.0006	1045- BIT 150-1045 -Control
Unknown 483	489.2346	2.2137	7.58	0.0041867	0.0086	1045- CIT 4-1045 -Control
Unknown 484	503.2869	2.2819	22.5	0	0.0003	1045- CIT 4-1045 -Control
Unknown 485	388.154	5.8242	6.46	0.0075258	0.0135	1045- BIT 150-1045 -Control
Unknown 486	445.2558	7.5821	6.33	0.0080602	0.0142	1045- MIT 25-1045- BIT 150
Unknown 487	584.4889	5.8508	18.6	0.0001	0.0005	1045- BIT 150-1045 -Control
Unknown 488	213.1371	3.3851	15	0.0002	0.001	1045- BIT 150-1045 -Control
Unknown 489	481.3179	7.73	13.4	0.0004	0.0014	1045- BIT 150-1045 -Control
Unknown 49	509.2884	6.6752	20.2	0.0001	0.0004	1045- BIT 150-1045 -Control
Unknown 490	296.2674	7.5554	6.05	0.0094703	0.0161	1045- MIT 25-1045- BIT 150
Unknown 491	227.1644	4.5199	11.9	0.0007	0.0021	1045- BIT 150-1045 -Control
Unknown 492	187.0654	0.8873	5.01	0.017664	0.0273	
Unknown 493	476.2897	7.0806	4.08	0.032752	0.0465	
Unknown 496	675.2429	2.7503	10.5	0.0011	0.0032	1045- BIT 150-1045 -Control
Unknown 498	510.3192	7.1398	4.31	0.027985	0.0406	
Unknown 499	395.3279	7.2667	8.93	0.0022	0.0053	1045- BIT 150-1045 -Control
Unknown 5	222.0321	0.8509	9.36	0.0018	0.0046	1045- BIT 150-1045 -Control
Unknown 50	138.9371	0.674	10.1	0.0013	0.0036	1045- BIT 150-1045 -Control
Unknown 500	549.3278	6.923	16.3	0.0002	0.0008	1045- BIT 150-1045 -Control
Unknown 501	756.3948	2.2085	13.6	0.0004	0.0013	1045- CIT 4-1045 -Control
Unknown 502	702.4728	8.467	66.2	0	0	1045- BIT 150-1045 -Control
Unknown 503	513.301	7.5342	5.52	0.012871	0.021	
Unknown 504	545.3956	6.7046	14.5	0.0003	0.0011	1045- BIT 150-1045 -Control
Unknown 505	489.2649	2.2497	5.36	0.014237	0.0228	
Unknown 506	639.4615	5.6108	16.8	0.0001	0.0007	1045- BIT 150-1045 -Control
Unknown 507	187.1337	3.2365	10.9	0.001	0.0028	1045- BIT 150-1045 -Control
Unknown 508	259.098	0.885	7.1	0.0053359	0.0102	1045- CIT 4-1045 -Control
Unknown 509	667.4894	7.2618	10.2	0.0013	0.0035	1045- BIT 150-1045 -Control

Unknown 51	307.2251	6.7165	5.45	0.013465	0.0217	
Unknown 510	432.2429	7.0134	5.12	0.01649	0.0258	
Unknown 514	492.3199	7.5757	4.54	0.024005	0.0355	
Unknown 515	495.2727	6.351	43.1	0	0	1045- BIT 150-1045 -Control
Unknown 516	569.3954	8.2611	21.4	0	0.0003	1045- BIT 150-1045 -Control
Unknown 517	672.3271	0.964	7.71	0.0039144	0.0083	1045- CIT 4-1045 -Control
Unknown 518	684.3854	2.9293	11.3	0.0008	0.0025	1045- BIT 150-1045 -Control
Unknown 519	349.2049	6.6392	21.3	0	0.0003	1045- BIT 150-1045 -Control
Unknown 52	424.2482	6.4562	26.4	0	0.0002	1045- BIT 150-1045 -Control
Unknown 520	389.1952	5.9791	22.1	0	0.0003	1045- BIT 150-1045 -Control
Unknown 521	551.3303	7.8581	31.5	0	0.0001	1045- BIT 150-1045 -Control
Unknown 522	628.2852	7.3485	9.59	0.0016	0.0042	1045- BIT 150-1045 -Control
Unknown 523	557.4526	7.0102	6.07	0.009332	0.0159	1045- MIT 25-1045- BIT 150
Unknown 525	545.2812	1.2402	5.72	0.011471	0.0191	1045- CIT 4-1045 -Control
Unknown 526	587.3814	7.8853	19	0.0001	0.0005	1045- BIT 150-1045 -Control
Unknown 527	732.3867	2.22	6.14	0.0089944	0.0155	1045- CIT 4-1045- BIT 150
Unknown 528	527.3503	7.7672	11.8	0.0007	0.0022	1045- BIT 150-1045 -Control
Unknown 529	549.4287	7.1201	7.5	0.0043497	0.0088	1045- CIT 4-1045- BIT 150
Unknown 530	357.1856	5.6128	51.8	0	0	1045- BIT 150-1045 -Control
Unknown 531	181.0736	3.603	13.3	0.0004	0.0015	1045- BIT 150-1045 -Control
Unknown 532	673.2936	6.6851	17.5	0.0001	0.0006	1045- BIT 150-1045 -Control
Unknown 533	642.2997	7.3434	8.45	0.0027477	0.0063	1045- BIT 150-1045 -Control
Unknown 534	450.2657	6.8136	15.9	0.0002	0.0008	1045- BIT 150-1045 -Control
Unknown 535	585.3683	7.7909	22.1	0	0.0003	1045- CIT 4-1045 -Control
Unknown 537	749.492	7.2676	11.2	0.0009	0.0026	1045- BIT 150-1045 -Control
Unknown 538	450.3123	7.0713	17.4	0.0001	0.0006	1045- BIT 150-1045 -Control
Unknown 539	630.4129	8.4177	20.8	0	0.0003	1045- BIT 150-1045 -Control
Unknown 54	354.1957	1.4558	29.6	0	0.0001	1045- BIT 150-1045 -Control
Unknown 540	279.1572	5.1404	23.3	0	0.0002	1045- BIT 150-1045 -Control
Unknown 541	269.2088	5.1759	7.01	0.0056	0.0106	1045- CIT 4-1045- BIT 150
Unknown 542	269.1371	3.2301	10.6	0.0011	0.0031	1045- BIT 150-1045 -Control
Unknown 543	728.3519	2.2736	4.82	0.019965	0.0303	
Unknown 544	573.3582	7.4955	15.1	0.0002	0.001	1045- BIT 150-1045 -Control
Unknown 546	363.2256	7.0631	14.5	0.0003	0.0011	1045- BIT 150-1045 -Control
Unknown 547	441.1491	2.2774	55.6	0	0	1045- BIT 150-1045 -Control
Unknown 548	473.2108	6.4503	7.93	0.0035137	0.0076	1045- CIT 4-1045- BIT 150
Unknown 551	442.3266	7.4247	6.2	0.0087072	0.0152	1045- BIT 150-1045 -Control
Unknown 552	788.5422	7.1391	6.71	0.0065662	0.012	1045- BIT 150-1045 -Control
Unknown 554	399.147	0.8299	9.56	0.0017	0.0043	1045- CIT 4-1045 -Control
Unknown 555	560.8065	2.8508	28.3	0	0.0001	1045- BIT 150-1045 -Control
Unknown 556	560.0336	0.7844	12.6	0.0005	0.0018	1045- BIT 150-1045 -Control
Unknown 557	311.2184	5.0577	4.8	0.02015	0.0305	
Unknown 558	836.4304	2.4836	5.74	0.011348	0.0189	1045- CIT 4-1045 -Control
Unknown 56	529.4204	6.7146	17.7	0.0001	0.0006	1045- BIT 150-1045 -Control
Unknown 560	186.1102	1.0985	8.51	0.0026684	0.0061	1045- CIT 4-1045 -Control
Unknown 562	246.0689	0.8607	24.3	0	0.0002	1045- BIT 150-1045 -Control
Unknown 565	523.3053	7.0207	15.4	0.0002	0.0009	1045- BIT 150-1045 -Control
Unknown 566	629.4192	8.2643	6.37	0.0078894	0.014	1045- CIT 4-1045- BIT 150

Unknown 567	538.3232	2.248	11.7	0.0007	0.0022	1045- CIT 4-1045 -Control
Unknown 568	485.2722	6.651	5.28	0.014898	0.0238	
Unknown 570	356.2788	6.9826	13.8	0.0003	0.0013	1045- BIT 150-1045 -Control
Unknown 571	198.074	0.7848	6.13	0.0090363	0.0156	1045- CIT 4-1045- BIT 150
Unknown 572	399.3163	6.3839	31.5	0	0.0001	1045- CIT 4-1045 -Control
Unknown 574	642.3264	6.3093	46.2	0	0	1045- BIT 150-1045 -Control
Unknown 575	805.6534	7.0927	16.8	0.0001	0.0007	1045- BIT 150-1045 -Control
Unknown 576	611.8564	3.4083	17.4	0.0001	0.0006	1045- CIT 4-1045 -Control
Unknown 577	645.2632	5.974	31.6	0	0.0001	1045- BIT 150-1045 -Control
Unknown 578	494.2883	6.7944	21.7	0	0.0003	1045- BIT 150-1045 -Control
Unknown 58	842.5731	8.7185	7.13	0.0052662	0.0101	1045- BIT 150-1045 -Control
Unknown 580	676.4537	8.45	37.2	0	0.0001	1045- BIT 150-1045 -Control
Unknown 581	456.2002	3.8922	11.5	0.0008	0.0023	1045- BIT 150-1045 -Control
Unknown 582	411.2073	6.7168	9.21	0.0019	0.0048	1045- CIT 4-1045- BIT 150
Unknown 585	464.1156	2.1861	18.4	0.0001	0.0005	1045- BIT 150-1045 -Control
Unknown 586	325.2535	7.0149	7.45	0.0044487	0.0089	1045- CIT 4-1045- BIT 150
Unknown 587	363.2515	7.2648	4.76	0.020726	0.0312	
Unknown 588	323.1028	0.9136	9.04	0.0021	0.0051	1045- CIT 4-1045 -Control
Unknown 589	436.3025	6.9744	6.44	0.007605	0.0136	1045- BIT 150-1045 -Control
Unknown 591	477.3916	6.4455	15.9	0.0002	0.0008	1045- BIT 150-1045 -Control
Unknown 593	349.2061	6.7258	10.4	0.0012	0.0032	1045- CIT 4-1045- BIT 150
Unknown 595	271.2268	5.3281	7.64	0.0040597	0.0085	1045- CIT 4-1045- BIT 150
Unknown 596	384.3116	7.4966	9.51	0.0017	0.0044	1045- BIT 150-1045 -Control
Unknown 599	277.1939	0.7885	16.6	0.0001	0.0007	1045- BIT 150-1045 -Control
Unknown 60	611.4334	6.7395	16.1	0.0002	0.0008	1045- BIT 150-1045 -Control
Unknown 600	583.2846	0.915	16.1	0.0002	0.0008	1045- CIT 4-1045 -Control
Unknown 601	507.225	7.0356	15.2	0.0002	0.001	1045- BIT 150-1045 -Control
Unknown 602	666.3823	2.2502	9.49	0.0017	0.0044	1045- CIT 4-1045 -Control
Unknown 603	749.4921	7.2669	6.68	0.0066551	0.0122	1045- BIT 150-1045 -Control
Unknown 604	409.1739	4.1502	22.8	0	0.0003	1045- BIT 150-1045 -Control
Unknown 608	495.273	6.4455	7.63	0.0040704	0.0085	1045- BIT 150-1045 -Control
Unknown 609	546.2809	7.3479	10.6	0.0011	0.0031	1045- BIT 150-1045 -Control
Unknown 61	627.4176	6.7158	41.4	0	0	1045- BIT 150-1045 -Control
Unknown 610	673.3308	7.8777	10.8	0.001	0.0029	1045- CIT 4-1045- BIT 150
Unknown 612	557.0392	3.3676	9.55	0.0017	0.0043	1045- BIT 150-1045 -Control
Unknown 613	546.281	7.0052	18	0.0001	0.0005	1045- BIT 150-1045 -Control
Unknown 614	399.1102	0.8432	4.79	0.020255	0.0306	
Unknown 615	776.5807	7.2366	5.09	0.01681	0.0262	
Unknown 616	492.3207	7.687	4.16	0.031001	0.0443	
Unknown 618	451.3066	4.542	5.47	0.013323	0.0216	
Unknown 62	263.0804	4.0698	6	0.0097266	0.0166	1045- BIT 150-1045 -Control
Unknown 620	552.2226	0.8378	5.91	0.010257	0.0173	1045- CIT 4-1045- BIT 150
Unknown 621	377.0857	0.6806	14.2	0.0003	0.0012	1045- CIT 4-1045 -Control
Unknown 622	563.1138	0.8098	4.36	0.026931	0.0392	
Unknown 623	405.188	4.9678	11.2	0.0008	0.0025	1045- MIT 25-1045 -Control
Unknown 624	316.2573	5.094	10.8	0.001	0.0029	1045- CIT 4-1045- BIT 150
Unknown 626	674.4433	8.2729	67.8	0	0	1045- BIT 150-1045 -Control
Unknown 627	397.1583	3.3409	14.9	0.0002	0.001	1045- BIT 150-1045 -Control

Unknown 629	305.2149	6.5291	5.55	0.012648	0.0207	1045- BIT 150-1045 -Control
Unknown 63	365.2177	5.8548	6.14	0.0089613	0.0155	1045- CIT 4-1045- BIT 150
Unknown 630	406.3028	7.1081	7.89	0.0035763	0.0077	1045- BIT 150-1045 -Control
Unknown 631	361.1617	5.0403	10.1	0.0014	0.0036	1045- BIT 150-1045 -Control
Unknown 632	642.3263	6.3604	18.9	0.0001	0.0005	1045- BIT 150-1045 -Control
Unknown 633	338.0451	0.8001	16.8	0.0001	0.0007	1045- BIT 150-1045 -Control
Unknown 635	295.2263	5.7641	5.35	0.014308	0.0229	
Unknown 636	679.581	6.0231	23	0	0.0003	1045- BIT 150-1045 -Control
Unknown 638	314.2684	6.2739	18.9	0.0001	0.0005	1045- BIT 150-1045 -Control
Unknown 639	596.2705	2.6976	8.11	0.0032274	0.0072	1045- CIT 4-1045 -Control
Unknown 64	317.1526	0.8165	5.97	0.0098798	0.0168	1045- CIT 4-1045 -Control
Unknown 640	597.4104	6.6389	27.5	0	0.0001	1045- BIT 150-1045 -Control
Unknown 641	761.4974	8.1855	75.6	0	0	1045- BIT 150-1045 -Control
Unknown 642	703.4649	8.2555	45.8	0	0	1045- BIT 150-1045 -Control
Unknown 644	678.098	0.6512	8.64	0.0025179	0.0059	1045- BIT 150-1045 -Control
Unknown 646	607.3153	6.9659	4.5	0.024521	0.036	
Unknown 647	369.2172	6.4453	8.57	0.0025951	0.006	1045- CIT 4-1045- BIT 150
Unknown 649	636.2852	7.3364	8.14	0.0031744	0.0071	1045- BIT 150-1045 -Control
Unknown 65	366.3011	6.6708	11.9	0.0007	0.0021	1045- BIT 150-1045 -Control
Unknown 650	550.3577	3.1316	23.9	0	0.0002	1045- CIT 4-1045 -Control
Unknown 652	699.4787	5.8534	4.69	0.021639	0.0324	
Unknown 653	424.5792	0.9885	16.2	0.0002	0.0008	1045- BIT 150-1045 -Control
Pantothenate	218.1045	2.8907	22.2	0	0.0003	1045- BIT 150-1045 -Control
Unknown 655	635.2606	5.1245	11.6	0.0007	0.0023	1045- CIT 4-1045- BIT 150
Unknown 656	559.3427	7.5018	15.3	0.0002	0.0009	1045- BIT 150-1045 -Control
Unknown 657	634.3243	2.8116	19.2	0.0001	0.0004	1045- CIT 4-1045 -Control
Unknown 658	427.2085	4.9617	6.71	0.0065548	0.012	1045- CIT 4-1045- BIT 150
Unknown 659	316.1633	1.1513	5.95	0.010011	0.017	1045- CIT 4-1045 -Control
Unknown 660	172.0912	0.9069	8.06	0.0033	0.0073	1045- CIT 4-1045 -Control
Unknown 661	708.5774	7.305	5.6	0.01228	0.0202	1045- BIT 150-1045 -Control
Unknown 662	528.2816	2.6918	9.61	0.0016	0.0042	1045- CIT 4-1045 -Control
Unknown 663	628.0599	0.6145	4.21	0.029841	0.0429	
Unknown 664	600.265	2.1902	6.09	0.0092619	0.0159	1045- CIT 4-1045- BIT 150
Unknown 665	319.225	6.4247	17.9	0.0001	0.0005	1045- BIT 150-1045 -Control
Unknown 666	580.3618	7.3392	15.1	0.0002	0.001	1045- BIT 150-1045 -Control
Unknown 667	323.0417	3.2432	7.07	0.0054201	0.0104	1045- BIT 150-1045 -Control
Unknown 668	277.1225	7.4917	16.6	0.0001	0.0007	1045- BIT 150-1045 -Control
Unknown 67	424.2483	6.5768	18.8	0.0001	0.0005	1045- BIT 150-1045 -Control
Unknown 670	173.1168	2.5195	5.75	0.011228	0.0188	1045- MIT 25-1045- BIT 150
Unknown 671	650.3822	2.223	9.64	0.0016	0.0042	1045- CIT 4-1045 -Control
Unknown 673	481.156	0.8125	16.4	0.0002	0.0008	1045- BIT 150-1045 -Control
Unknown 674	561.3584	7.848	26.8	0	0.0001	1045- BIT 150-1045 -Control
Unknown 675	527.2517	2.5756	7.5	0.0043405	0.0088	1045- CIT 4-1045 -Control
Unknown 676	241.1401	3.2813	16.3	0.0002	0.0008	1045- BIT 150-1045 -Control
Unknown 677	565.045	0.6515	8.9	0.0022	0.0054	1045- BIT 150-1045 -Control
Unknown 678	478.2932	6.5324	8.76	0.0023835	0.0056	1045- CIT 4-1045- BIT 150
Unknown 679	582.2693	5.8976	16.3	0.0002	0.0008	1045- BIT 150-1045 -Control
Unknown 68	395.1716	0.8887	5.22	0.015443	0.0244	

Unknown 680	455.4091	6.4433	16.4	0.0002	0.0007	1045- BIT 150-1045 -Control
Unknown 681	560.2969	7.3446	12.5	0.0005	0.0018	1045- BIT 150-1045 -Control
Unknown 682	707.3384	2.5903	5.71	0.011527	0.0191	1045- CIT 4-1045 -Control
Unknown 684	593.3027	6.642	15.8	0.0002	0.0008	1045- BIT 150-1045 -Control
Unknown 685	568.5306	7.3854	7.93	0.0035114	0.0076	1045- BIT 150-1045 -Control
Unknown 686	468.2721	6.5962	12.7	0.0005	0.0017	1045- BIT 150-1045 -Control
Unknown 687	215.1651	4.5221	6.76	0.0063992	0.0118	1045- CIT 4-1045- BIT 150
Unknown 689	596.259	0.8388	4.94	0.018509	0.0283	
Unknown 69	417.2303	7.041	18.8	0.0001	0.0005	1045- BIT 150-1045 -Control
Unknown 690	290.0915	0.7183	5.64	0.012002	0.0198	1045- CIT 4-1045 -Control
Unknown 694	489.2177	5.539	8.42	0.0027805	0.0063	1045- BIT 150-1045 -Control
Unknown 695	274.1059	0.7795	5.35	0.014283	0.0229	
Unknown 696	585.308	2.6046	11	0.0009	0.0027	1045- CIT 4-1045 -Control
Unknown 697	487.2254	0.9417	4.25	0.029123	0.042	
Unknown 698	699.4786	5.6271	15.1	0.0002	0.001	1045- BIT 150-1045 -Control
Unknown 699	342.1399	0.8108	17	0.0001	0.0007	1045- BIT 150-1045 -Control
Unknown 700	761.4926	7.7642	65.2	0	0	1045- BIT 150-1045 -Control
Unknown 702	634.2352	0.8788	7.5	0.0043495	0.0088	1045- CIT 4-1045 -Control
Unknown 704	685.2727	5.8912	6.98	0.005686	0.0107	1045- CIT 4-1045- BIT 150
Unknown 706	368.1607	0.9037	5.15	0.01613	0.0253	
Unknown 708	812.4401	2.5348	4.62	0.022681	0.0338	
Unknown 709	778.3751	2.2046	9.8	0.0015	0.0039	1045- CIT 4-1045 -Control
Unknown 71	582.2693	5.8302	26	0	0.0002	1045- BIT 150-1045 -Control
Unknown 711	637.4046	7.9579	7.56	0.0042142	0.0086	1045- CIT 4-1045- BIT 150
Unknown 712	109.0179	1.2136	7.07	0.0054318	0.0104	1045- BIT 150-1045 -Control
Unknown 716	326.2082	1.2489	15.6	0.0002	0.0009	1045- CIT 4-1045 -Control
Unknown 717	641.2955	0.9085	5.58	0.012426	0.0204	1045- CIT 4-1045 -Control
Unknown 718	574.3351	2.4239	17	0.0001	0.0007	1045- CIT 4-1045 -Control
Unknown 719	669.2799	7.9167	13.2	0.0004	0.0015	1045- BIT 150-1045 -Control
Unknown 72	351.1264	0.8467	5.16	0.016047	0.0252	
Unknown 720	209.1172	3.2321	6.42	0.0076637	0.0137	1045- CIT 4-1045- BIT 150
Unknown 722	553.2639	2.6913	3.98	0.03505	0.0495	
Unknown 723	335.2159	4.9828	7.72	0.0039021	0.0083	1045- CIT 4-1045- BIT 150
Unknown 724	632.4307	8.5818	14.4	0.0003	0.0011	1045- BIT 150-1045 -Control
Unknown 725	745.3569	2.5635	7.64	0.0040596	0.0085	1045- CIT 4-1045- BIT 150
Unknown 726	272.0065	0.6912	7.37	0.0046547	0.0092	1045- CIT 4-1045- BIT 150
Unknown 727	203.0015	0.4792	7.93	0.0035127	0.0076	1045- CIT 4-1045 -Control
Unknown 728	810.3566	2.2714	8.59	0.0025671	0.006	1045- CIT 4-1045- BIT 150
Unknown 729	467.3423	7.5349	13.5	0.0004	0.0014	1045- MIT 25-1045 -Control
Unknown 73	417.2224	6.7282	6.4	0.007769	0.0138	1045- MIT 25-1045- BIT 150
Unknown 732	241.1805	5.0552	16.1	0.0002	0.0008	1045- BIT 150-1045 -Control
Unknown 733	799.4755	8.3093	107	0	0	1045- BIT 150-1045 -Control
Unknown 735	651.5562	7.5868	6.67	0.0066959	0.0122	1045- MIT 25-1045- BIT 150
Unknown 738	488.3755	7.5142	6.58	0.0070426	0.0127	1045- BIT 150-1045 -Control
Unknown 739	437.3095	7.8513	18.8	0.0001	0.0005	1045- BIT 150-1045 -Control
Unknown 74	494.2564	1.0564	9.2	0.002	0.0048	1045- MIT 25-1045 -Control
Unknown 740	341.1195	2.6234	9.93	0.0014	0.0038	1045- BIT 150-1045 -Control
Unknown 741	387.2743	7.0897	6.12	0.0090639	0.0156	1045- CIT 4-1045- BIT 150

Unknown 742	595.4938	5.6245	24.7	0	0.0002	1045- BIT 150-1045 -Control
Unknown 744	515.3451	7.8561	27	0	0.0001	1045- BIT 150-1045 -Control
Unknown 745	565.2756	6.571	27	0	0.0001	1045- BIT 150-1045 -Control
Unknown 747	225.1044	3.0998	7.43	0.0044939	0.009	1045- BIT 150-1045 -Control
Unknown 748	836.4303	2.7041	7.95	0.0034779	0.0075	1045- CIT 4-1045 -Control
Unknown 749	243.1964	5.4789	6.99	0.0056665	0.0107	1045- CIT 4-1045- BIT 150
Unknown 75	563.2599	6.0325	44.4	0	0	1045- BIT 150-1045 -Control
Unknown 750	617.4758	5.8523	10.1	0.0013	0.0036	1045- BIT 150-1045 -Control
Unknown 751	532.2654	6.6327	18.6	0.0001	0.0005	1045- BIT 150-1045 -Control
Unknown 752	345.0538	3.2401	10.8	0.001	0.0029	1045- BIT 150-1045 -Control
Unknown 753	681.5045	7.5735	7.61	0.004111	0.0085	1045- MIT 25-1045- BIT 150
Unknown 755	111.0142	0.6237	16.8	0.0001	0.0007	1045- CIT 4-1045 -Control
Unknown 758	610.2853	2.6931	9.85	0.0015	0.0039	1045- CIT 4-1045 -Control
Unknown 76	531.3679	6.6224	27.9	0	0.0001	1045- BIT 150-1045 -Control
Unknown 760	333.2103	6.3102	21.1	0	0.0003	1045- BIT 150-1045 -Control
Unknown 761	701.563	6.0223	10.1	0.0013	0.0036	1045- CIT 4-1045- BIT 150
Unknown 762	523.1512	2.2668	6.4	0.0077723	0.0138	1045- CIT 4-1045- BIT 150
Unknown 763	591.2909	6.687	23.1	0	0.0002	1045- BIT 150-1045 -Control
Unknown 764	336.2236	7.0172	10.5	0.0011	0.0031	1045- BIT 150-1045 -Control
Unknown 766	395.2327	6.7428	11.7	0.0007	0.0022	1045- BIT 150-1045 -Control
Unknown 767	703.3369	7.1695	10.7	0.001	0.003	1045- BIT 150-1045 -Control
Unknown 768	799.6102	6.7141	10.2	0.0013	0.0034	1045- MIT 25-1045 -Control
Unknown 769	295.2275	5.3479	9.68	0.0016	0.0041	1045- CIT 4-1045- BIT 150
Unknown 77	215.1651	7.8986	20.1	0.0001	0.0004	1045- BIT 150-1045 -Control
Unknown 770	783.6351	6.6348	6.98	0.0056695	0.0107	1045- CIT 4-1045- BIT 150
Unknown 771	411.3559	7.494	17.6	0.0001	0.0006	1045- BIT 150-1045 -Control
Unknown 772	528.1291	0.8156	6.5	0.0073556	0.0132	1045- CIT 4-1045 -Control
Unknown 773	377.2378	5.3975	12.9	0.0005	0.0016	1045- BIT 150-1045 -Control
Unknown 776	557.2025	6.049	19.9	0.0001	0.0004	1045- BIT 150-1045 -Control
Unknown 777	477.1737	0.8861	5.58	0.012441	0.0204	1045- CIT 4-1045- BIT 150
Unknown 779	589.4327	6.643	15	0.0002	0.001	1045- BIT 150-1045 -Control
Unknown 78	511.4729	7.127	7.96	0.0034665	0.0075	1045- MIT 25-1045 -Control
Unknown 781	537.4863	7.2131	13.9	0.0003	0.0013	1045- BIT 150-1045 -Control
Unknown 782	410.033	0.8068	4.01	0.03427	0.0485	
Unknown 784	412.209	0.9038	6.13	0.0090577	0.0156	1045- CIT 4-1045 -Control
Unknown 785	411.3042	7.7655	5.48	0.013174	0.0214	
Unknown 786	541.3607	7.9225	31.4	0	0.0001	1045- BIT 150-1045 -Control
Unknown 787	639.4575	7.0369	22.4	0	0.0003	1045- BIT 150-1045 -Control
Unknown 788	581.2425	4.3955	29.2	0	0.0001	1045- BIT 150-1045 -Control
Unknown 79	532.2656	6.6945	19.2	0.0001	0.0004	1045- BIT 150-1045 -Control
Unknown 790	650.3447	2.9799	4.93	0.018561	0.0283	
Unknown 791	523.305	6.9151	4.27	0.028719	0.0415	
Unknown 792	213.0921	3.5923	14.2	0.0003	0.0012	1045- BIT 150-1045 -Control
Unknown 794	291.1908	4.9525	15.8	0.0002	0.0008	1045- BIT 150-1045 -Control
Unknown 795	449.577	0.556	57.7	0	0	1045- BIT 150-1045 -Control
Unknown 796	625.3349	2.5862	7.19	0.0050816	0.0098	1045- CIT 4-1045 -Control
Unknown 798	605.5196	8.8259	8.38	0.0028387	0.0065	1045- CIT 4-1045- BIT 150
Unknown 799	245.0928	3.2508	7.75	0.0038353	0.0081	1045- CIT 4-1045- BIT 150

Unknown 8	328.0736	0.8917	24.5	0	0.0002	1045- BIT 150-1045 -Control
Unknown 80	164.0916	0.9084	7.05	0.005471	0.0104	1045- CIT 4-1045 -Control
Unknown 800	672.3642	2.2442	7.58	0.0041762	0.0086	1045- CIT 4-1045- BIT 150
Unknown 801	804.5578	4.376	9.25	0.0019	0.0047	1045- CIT 4-1045- BIT 150
Unknown 802	221.0898	0.8526	7.8	0.0037447	0.008	1045- CIT 4-1045 -Control
Unknown 803	345.1719	0.7901	4.33	0.027639	0.0401	
Unknown 804	826.6078	8.9207	15.7	0.0002	0.0008	1045- BIT 150-1045 -Control
Unknown 805	618.421	7.9788	28.2	0	0.0001	1045- BIT 150-1045 -Control
Unknown 806	295.112	4.5375	11.6	0.0007	0.0023	1045- BIT 150-1045 -Control
Unknown 807	195.1386	4.8949	8.58	0.0025828	0.006	1045- CIT 4-1045- BIT 150
Unknown 808	213.135	3.5733	54.2	0	0	1045- BIT 150-1045 -Control
Unknown 809	579.3649	8.2283	26.6	0	0.0002	1045- BIT 150-1045 -Control
Unknown 81	483.2748	6.4662	35.7	0	0.0001	1045- BIT 150-1045 -Control
Unknown 810	639.5561	7.6718	9.51	0.0017	0.0044	1045- CIT 4-1045- BIT 150
Unknown 813	294.1302	0.9072	5.4	0.013883	0.0224	
Unknown 814	566.8223	2.5634	28.2	0	0.0001	1045- BIT 150-1045 -Control
Unknown 817	657.3866	8.2451	13.4	0.0004	0.0014	1045- CIT 4-1045- BIT 150
Unknown 818	556.3138	2.4526	11.7	0.0007	0.0022	1045- CIT 4-1045 -Control
Unknown 819	617.4757	5.6265	22.3	0	0.0003	1045- BIT 150-1045 -Control
Unknown 82	529.3357	6.3473	4.68	0.021774	0.0326	
Unknown 820	492.0371	0.8075	7.98	0.0034281	0.0075	1045- BIT 150-1045 -Control
Unknown 822	543.3833	8.226	29	0	0.0001	1045- BIT 150-1045 -Control
Unknown 823	405.1945	4.148	12.4	0.0006	0.0018	1045- BIT 150-1045 -Control
Unknown 824	355.2031	5.6332	14.4	0.0003	0.0011	1045- BIT 150-1045 -Control
Unknown 825	462.3194	7.0911	7.65	0.0040323	0.0084	1045- BIT 150-1045 -Control
Unknown 827	604.0763	0.6668	5.54	0.012773	0.0208	
Unknown 828	208.0906	3.5947	12.5	0.0005	0.0018	1045- BIT 150-1045 -Control
Unknown 830	277.1229	7.9175	12.3	0.0006	0.0019	1045- CIT 4-1045- BIT 150
Unknown 832	667.5446	5.3852	12.3	0.0006	0.0019	1045- CIT 4-1045- BIT 150
Unknown 834	212.09	0.8028	25.4	0	0.0002	1045- BIT 150-1045 -Control
Unknown 835	661.0824	0.644	48.9	0	0	1045- BIT 150-1045 -Control
Unknown 837	213.1493	4.6555	9.16	0.002	0.0049	1045- CIT 4-1045- BIT 150
Unknown 838	669.3945	3.4581	5.61	0.012186	0.0201	1045- MIT 25-1045 -Control
Unknown 839	297.1657	3.9037	17.1	0.0001	0.0006	1045- BIT 150-1045 -Control
Unknown 84	374.2387	5.4748	4.23	0.029404	0.0424	
Unknown 840	317.2097	5.4939	6.21	0.0086557	0.0151	1045- BIT 150-1045 -Control
Unknown 841	647.2796	6.5529	40.7	0	0	1045- BIT 150-1045 -Control
Unknown 842	573.2516	0.9775	4.29	0.028336	0.0411	
Unknown 843	227.0647	4.7624	18.9	0.0001	0.0005	1045- BIT 150-1045 -Control
Unknown 844	708.3509	2.2291	11.7	0.0007	0.0022	1045- CIT 4-1045 -Control
Unknown 845	510.416	8.3708	12.8	0.0005	0.0017	1045- BIT 150-1045 -Control
Unknown 848	359.2888	5.1934	4.16	0.030989	0.0443	
Unknown 849	577.3498	7.924	33.3	0	0.0001	1045- BIT 150-1045 -Control
Unknown 85	461.26	5.5971	11.1	0.0009	0.0026	1045- BIT 150-1045 -Control
Unknown 850	309.2796	7.7384	32.4	0	0.0001	1045- BIT 150-1045 -Control
Unknown 851	383.1769	5.8996	6.99	0.0056494	0.0107	1045- CIT 4-1045- BIT 150
Unknown 852	744.5325	4.9357	12	0.0006	0.0021	1045- BIT 150-1045 -Control
Unknown 853	337.2011	5.1836	5.03	0.017484	0.0272	

Unknown 854	343.1942	1.1142	4.98	0.017983	0.0276	
Unknown 856	610.0834	0.6751	9.71	0.0016	0.0041	1045- CIT 4-1045 -Control
Unknown 858	469.2626	6.1357	35.6	0	0.0001	1045- BIT 150-1045 -Control
Unknown 860	494.2877	5.8032	4.54	0.023909	0.0354	
Unknown 862	385.1379	2.7975	7.83	0.0036872	0.0079	1045- CIT 4-1045- BIT 150
Unknown 863	223.1701	5.5085	15.1	0.0002	0.001	1045- BIT 150-1045 -Control
Unknown 864	807.9489	3.7081	10.5	0.0011	0.0032	1045- CIT 4-1045 -Control
Unknown 865	605.5083	6.023	41.2	0	0	1045- BIT 150-1045 -Control
Unknown 866	440.3281	7.8693	14.6	0.0003	0.0011	1045- BIT 150-1045 -Control
Unknown 870	468.0108	6.4965	5.45	0.01343	0.0217	
Unknown 871	211.0521	3.5295	19.8	0.0001	0.0004	1045- BIT 150-1045 -Control
Unknown 873	687.4125	6.638	42	0	0	1045- BIT 150-1045 -Control
Unknown 874	454.2666	1.0946	10	0.0014	0.0037	1045- CIT 4-1045 -Control
Unknown 875	569.0515	0.6329	20.4	0.0001	0.0004	1045- BIT 150-1045 -Control
Unknown 878	436.2329	0.8018	5.68	0.01171	0.0194	1045- CIT 4-1045 -Control
Unknown 88	323.1811	4.3714	13.7	0.0004	0.0013	1045- BIT 150-1045 -Control
Unknown 882	309.1385	0.9475	5.46	0.013373	0.0216	
Unknown 883	477.3846	8.2205	4.81	0.020109	0.0304	
Unknown 885	305.1297	2.9708	4.67	0.022011	0.0329	
Unknown 886	456.3454	7.5879	34.6	0	0.0001	1045- BIT 150-1045 -Control
Unknown 887	269.212	5.8401	9.68	0.0016	0.0041	1045- MIT 25-1045 -Control
Unknown 89	283.152	0.8062	6.35	0.0079716	0.0141	1045- CIT 4-1045 -Control
Unknown 890	735.4811	8.1509	43.3	0	0	1045- BIT 150-1045 -Control
Unknown 892	359.1513	0.6713	9.02	0.0021	0.0051	1045- CIT 4-1045 -Control
Unknown 893	321.1945	5.9498	6.65	0.0067889	0.0123	1045- BIT 150-1045 -Control
Unknown 894	577.2778	6.3594	89.4	0	0	1045- BIT 150-1045 -Control
Unknown 896	311.2201	4.1049	28.2	0	0.0001	1045- BIT 150-1045 -Control
Unknown 897	600.3578	0.9475	18.3	0.0001	0.0005	1045- MIT 25-1045 -Control
Unknown 899	621.3344	7.1404	8.69	0.0024543	0.0058	1045- CIT 4-1045- BIT 150
Unknown 9	533.4548	7.1269	4.53	0.024081	0.0355	
Unknown 902	243.0613	0.6445	9.95	0.0014	0.0037	1045- CIT 4-1045 -Control
Unknown 903	626.2766	0.8883	11.5	0.0008	0.0023	1045- CIT 4-1045 -Control
Unknown 904	608.2694	1.0061	4.9	0.01897	0.0289	
Unknown 905	217.003	3.9258	6.83	0.0061457	0.0114	1045- CIT 4-1045- BIT 150
Unknown 906	381.1972	1.0257	4.66	0.02213	0.0331	
Unknown 907	347.1547	2.7751	28.6	0	0.0001	1045- BIT 150-1045 -Control
Unknown 909	380.1722	0.8798	7.61	0.0041182	0.0085	1045- CIT 4-1045 -Control
Unknown 91	295.1548	4.0248	9.67	0.0016	0.0041	1045- BIT 150-1045 -Control
Unknown 910	707.4497	7.8959	11.1	0.0009	0.0026	1045- CIT 4-1045 -Control
Unknown 912	487.2134	4.6256	4.81	0.02004	0.0303	
Unknown 914	511.2156	0.9368	5.55	0.012698	0.0208	
Unknown 918	575.3075	2.3785	18.2	0.0001	0.0005	1045- CIT 4-1045 -Control
Unknown 92	261.0954	1.0204	6.64	0.0068119	0.0123	1045- CIT 4-1045 -Control
Unknown 922	468.0107	5.3963	5.02	0.017562	0.0272	
Unknown 923	596.3177	2.4217	14.2	0.0003	0.0012	1045- CIT 4-1045 -Control
Unknown 925	307.264	7.475	8.04	0.0033293	0.0073	1045- CIT 4-1045- BIT 150
Unknown 926	661.472	7.2428	66.2	0	0	1045- BIT 150-1045 -Control
Unknown 927	843.4999	8.5199	10.9	0.001	0.0028	1045- BIT 150-1045 -Control

Unknown 929	351.2153	5.1757	9.39	0.0018	0.0045	1045- CIT 4-1045- BIT 150
Unknown 93	379.2404	5.8463	12.3	0.0006	0.0019	1045- BIT 150-1045 -Control
Unknown 930	522.3371	7.8613	59.8	0	0	1045- BIT 150-1045 -Control
Unknown 931	603.4037	8.2287	29.9	0	0.0001	1045- BIT 150-1045 -Control
Unknown 935	726.4688	6.7039	34.2	0	0.0001	1045- BIT 150-1045 -Control
Unknown 936	383.2071	1.0018	12.3	0.0006	0.0019	1045- CIT 4-1045 -Control
Unknown 937	455.1654	2.9305	17.2	0.0001	0.0006	1045- BIT 150-1045 -Control
Unknown 939	294.0944	0.8043	19.1	0.0001	0.0005	1045- BIT 150-1045 -Control
Unknown 94	640.322	0.8864	6.94	0.0058106	0.0109	1045- CIT 4-1045 -Control
Unknown 940	356.2546	2.5184	15.8	0.0002	0.0008	1045- BIT 150-1045 -Control
Unknown 944	545.4158	5.8794	5.1	0.016716	0.0261	
Unknown 946	433.2197	5.8418	35	0	0.0001	1045- BIT 150-1045 -Control
Unknown 947	549.2014	0.6778	13.8	0.0003	0.0013	1045- CIT 4-1045 -Control
Unknown 949	313.2371	3.6891	8.16	0.0031486	0.0071	1045- BIT 150-1045 -Control
Unknown 951	197.0944	0.8314	8.19	0.0030972	0.007	1045- CIT 4-1045 -Control
Unknown 953	586.2418	2.6915	7.67	0.0039965	0.0084	1045- CIT 4-1045 -Control
Unknown 954	586.3353	2.4077	4.54	0.023873	0.0354	
Unknown 957	467.3236	7.8249	14.3	0.0003	0.0012	1045- BIT 150-1045 -Control
Unknown 959	295.1377	1.2333	5.02	0.017582	0.0272	
Unknown 96	321.2373	5.9998	8.02	0.00337	0.0074	1045- CIT 4-1045- BIT 150
Unknown 960	411.1839	4.0523	10.6	0.0011	0.003	1045- BIT 150-1045 -Control
Unknown 962	277.123	3.3489	6.08	0.0093055	0.0159	1045- CIT 4-1045- BIT 150
Unknown 964	583.1638	0.8343	7.18	0.0051327	0.0099	1045- BIT 150-1045 -Control
Unknown 965	716.5011	4.9412	5.28	0.014885	0.0238	
Unknown 966	656.3475	2.421	13.4	0.0004	0.0014	1045- CIT 4-1045 -Control
Unknown 967	548.2523	0.8377	7.98	0.0034259	0.0075	1045- CIT 4-1045 -Control
Unknown 969	285.0896	0.9646	15.5	0.0002	0.0009	1045- BIT 150-1045 -Control
Unknown 97	532.2654	6.7905	10.1	0.0014	0.0036	1045- BIT 150-1045 -Control
Unknown 970	589.389	8.2222	27.5	0	0.0001	1045- BIT 150-1045 -Control
Unknown 971	410.0827	0.8467	17.3	0.0001	0.0006	1045- BIT 150-1045 -Control
Unknown 972	491.1223	0.7932	9.39	0.0018	0.0045	1045- BIT 150-1045 -Control
Unknown 973	728.4871	8.3019	21.1	0	0.0003	1045- BIT 150-1045 -Control
Unknown 974	638.3171	2.4543	9.44	0.0018	0.0045	1045- CIT 4-1045 -Control
Unknown 975	534.2812	7.0977	15.2	0.0002	0.001	1045- BIT 150-1045 -Control
Unknown 976	324.1817	0.8733	16.1	0.0002	0.0008	1045- BIT 150-1045 -Control
Unknown 977	374.3157	7.4849	4.27	0.028602	0.0414	
Unknown 978	549.1449	0.8095	5.89	0.010346	0.0174	1045- BIT 150-1045 -Control
Unknown 980	555.3763	8.202	14.2	0.0003	0.0012	1045- BIT 150-1045 -Control
Unknown 981	737.3821	2.9774	6.12	0.0091065	0.0157	1045- BIT 150-1045 -Control
Unknown 982	551.5038	7.3539	7.91	0.0035462	0.0076	1045- MIT 25-1045 -Control
Unknown 985	295.1388	0.7773	10.3	0.0012	0.0034	1045- CIT 4-1045 -Control
Unknown 986	620.3945	7.5587	5.26	0.015072	0.024	
Unknown 987	751.4345	2.2348	9.39	0.0018	0.0045	1045- CIT 4-1045 -Control
Unknown 989	325.1987	5.4893	8.33	0.0029085	0.0066	1045- CIT 4-1045- BIT 150
Unknown 99	214.1365	2.2472	6.76	0.0063809	0.0118	1045- CIT 4-1045 -Control
Unknown 990	730.5036	8.7344	13.9	0.0003	0.0013	1045- BIT 150-1045 -Control
Unknown 991	465.2331	1.23	11	0.0009	0.0027	1045- CIT 4-1045 -Control
Unknown 992	405.1936	1.0508	14.6	0.0003	0.0011	1045- CIT 4-1045 -Control

Unknown 993	475.2666	7.59	7.98	0.003437	0.0075	1045- BIT 150-1045 -Control
Unknown 994	467.233	2.6934	20.6	0.0001	0.0004	1045- BIT 150-1045 -Control
Unknown 996	468.1661	0.9101	5.64	0.012021	0.0198	1045- CIT 4-1045- BIT 150
Unknown 997	248.1056	4.4734	22.4	0	0.0003	1045- BIT 150-1045 -Control
Unknown 999	509.1945	0.7996	5.26	0.01512	0.024	

Supplementary Table S7: ANOVA analysis of lipids in biocide treated *P. oleovorans* P4A strain.

Lipid Name	m/z	Retention time	f.value	p.value	FDR	Tukey's HSD
Unknown 1390	660.2531	0.86	17.9	0.0001	0.027	P4A- CMIT 4-P4A- BIT 150
Unknown 1691	922.6007	6.44	17.1	0.0001	0.027	P4A- Control-P4A- BIT 150
Unknown 1387	638.2711	0.85	16.4	0.0002	0.027	P4A- CMIT 4-P4A- BIT 150
Unknown 1190	313.3585	3.64	15	0.0002	0.027	P4A- CMIT 4-P4A- BIT 150
Unknown 1339	137.0466	0.75	14.9	0.0002	0.027	P4A- CMIT 4-P4A- BIT 150
Unknown 1356	297.0405	0.78	14.6	0.0003	0.027	P4A- CMIT 4-P4A- BIT 150
Unknown 1400	439.2905	0.94	14	0.0003	0.028	P4A- CMIT 4-P4A- BIT 150
Unknown 1359	299.0385	0.78	13.4	0.0004	0.028	P4A- CMIT 4-P4A- BIT 150
Unknown 682	104.1068	0.73	13.1	0.0004	0.028	P4A- CMIT 4-P4A- BIT 150
Unknown 1179	792.5759	6.41	12.9	0.0005	0.028	P4A- CMIT 4-P4A- BIT 150
Unknown 788	137.0676	0.73	11.5	0.0008	0.042	P4A- CMIT 4-P4A- BIT 150
Unknown 1393	137.0466	0.75	11.2	0.0009	0.043	P4A- MIT 25-P4A- BIT 150
Unknown 1366	355.0946	0.79	11	0.0009	0.043	P4A- MIT 25-P4A- BIT 150
Unknown 745	250.1158	0.82	10.8	0.0010	0.044	P4A- CMIT 4-P4A- BIT 150
Unknown 741	181.0966	0.87	10.6	0.0011	0.045	P4A- CMIT 4-P4A- BIT 150

Supplementary Table S8: ANOVA analysis of polar metabolites in biocide treated *P. oleovorans* P4A strain.

Polar Metabolites Name	m/z	Retention time	f.value	p.value	FDR	Tukey's HSD
Unknown 574	642.3264	6.3093	96.494	0.0000	0	P4A- CMIT 4-P4A- BIT 150
Unknown 1300	551.2623	6.4565	91.37	0.0000	0	P4A- CMIT 4-P4A- BIT 150
Unknown 719	669.2799	7.9167	88.935	0.0000	0	P4A- CMIT 4-P4A- BIT 150
Unknown 1049	539.3528	7.7799	82.866	0.0000	0	P4A- CMIT 4-P4A- BIT 150
Unknown 296	658.4468	8.6581	72.906	0.0000	0	P4A- CMIT 4-P4A- BIT 150
Unknown 1151	702.4727	8.3862	63.455	0.0000	0	P4A- CMIT 4-P4A- BIT 150
Unknown 1055	604.3998	8.2805	58.914	0.0000	0	P4A- CMIT 4-P4A- BIT 150
Unknown 23	580.3380	7.1149	55.359	0.0000	0.0001	P4A- CMIT 4-P4A- BIT 150
Unknown 851	383.1769	5.8996	53.323	0.0000	0.0001	P4A- CMIT 4-P4A- BIT 150
Unknown 1430	614.2716	6.7033	52.709	0.0000	0.0001	P4A- CMIT 4-P4A- BIT 150
Unknown 698	699.4786	5.6271	51.6	0.0000	0.0001	P4A- CMIT 4-P4A- BIT 150
Unknown 1077	683.0552	0.6304	50.683	0.0000	0.0001	P4A- CMIT 4-P4A- BIT 150
Unknown 1320	704.4848	8.2856	48.458	0.0000	0.0001	P4A- CMIT 4-P4A- BIT 150
Unknown 177	383.1413	2.7755	48.05	0.0000	0.0001	P4A- CMIT 4-P4A- BIT 150
Unknown 1045	483.1940	4.1007	46.646	0.0000	0.0001	P4A- CMIT 4-P4A- BIT 150
Unknown 642	703.4649	8.2555	45.151	0.0000	0.0001	P4A- CMIT 4-P4A- BIT 150
Unknown 1251	704.4844	8.2191	44.877	0.0000	0.0001	P4A- CMIT 4-P4A- BIT 150
Unknown 1410	629.5163	5.8507	44.096	0.0000	0.0001	P4A- CMIT 4-P4A- BIT 150
Unknown 386	577.2779	6.6839	43.145	0.0000	0.0001	P4A- CMIT 4-P4A- BIT 150
Unknown 1027	709.3943	3.1915	43.128	0.0000	0.0001	P4A- CMIT 4-P4A- BIT 150
Unknown 1354	577.2778	6.6150	42.969	0.0000	0.0001	P4A- CMIT 4-P4A- BIT 150
Unknown 112	506.2520	6.4455	41.407	0.0000	0.0001	P4A- CMIT 4-P4A- BIT 150
Unknown 585	464.1156	2.1861	40.928	0.0000	0.0001	P4A- CMIT 4-P4A- BIT 150
Unknown 75	563.2599	6.0325	40.601	0.0000	0.0001	P4A- CMIT 4-P4A- BIT 150
Unknown 32	183.1389	4.4550	39.837	0.0000	0.0001	P4A- CMIT 4-P4A- BIT 150
Unknown 1223	529.4059	4.6607	38.683	0.0000	0.0001	P4A- Control-P4A- BIT 150
Unknown 263	223.1701	5.5585	38.423	0.0000	0.0001	P4A- CMIT 4-P4A- BIT 150
Unknown 231	584.2853	6.2933	38.172	0.0000	0.0001	P4A- CMIT 4-P4A- BIT 150
Unknown 125	396.1999	1.5565	37.55	0.0000	0.0001	P4A- CMIT 4-P4A- BIT 150
Unknown 704	685.2727	5.8912	37.454	0.0000	0.0001	P4A- CMIT 4-P4A- BIT 150
Unknown 376	670.3562	7.0218	36.031	0.0000	0.0001	P4A- CMIT 4-P4A- BIT 150
Unknown 863	223.1701	5.5085	36.023	0.0000	0.0001	P4A- CMIT 4-P4A- BIT 150
Unknown 1233	546.6099	0.5932	35.791	0.0000	0.0001	P4A- CMIT 4-P4A- BIT 150
Unknown 186	307.1885	5.9753	35.658	0.0000	0.0001	P4A- CMIT 4-P4A- BIT 150
Unknown 382	733.4649	7.9738	34.95	0.0000	0.0002	P4A- CMIT 4-P4A- BIT 150
Unknown 729	467.3423	7.5349	34.687	0.0000	0.0002	P4A- CMIT 4-P4A- BIT 150
Unknown 745	565.2756	6.5710	34.014	0.0000	0.0002	P4A- CMIT 4-P4A- BIT 150
Unknown 331	545.3951	6.6344	33.127	0.0000	0.0002	P4A- CMIT 4-P4A- BIT 150
Unknown 632	642.3263	6.3604	32.503	0.0000	0.0002	P4A- CMIT 4-P4A- BIT 150
Unknown 539	630.4129	8.4177	32.34	0.0000	0.0002	P4A- CMIT 4-P4A- BIT 150
Unknown 1434	614.2723	6.6593	32.035	0.0000	0.0002	P4A- CMIT 4-P4A- BIT 150
Unknown 1153	257.1733	3.6642	31.268	0.0000	0.0002	P4A- CMIT 4-P4A- BIT 150
Unknown 215	281.1358	4.0087	30.971	0.0000	0.0002	P4A- MIT 25-P4A- BIT 150
Unknown 1243	647.2780	6.4686	30.869	0.0000	0.0002	P4A- CMIT 4-P4A- BIT 150
Unknown 626	674.4433	8.2729	30.378	0.0000	0.0003	P4A- CMIT 4-P4A- BIT 150
Unknown 656	559.3427	7.5018	29.492	0.0000	0.0003	P4A- CMIT 4-P4A- BIT 150

Unknown 679	582.2693	5.8976	28.422	0.0000	0.0003	P4A- CMIT 4-P4A- BIT 150
Unknown 21	563.2600	5.9730	28.411	0.0000	0.0003	P4A- CMIT 4-P4A- BIT 150
Unknown 1019	575.3731	7.8449	28.376	0.0000	0.0003	P4A- CMIT 4-P4A- BIT 150
Unknown 228	536.2522	7.3418	28.091	0.0000	0.0003	P4A- CMIT 4-P4A- BIT 150
Unknown 405	375.1925	4.0744	28.09	0.0000	0.0003	P4A- CMIT 4-P4A- BIT 150
Unknown 4	565.2760	6.4481	27.73	0.0000	0.0003	P4A- CMIT 4-P4A- BIT 150
Unknown 946	433.2197	5.8418	27.635	0.0000	0.0003	P4A- CMIT 4-P4A- BIT 150
Unknown 1409	601.3882	8.1594	27.091	0.0000	0.0004	P4A- CMIT 4-P4A- BIT 150
Unknown 1025	355.1435	5.0424	26.974	0.0000	0.0004	P4A- CMIT 4-P4A- BIT 150
Unknown 805	618.4210	7.9788	26.636	0.0000	0.0004	P4A- CMIT 4-P4A- BIT 150
Unknown 668	277.1225	7.4917	26.306	0.0000	0.0004	P4A- CMIT 4-P4A- BIT 150
Unknown 38	492.2763	5.6852	25.819	0.0000	0.0004	P4A- CMIT 4-P4A- BIT 150
Unknown 1201	629.5163	6.4142	25.741	0.0000	0.0004	P4A- CMIT 4-P4A- BIT 150
Unknown 199	337.2006	4.9465	25.675	0.0000	0.0004	P4A- CMIT 4-P4A- BIT 150
Unknown 292	549.3128	7.4908	25.646	0.0000	0.0004	P4A- CMIT 4-P4A- BIT 150
Unknown 832	667.5446	5.3852	25.632	0.0000	0.0004	P4A- CMIT 4-P4A- BIT 150
Unknown 516	569.3954	8.2611	25.225	0.0000	0.0005	P4A- CMIT 4-P4A- BIT 150
Unknown 841	647.2796	6.5529	24.785	0.0000	0.0005	P4A- CMIT 4-P4A- BIT 150
Unknown 1219	223.1701	5.6240	24.656	0.0000	0.0005	P4A- CMIT 4-P4A- BIT 150
Unknown 241	785.4942	6.7073	24.585	0.0000	0.0005	P4A- CMIT 4-P4A- BIT 150
Unknown 809	579.3649	8.2283	24.482	0.0000	0.0005	P4A- CMIT 4-P4A- BIT 150
Unknown 35	349.2359	7.0403	23.676	0.0000	0.0006	P4A- CMIT 4-P4A- BIT 150
Unknown 653	424.5792	0.9885	23.502	0.0000	0.0006	P4A- CMIT 4-P4A- BIT 150
Unknown 1171	789.5270	7.9828	23.376	0.0000	0.0006	P4A- CMIT 4-P4A- BIT 150
Unknown 1272	639.5057	5.5245	23.323	0.0000	0.0006	P4A- CMIT 4-P4A- BIT 150
Unknown 502	702.4728	8.4670	23.195	0.0000	0.0006	P4A- CMIT 4-P4A- BIT 150
Unknown 937	455.1654	2.9305	23.194	0.0000	0.0006	P4A- CMIT 4-P4A- BIT 150
Unknown 636	679.5810	6.0231	22.658	0.0000	0.0007	P4A- CMIT 4-P4A- BIT 150
Unknown 1108	277.1221	3.0729	22.414	0.0000	0.0007	P4A- CMIT 4-P4A- BIT 150
Unknown 1028	655.4253	8.4000	22.26	0.0000	0.0007	P4A- CMIT 4-P4A- BIT 150
Unknown 96	321.2373	5.9998	22.025	0.0000	0.0008	P4A- CMIT 4-P4A- BIT 150
Unknown 674	561.3584	7.8480	21.954	0.0000	0.0008	P4A- CMIT 4-P4A- BIT 150
Unknown 788	581.2425	4.3955	21.941	0.0000	0.0008	P4A- CMIT 4-P4A- BIT 150
Unknown 1376	832.5688	8.2816	21.921	0.0000	0.0008	P4A- CMIT 4-P4A- BIT 150
Unknown 1564	559.3421	7.5161	21.722	0.0000	0.0008	P4A- CMIT 4-P4A- BIT 150
Unknown 369	629.3435	7.8543	21.715	0.0000	0.0008	P4A- CMIT 4-P4A- BIT 150
Unknown 323	610.3014	6.4414	21.645	0.0000	0.0008	P4A- CMIT 4-P4A- BIT 150
Unknown 71	582.2693	5.8302	21.521	0.0000	0.0008	P4A- CMIT 4-P4A- BIT 150
Unknown 580	676.4537	8.4500	21.514	0.0000	0.0008	P4A- CMIT 4-P4A- BIT 150
Unknown 1333	810.4913	8.2977	21.51	0.0000	0.0008	P4A- CMIT 4-P4A- BIT 150
Unknown 962	277.1230	3.3489	21.447	0.0000	0.0008	P4A- CMIT 4-P4A- BIT 150
Unknown 665	319.2250	6.4247	21.354	0.0000	0.0008	P4A- CMIT 4-P4A- BIT 150
Unknown 1128	763.5148	7.9645	20.876	0.0000	0.0009	P4A- CMIT 4-P4A- BIT 150
Unknown 1491	650.3978	7.5264	20.638	0.0000	0.0009	P4A- CMIT 4-P4A- BIT 150
Unknown 1299	338.2675	6.0834	20.604	0.0001	0.0009	P4A- CMIT 4-P4A- BIT 150
Unknown 118	649.3783	6.0175	20.456	0.0001	0.0009	P4A- Control-P4A- BIT 150
Unknown 700	761.4926	7.7642	20.378	0.0001	0.0009	P4A- CMIT 4-P4A- BIT 150
Unknown 894	577.2778	6.3594	20.074	0.0001	0.001	P4A- CMIT 4-P4A- BIT 150

Unknown 1580	520.2654	7.0973	19.978	0.0001	0.001	P4A- CMIT 4-P4A- BIT 150
Unknown 1118	152.0394	0.9727	19.862	0.0001	0.001	P4A- CMIT 4-P4A- BIT 150
Unknown 740	341.1195	2.6234	19.746	0.0001	0.001	P4A- CMIT 4-P4A- BIT 150
Unknown 520	389.1952	5.9791	19.639	0.0001	0.0011	P4A- CMIT 4-P4A- BIT 150
Unknown 275	473.3530	5.9080	19.513	0.0001	0.0011	P4A- CMIT 4-P4A- BIT 150
Unknown 422	371.2359	5.9303	19.495	0.0001	0.0011	P4A- CMIT 4-P4A- BIT 150
Unknown 95	493.3452	7.6862	18.921	0.0001	0.0012	P4A- CMIT 4-P4A- BIT 150
Unknown 532	673.2936	6.6851	18.768	0.0001	0.0013	P4A- CMIT 4-P4A- BIT 150
Unknown 555	560.8065	2.8508	18.741	0.0001	0.0013	P4A- CMIT 4-P4A- BIT 150
Unknown 1157	311.2618	5.9893	18.223	0.0001	0.0014	P4A- CMIT 4-P4A- BIT 150
Unknown 330	450.2650	6.7239	18.1	0.0001	0.0015	P4A- CMIT 4-P4A- BIT 150
Unknown 884	277.1225	7.8655	18.072	0.0001	0.0015	P4A- CMIT 4-P4A- BIT 150
Unknown 931	603.4037	8.2287	18.057	0.0001	0.0015	P4A- CMIT 4-P4A- BIT 150
Unknown 1546	209.1279	3.5895	18.043	0.0001	0.0015	P4A- CMIT 4-P4A- BIT 150
Unknown 762	523.1512	2.2668	17.899	0.0001	0.0015	P4A- CMIT 4-P4A- BIT 150
Unknown 1150	455.2389	5.8227	17.877	0.0001	0.0015	P4A- CMIT 4-P4A- BIT 150
Unknown 216	291.1183	3.2502	17.724	0.0001	0.0015	P4A- CMIT 4-P4A- BIT 150
Unknown 521	551.3303	7.8581	17.681	0.0001	0.0016	P4A- CMIT 4-P4A- BIT 150
Unknown 1101	257.1746	3.3595	17.522	0.0001	0.0016	P4A- CMIT 4-P4A- BIT 150
Unknown 445	169.1597	5.1242	17.472	0.0001	0.0016	P4A- CMIT 4-P4A- BIT 150
Unknown 88	323.1811	4.3714	17.463	0.0001	0.0016	P4A- CMIT 4-P4A- BIT 150
Unknown 454	337.2000	5.7605	17.434	0.0001	0.0016	P4A- CMIT 4-P4A- BIT 150
Unknown 572	399.3163	6.3839	17.345	0.0001	0.0016	P4A- CMIT 4-P4A- BIT 150
Unknown 1503	644.3812	2.6169	17.21	0.0001	0.0017	P4A- CMIT 4-P4A- BIT 150
Unknown 786	541.3607	7.9225	17.114	0.0001	0.0017	P4A- CMIT 4-P4A- BIT 150
Unknown 446	141.1283	3.2466	17.096	0.0001	0.0017	P4A- CMIT 4-P4A- BIT 150
Unknown 1089	644.3309	7.7076	17.075	0.0001	0.0017	P4A- CMIT 4-P4A- BIT 150
Unknown 1031	547.4339	6.4329	17	0.0001	0.0017	P4A- CMIT 4-P4A- BIT 150
Unknown 631	361.1617	5.0403	16.956	0.0001	0.0017	P4A- CMIT 4-P4A- BIT 150
Unknown 360	435.5701	0.9788	16.918	0.0001	0.0017	P4A- CMIT 4-P4A- BIT 150
Unknown 1088	602.4171	7.9707	16.918	0.0001	0.0017	P4A- CMIT 4-P4A- BIT 150
Unknown 926	661.4720	7.2428	16.605	0.0001	0.0019	P4A- CMIT 4-P4A- BIT 150
Unknown 503	513.3010	7.5342	16.421	0.0002	0.0019	P4A- CMIT 4-P4A- BIT 150
Unknown 799	245.0928	3.2508	16.356	0.0002	0.002	P4A- CMIT 4-P4A- BIT 150
Unknown 192	605.3099	7.0160	16.199	0.0002	0.002	P4A- CMIT 4-P4A- BIT 150
Unknown 865	605.5083	6.0230	16.181	0.0002	0.002	P4A- CMIT 4-P4A- BIT 150
Unknown 476	689.4413	8.1207	16.168	0.0002	0.002	P4A- CMIT 4-P4A- BIT 150
Unknown 830	277.1229	7.9175	16.162	0.0002	0.002	P4A- CMIT 4-P4A- BIT 150
Unknown 761	701.5630	6.0223	16.159	0.0002	0.002	P4A- CMIT 4-P4A- BIT 150
Unknown 655	635.2606	5.1245	15.986	0.0002	0.0021	P4A- CMIT 4-P4A- BIT 150
Unknown 1053	673.2936	6.6127	15.971	0.0002	0.0021	P4A- CMIT 4-P4A- BIT 150
Unknown 450	279.1569	5.0481	15.939	0.0002	0.0021	P4A- CMIT 4-P4A- BIT 150
Unknown 646	607.3153	6.9659	15.807	0.0002	0.0022	P4A- CMIT 4-P4A- BIT 150
Unknown 862	385.1379	2.7975	15.741	0.0002	0.0022	P4A- CMIT 4-P4A- BIT 150
Unknown 350	459.2696	7.5759	15.698	0.0002	0.0022	P4A- CMIT 4-P4A- BIT 150
Unknown 733	799.4755	8.3093	15.658	0.0002	0.0022	P4A- CMIT 4-P4A- BIT 150
Unknown 295	263.1631	4.9644	15.584	0.0002	0.0022	P4A- CMIT 4-P4A- BIT 150
Unknown 276	243.1963	4.6443	15.329	0.0002	0.0024	P4A- CMIT 4-P4A- BIT 150

Unknown 1512	535.6167	0.5587	15.152	0.0002	0.0025	P4A- CMIT 4-P4A- BIT 150
Unknown 1276	655.3952	7.8910	15.032	0.0002	0.0026	P4A- CMIT 4-P4A- BIT 150
Unknown 1059	397.2527	3.2486	14.969	0.0002	0.0026	P4A- CMIT 4-P4A- BIT 150
Unknown 243	215.1650	3.8734	14.517	0.0003	0.003	P4A- CMIT 4-P4A- BIT 150
Unknown 544	573.3582	7.4955	14.508	0.0003	0.003	P4A- CMIT 4-P4A- BIT 150
Unknown 1099	561.2529	6.7660	14.405	0.0003	0.0031	P4A- CMIT 4-P4A- BIT 150
Unknown 77	215.1651	7.8986	14.349	0.0003	0.0031	P4A- CMIT 4-P4A- BIT 150
Unknown 839	297.1657	3.9037	13.952	0.0003	0.0035	P4A- CMIT 4-P4A- BIT 150
Unknown 1522	845.2207	4.7835	13.733	0.0003	0.0038	P4A- CMIT 4-P4A- BIT 150
Unknown 791	523.3050	6.9151	13.597	0.0004	0.0039	P4A- CMIT 4-P4A- BIT 150
Unknown 615	776.5807	7.2366	13.578	0.0004	0.0039	P4A- CMIT 4-P4A- BIT 150
Unknown 649	636.2852	7.3364	13.555	0.0004	0.0039	P4A- CMIT 4-P4A- BIT 150
Unknown 157	735.4669	6.9414	13.504	0.0004	0.0039	P4A- CMIT 4-P4A- BIT 150
Unknown 770	783.6351	6.6348	13.489	0.0004	0.0039	P4A- CMIT 4-P4A- BIT 150
Unknown 559	191.1233	4.0170	13.394	0.0004	0.004	P4A- MIT 25-P4A- BIT 150
Unknown 1498	619.3194	6.9835	13.392	0.0004	0.004	P4A- CMIT 4-P4A- BIT 150
Unknown 496	675.2429	2.7503	13.27	0.0004	0.0042	P4A- CMIT 4-P4A- BIT 150
Unknown 126	307.1882	5.9080	13.253	0.0004	0.0042	P4A- CMIT 4-P4A- BIT 150
Unknown 43	417.2209	4.6315	13.184	0.0004	0.0042	P4A- Control-P4A- BIT 150
Unknown 255	714.4932	8.8577	13.15	0.0004	0.0043	P4A- CMIT 4-P4A- BIT 150
Unknown 421	481.2603	6.0611	13.067	0.0004	0.0044	P4A- CMIT 4-P4A- BIT 150
Unknown 316	227.1650	4.4826	12.874	0.0005	0.0046	P4A- CMIT 4-P4A- BIT 150
Unknown 577	645.2632	5.9740	12.818	0.0005	0.0047	P4A- CMIT 4-P4A- BIT 150
Unknown 475	648.4274	8.1787	12.697	0.0005	0.0049	P4A- CMIT 4-P4A- BIT 150
Unknown 1074	677.4415	8.1787	12.581	0.0005	0.005	P4A- Control-P4A- BIT 150
Unknown 849	577.3498	7.9240	12.43	0.0005	0.0053	P4A- CMIT 4-P4A- BIT 150
Unknown 480	507.4872	8.4261	12.224	0.0006	0.0057	P4A- CMIT 4-P4A- BIT 150
Unknown 1229	709.5554	4.3475	12.155	0.0006	0.0058	P4A- CMIT 4-P4A- BIT 150
Unknown 810	639.5561	7.6718	12.071	0.0006	0.0059	P4A- CMIT 4-P4A- BIT 150
Unknown 1280	620.3093	7.3499	12.054	0.0006	0.0059	P4A- CMIT 4-P4A- BIT 150
Unknown 129	384.2547	6.4283	12.04	0.0006	0.0059	P4A- CMIT 4-P4A- BIT 150
Unknown 1294	576.2940	6.7994	11.778	0.0007	0.0065	P4A- CMIT 4-P4A- BIT 150
Unknown 167	756.5158	7.2387	11.554	0.0007	0.007	P4A- CMIT 4-P4A- BIT 150
Unknown 807	195.1386	4.8949	11.449	0.0008	0.0072	P4A- MIT 25-P4A- BIT 150
Unknown 471	500.2756	7.3473	11.335	0.0008	0.0075	P4A- CMIT 4-P4A- BIT 150
Unknown 163	576.2853	7.3390	11.215	0.0009	0.0078	P4A- CMIT 4-P4A- BIT 150
Unknown 1576	266.1340	3.4170	11.131	0.0009	0.008	P4A- CMIT 4-P4A- BIT 150
Unknown 365	552.3306	6.6786	11.075	0.0009	0.0082	P4A- CMIT 4-P4A- BIT 150
Unknown 1499	614.2681	7.3394	11.014	0.0009	0.0083	P4A- CMIT 4-P4A- BIT 150
Unknown 1478	453.1456	4.0138	10.76	0.0010	0.0092	P4A- MIT 25-P4A- BIT 150
Unknown 822	543.3833	8.2260	10.745	0.0010	0.0092	P4A- CMIT 4-P4A- BIT 150
Unknown 487	584.4889	5.8508	10.655	0.0011	0.0094	P4A- CMIT 4-P4A- BIT 150
Unknown 780	561.4131	4.9482	10.54	0.0011	0.0098	P4A- CMIT 4-P4A- BIT 150
Unknown 58	534.2811	7.2100	10.477	0.0011	0.01	P4A- CMIT 4-P4A- BIT 150
Unknown 322	451.3055	6.8403	10.437	0.0012	0.0101	P4A- CMIT 4-P4A- BIT 150
Unknown 259	605.4099	6.6391	10.415	0.0012	0.0102	P4A- CMIT 4-P4A- BIT 150
Unknown 627	397.1583	3.3409	10.358	0.0012	0.0104	P4A- CMIT 4-P4A- BIT 150
Unknown 1395	425.2729	2.0904	10.335	0.0012	0.0104	P4A- CMIT 4-P4A- BIT 150

Unknown 483	489.2346	2.2137	10.333	0.0012	0.0104	P4A- MIT 25-P4A- BIT 150
Unknown 1110	615.4056	8.2450	10.262	0.0012	0.0106	P4A- CMIT 4-P4A- BIT 150
Unknown 666	580.3618	7.3392	10.245	0.0013	0.0106	P4A- CMIT 4-P4A- BIT 150
Unknown 30	371.1422	4.0174	10.239	0.0013	0.0106	P4A- MIT 25-P4A- BIT 150
Unknown 106	549.2443	5.9730	10.195	0.0013	0.0107	P4A- CMIT 4-P4A- BIT 150
Unknown 1386	758.5330	8.4680	10.114	0.0013	0.011	P4A- CMIT 4-P4A- BIT 150
Unknown 763	591.2909	6.6870	10.107	0.0013	0.011	P4A- CMIT 4-P4A- BIT 150
Unknown 604	409.1739	4.1502	10.051	0.0014	0.0112	P4A- CMIT 4-P4A- BIT 150
Unknown 652	699.4787	5.8534	9.9012	0.0014	0.0119	P4A- CMIT 4-P4A- BIT 150
Unknown 724	632.4307	8.5818	9.8539	0.0015	0.0121	P4A- MIT 25-P4A- BIT 150
Unknown 1465	834.5886	8.4653	9.8005	0.0015	0.0122	P4A- CMIT 4-P4A- BIT 150
Unknown 909	380.1722	0.8798	9.7979	0.0015	0.0122	P4A- MIT 25-P4A- BIT 150
Unknown 470	513.3305	7.5065	9.7093	0.0016	0.0126	P4A- CMIT 4-P4A- BIT 150
Unknown 159	724.3018	7.3482	9.6886	0.0016	0.0127	P4A- CMIT 4-P4A- BIT 150
Unknown 1561	489.2709	1.8381	9.5569	0.0017	0.0134	P4A- MIT 25-P4A- BIT 150
Unknown 336	629.3119	1.0729	9.4319	0.0018	0.014	P4A- CMIT 4-P4A- BIT 150
Unknown 645	152.0318	0.8357	9.3762	0.0018	0.0143	P4A- MIT 25-P4A- BIT 150
Unknown 944	545.4158	5.8794	9.3548	0.0018	0.0144	P4A- CMIT 4-P4A- BIT 150
Unknown 1014	728.4874	8.3766	9.3157	0.0019	0.0145	P4A- CMIT 4-P4A- BIT 150
Unknown 1357	474.2650	7.0495	9.2876	0.0019	0.0146	P4A- CMIT 4-P4A- BIT 150
Unknown 650	550.3577	3.1316	9.2463	0.0019	0.0148	P4A- CMIT 4-P4A- BIT 150
Unknown 535	585.3683	7.7909	9.2399	0.0019	0.0148	P4A- CMIT 4-P4A- BIT 150
Unknown 965	716.5011	4.9412	9.2076	0.0019	0.015	P4A- MIT 25-P4A- BIT 150
Unknown 711	637.4046	7.9579	9.108	0.0020	0.0156	P4A- Control-P4A- BIT 150
Unknown 908	775.4632	5.6299	9.0279	0.0021	0.0161	P4A- CMIT 4-P4A- BIT 150
Unknown 980	555.3763	8.2020	9.0003	0.0021	0.0162	P4A- CMIT 4-P4A- BIT 150
Unknown 1072	444.3464	8.0755	8.9864	0.0021	0.0162	P4A- Control-P4A- CMIT 4
Unknown 794	291.1908	4.9525	8.9291	0.0022	0.0165	P4A- CMIT 4-P4A- BIT 150
Unknown 1422	433.3464	8.8024	8.9276	0.0022	0.0165	P4A- CMIT 4-P4A- BIT 150
Unknown 1379	291.1908	5.1791	8.8773	0.0023	0.0168	P4A- CMIT 4-P4A- BIT 150
Unknown 172	241.1808	4.1449	8.8489	0.0023	0.0169	P4A- CMIT 4-P4A- BIT 150
Unknown 1574	452.2966	7.4996	8.8405	0.0023	0.0169	P4A- CMIT 4-P4A- BIT 150
Unknown 750	617.4758	5.8523	8.8217	0.0023	0.0169	P4A- CMIT 4-P4A- BIT 150
Unknown 239	267.1960	4.7514	8.8123	0.0023	0.0169	P4A- CMIT 4-P4A- BIT 150
Unknown 326	467.1937	6.4489	8.8113	0.0023	0.0169	P4A- MIT 25-P4A- BIT 150
Unknown 890	735.4811	8.1509	8.776	0.0024	0.0171	P4A- CMIT 4-P4A- BIT 150
Unknown 10	677.4178	6.5649	8.7431	0.0024	0.0173	P4A- Control-P4A- BIT 150
Unknown 200	663.4168	8.0376	8.705	0.0024	0.0175	P4A- MIT 25-P4A- BIT 150
Unknown 132	197.1545	5.1221	8.6832	0.0025	0.0176	P4A- CMIT 4-P4A- BIT 150
Unknown 466	788.3985	2.6827	8.5209	0.0027	0.0189	P4A- MIT 25-P4A- BIT 150
Unknown 1531	543.6028	0.5712	8.4842	0.0027	0.0191	P4A- CMIT 4-P4A- BIT 150
Unknown 664	600.2650	2.1902	8.4276	0.0028	0.0196	P4A- MIT 25-P4A- BIT 150
Unknown 1052	812.5070	8.7347	8.3785	0.0028	0.0199	P4A- CMIT 4-P4A- BIT 150
Unknown 300	733.4630	6.4449	8.3722	0.0028	0.0199	P4A- CMIT 4-P4A- BIT 150
Unknown 307	663.2897	5.8769	8.3571	0.0029	0.02	P4A- CMIT 4-P4A- BIT 150
Unknown 1175	249.1037	4.1170	8.3321	0.0029	0.02	P4A- CMIT 4-P4A- BIT 150
Unknown 960	411.1839	4.0523	8.3277	0.0029	0.02	P4A- MIT 25-P4A- BIT 150
Unknown 137	424.3419	8.3116	8.3229	0.0029	0.02	P4A- CMIT 4-P4A- BIT 150

Unknown 1417	534.2520	1.0333	8.3052	0.0029	0.0201	P4A- MIT 25-P4A- BIT 150
Unknown 1114	552.3305	6.6117	8.2981	0.0029	0.0201	P4A- CMIT 4-P4A- BIT 150
Unknown 1180	554.3471	7.0927	8.2948	0.0030	0.0201	P4A- CMIT 4-P4A- BIT 150
Unknown 806	295.1120	4.5375	8.2587	0.0030	0.0203	P4A- Control-P4A- BIT 150
Unknown 1536	446.1368	0.6607	8.2112	0.0031	0.0207	P4A- MIT 25-P4A- BIT 150
Unknown 335	153.1284	4.0074	8.1756	0.0031	0.021	P4A- MIT 25-P4A- BIT 150
Unknown 1119	439.3009	6.4335	8.1469	0.0032	0.0212	P4A- CMIT 4-P4A- BIT 150
Unknown 1433	616.9146	0.5884	8.1256	0.0032	0.0213	P4A- CMIT 4-P4A- BIT 150
Unknown 1501	403.2982	5.9546	8.0879	0.0033	0.0216	P4A- CMIT 4-P4A- BIT 150
Unknown 1400	599.2284	0.9260	8.0812	0.0033	0.0216	P4A- MIT 25-P4A- BIT 150
Unknown 609	546.2809	7.3479	8.0474	0.0033	0.0218	P4A- CMIT 4-P4A- BIT 150
Unknown 31	377.1535	4.0179	8.0275	0.0034	0.022	P4A- MIT 25-P4A- BIT 150
Unknown 873	687.4125	6.6380	7.9825	0.0034	0.0223	P4A- CMIT 4-P4A- BIT 150
Unknown 1164	435.2414	6.2484	7.9824	0.0034	0.0223	P4A- MIT 25-P4A- BIT 150
Unknown 1348	154.0590	0.8559	7.9632	0.0035	0.0224	P4A- CMIT 4-P4A- BIT 150
Unknown 779	589.4327	6.6430	7.8979	0.0036	0.023	P4A- CMIT 4-P4A- BIT 150
Unknown 1124	730.5001	7.1056	7.8386	0.0037	0.0236	P4A- CMIT 4-P4A- BIT 150
Unknown 767	703.3369	7.1695	7.8254	0.0037	0.0237	P4A- CMIT 4-P4A- BIT 150
Unknown 1010	365.1922	4.9725	7.8147	0.0037	0.0237	P4A- CMIT 4-P4A- BIT 150
Unknown 500	549.3278	6.9230	7.7875	0.0038	0.0239	P4A- CMIT 4-P4A- BIT 150
Unknown 1032	595.4938	5.8486	7.7526	0.0038	0.0242	P4A- CMIT 4-P4A- BIT 150
Unknown 996	468.1661	0.9101	7.6621	0.0040	0.0251	P4A- MIT 25-P4A- BIT 150
Unknown 1271	390.1269	1.9062	7.6559	0.0040	0.0251	P4A- CMIT 4-P4A- BIT 150
Unknown 1109	485.2659	1.1220	7.6544	0.0040	0.0251	P4A- MIT 25-P4A- BIT 150
Unknown 1172	655.2440	0.6837	7.6523	0.0040	0.0251	P4A- MIT 25-P4A- BIT 150
Unknown 1530	752.4341	2.9816	7.4885	0.0044	0.0271	P4A- MIT 25-P4A- BIT 150
Unknown 935	726.4688	6.7039	7.4673	0.0044	0.0273	P4A- MIT 25-P4A- BIT 150
Unknown 99	214.1365	2.2472	7.4215	0.0045	0.0278	P4A- MIT 25-P4A- BIT 150
Unknown 281	351.2136	5.7322	7.3626	0.0047	0.0286	P4A- CMIT 4-P4A- BIT 150
Unknown 1007	567.2863	6.5022	7.35	0.0047	0.0287	P4A- CMIT 4-P4A- BIT 150
Unknown 658	427.2085	4.9617	7.3268	0.0047	0.0289	P4A- MIT 25-P4A- BIT 150
Unknown 527	732.3867	2.2200	7.3143	0.0048	0.029	P4A- MIT 25-P4A- BIT 150
Unknown 973	728.4871	8.3019	7.3034	0.0048	0.029	P4A- CMIT 4-P4A- BIT 150
Unknown 613	546.2810	7.0052	7.2942	0.0048	0.0291	P4A- CMIT 4-P4A- BIT 150
Unknown 1500	367.2043	4.9667	7.2357	0.0050	0.0298	P4A- MIT 25-P4A- BIT 150
Unknown 314	591.2905	6.5969	7.2208	0.0050	0.0298	P4A- CMIT 4-P4A- BIT 150
Unknown 1197	201.1494	3.8284	7.2184	0.0050	0.0298	P4A- CMIT 4-P4A- BIT 150
Unknown 826	599.5239	5.9943	7.2119	0.0050	0.0298	P4A- Control-P4A- BIT 150
Unknown 1121	127.1093	4.5761	7.2113	0.0050	0.0298	P4A- MIT 25-P4A- BIT 150
Unknown 1211	261.1178	1.2622	7.1782	0.0051	0.0302	P4A- CMIT 4-P4A- BIT 150
Unknown 34	141.1283	3.5857	7.1178	0.0053	0.031	P4A- CMIT 4-P4A- BIT 150
Unknown 657	634.3243	2.8116	7.1153	0.0053	0.031	P4A- CMIT 4-P4A- BIT 150
Unknown 453	365.2315	6.4199	7.0944	0.0054	0.0312	P4A- CMIT 4-P4A- BIT 150
Unknown 623	405.1880	4.9678	7.0861	0.0054	0.0312	P4A- MIT 25-P4A- BIT 150
Unknown 760	333.2103	6.3102	6.9843	0.0057	0.0328	P4A- Control-P4A- BIT 150
Unknown 467	687.4139	6.7169	6.9722	0.0057	0.0329	P4A- MIT 25-P4A- BIT 150
Unknown 1508	549.1986	6.4492	6.9673	0.0057	0.0329	P4A- CMIT 4-P4A- BIT 150
Unknown 1525	654.2285	0.8222	6.934	0.0058	0.0333	P4A- MIT 25-P4A- BIT 150

Unknown 515	495.2727	6.3510	6.8987	0.0059	0.0338	P4A- CMIT 4-P4A- BIT 150
Unknown 929	351.2153	5.1757	6.8725	0.0060	0.0342	P4A- MIT 25-P4A- BIT 150
Unknown 1431	787.5115	8.0285	6.8355	0.0061	0.0347	P4A- MIT 25-P4A- BIT 150
Unknown 1575	319.2307	6.8439	6.7767	0.0063	0.0357	P4A- Control-P4A- BIT 150
Unknown 528	527.3503	7.7672	6.7134	0.0065	0.0368	P4A- CMIT 4-P4A- BIT 150
Unknown 702	634.2352	0.8788	6.7067	0.0066	0.0368	P4A- MIT 25-P4A- BIT 150
Unknown 1559	235.1177	1.7335	6.6916	0.0066	0.037	P4A- MIT 25-P4A- BIT 150
Unknown 359	685.4629	5.6313	6.6633	0.0067	0.0375	P4A- CMIT 4-P4A- BIT 150
Unknown 1337	685.3642	2.1684	6.6569	0.0067	0.0375	P4A- MIT 25-P4A- BIT 150
Unknown 15	805.6300	6.6361	6.6238	0.0069	0.038	P4A- CMIT 4-P4A- BIT 150
Unknown 185	592.2990	1.0915	6.613	0.0069	0.0381	P4A- MIT 25-P4A- BIT 150
Unknown 1343	812.5082	7.0941	6.6056	0.0069	0.0381	P4A- MIT 25-P4A- BIT 150
Unknown 789	253.2562	6.7023	6.5848	0.0070	0.0384	P4A- CMIT 4-P4A- BIT 150
Unknown 191	389.1973	5.8741	6.5786	0.0070	0.0384	P4A- CMIT 4-P4A- BIT 150
Unknown 852	744.5325	4.9357	6.5722	0.0071	0.0384	P4A- CMIT 4-P4A- BIT 150
Unknown 1106	718.2859	7.3351	6.5338	0.0072	0.0391	P4A- CMIT 4-P4A- BIT 150
Unknown 771	411.3559	7.4940	6.5009	0.0073	0.0397	P4A- MIT 25-P4A- BIT 150
Unknown 1593	496.3127	2.3136	6.4942	0.0074	0.0397	P4A- MIT 25-P4A- CMIT 4
Unknown 394	447.2366	5.6187	6.4691	0.0075	0.0401	P4A- CMIT 4-P4A- BIT 150
Unknown 1268	514.2574	0.8509	6.4299	0.0076	0.0407	P4A- MIT 25-P4A- BIT 150
Unknown 1569	459.2254	1.4026	6.4281	0.0076	0.0407	P4A- MIT 25-P4A- BIT 150
Unknown 1063	627.5385	7.5824	6.4279	0.0076	0.0407	P4A- MIT 25-P4A- CMIT 4
Unknown 1057	770.5313	7.3893	6.4142	0.0077	0.0408	P4A- CMIT 4-P4A- BIT 150
Unknown 641	761.4974	8.1855	6.33	0.0081	0.0427	P4A- CMIT 4-P4A- BIT 150
Unknown 389	644.3403	6.7802	6.2949	0.0082	0.0433	P4A- CMIT 4-P4A- BIT 150
Unknown 1270	375.2748	3.2383	6.2931	0.0082	0.0433	P4A- Control-P4A- BIT 150
Unknown 358	692.5228	7.0386	6.2308	0.0085	0.0446	P4A- Control-P4A- MIT 25
Unknown 76	531.3679	6.6224	6.2086	0.0086	0.045	P4A- MIT 25-P4A- BIT 150
Unknown 1260	595.3187	7.0479	6.205	0.0087	0.045	P4A- MIT 25-P4A- BIT 150
Unknown 1065	200.0836	1.0919	6.1997	0.0087	0.045	P4A- CMIT 4-P4A- BIT 150
Unknown 695	274.1059	0.7795	6.1831	0.0088	0.0453	P4A- MIT 25-P4A- CMIT 4
Unknown 332	427.1257	3.2371	6.1472	0.0089	0.0461	P4A- CMIT 4-P4A- BIT 150
Unknown 970	589.3890	8.2222	6.1411	0.0090	0.0461	P4A- MIT 25-P4A- BIT 150
Unknown 533	642.2997	7.3434	6.1296	0.0090	0.0462	P4A- CMIT 4-P4A- BIT 150
Unknown 52	424.2482	6.4562	6.1024	0.0092	0.0468	P4A- Control-P4A- BIT 150
Unknown 591	477.3916	6.4455	6.0708	0.0093	0.0475	P4A- MIT 25-P4A- BIT 150
Unknown 732	241.1805	5.0552	6.0515	0.0094	0.0479	P4A- MIT 25-P4A- BIT 150
Unknown 250	332.1639	5.1331	6.0139	0.0097	0.0488	P4A- CMIT 4-P4A- BIT 150
Unknown 318	281.3012	7.2435	5.9988	0.0097	0.049	P4A- CMIT 4-P4A- BIT 150
Unknown 989	325.1987	5.4893	5.9896	0.0098	0.0491	P4A- MIT 25-P4A- BIT 150
Unknown 1176	364.1740	0.8410	5.9848	0.0098	0.0491	P4A- MIT 25-P4A- BIT 150
Unknown 403	607.2587	4.7110	5.9673	0.0099	0.0494	P4A- CMIT 4-P4A- BIT 150
Unknown 1326	277.1225	8.2280	5.9659	0.0099	0.0494	P4A- CMIT 4-P4A- BIT 150
Unknown 1102	277.1834	5.9508	5.9573	0.0100	0.0495	P4A- Control-P4A- BIT 150
Unknown 1079	810.4911	8.3596	5.9399	0.0101	0.0497	P4A- MIT 25-P4A- BIT 150
Unknown 1047	558.2574	0.9784	5.9396	0.0101	0.0497	P4A- MIT 25-P4A- BIT 150
